# Sputum and oral microbiota profiles are associated with long COVID severity and phenotype post-hospitalisation: a UK multicentre, prospective cohort study

**DOI:** 10.1183/13993003.01162-2026

**Published:** 2026-11-05

**Authors:** Daniela Alferes de Lima Headley, Hollian Richardson, Chandani Hennayake, Zsofia Eke, Jamie Stobo, Merete B. Long, Olivia C. Leavy, Matthew Richardson, Omer Elneima, Hamish J.C. McAuley, Aarti Shikotra, Amisha Singapuri, Marco Sereno, Ruth M. Saunders, Victoria C. Harris, Linzy Houchen-Wolloff, Neil J. Greening, Kimon Ntotsis, Daniel Lozano-Rojas, Beatriz Guillen-Guio, Ewen M. Harrison, Annemarie B. Docherty, Steven Kerr, Nazir I. Lone, Jennifer K. Quint, Aziz Sheikh, Mathew Thorpe, Bang Zheng, Claudia Efstathiou, Maxime Taquet, Iain D. Stewart, Trudie Chalder, Melanie J. Davies, Anthony De Soyza, John R. Geddes, William Greenhalf, Luke S. Howard, Joseph Jacob, R. Gisli Jenkins, William D-C. Man, Gerry P. McCann, Stefan Neubauer, Peter J.M. Openshaw, Joanna C. Porter, Matthew J. Rowland, David Thomas, Mark Toshner, Simon Heller, Janet T. Scott, Malcolm G. Semple, Sally J. Singh, Raminder Aul, Paul Beirne, Charlotte E. Bolton, Jeremy S. Brown, Gourab Choudhury, Nawar Diar Bakerly, Nicholas Easom, Carlos Echevarria, Jonathan Fuld, John R. Hurst, Mark Jones, Dhruv Parekh, Paul Pfeffer, Najib M. Rahman, Sarah Rowland-Jones, Ajay M. Shah, Daniel G. Wootton, Keir Lewis, Liam G. Heaney, Ling-Pei Ho, Alex Horsley, Michael Marks, Krisnah Poinasamy, Betty Raman, Louise V. Wain, Rachael A. Evans, Christopher E. Brightling, Philip L. Molyneaux, James D. Chalmers

**Affiliations:** 1 University of Dundee, Ninewells Hospital and Medical School, Dundee, UK; 2 Department of Population Health Sciences, University of Leicester, Leicester, UK; 3 The Institute for Lung Health, NIHR Leicester Biomedical Research Centre, University of Leicester, Leicester, UK; 4 NIHR Leicester Biomedical Research Centre, University of Leicester, Leicester, UK; 5 University Hospitals of Leicester NHS Trust, Leicester, UK; 6 Centre for Exercise and Rehabilitation Science, NIHR Leicester Biomedical Research Centre-Respiratory, University of Leicester, Leicester, UK; 7 Department of Respiratory Sciences, University of Leicester, Leicester, UK; 8 Therapy Department, University Hospitals of Leicester, NHS Trust, Leicester, UK; 9 Centre for Medical Informatics, The Usher Institute, University of Edinburgh, Edinburgh, UK; 10 London School of Hygiene and Tropical Medicine, London, UK; 11 Roslin Institute, University of Edinburgh, Edinburgh, UK; 12 The Usher Institute, University of Edinburgh, Edinburgh, UK; 13 Royal Infirmary of Edinburgh, NHS Lothian, Edinburgh, UK; 14 School of Public Health, Imperial College London, London, UK; 15 Nuffield Department of Primary Care Health Sciences, University of Oxford, Oxford, UK; 16 National Heart and Lung Institute, Imperial College London, London, UK; 17 Department of Psychiatry, University of Oxford, Oxford, UK; 18 Oxford Health NHS Foundation Trust, Oxford, UK; 19 Department of Psychological Medicine, Institute of Psychiatry, Psychology and Neuroscience, Kings College London, London, UK; 20 South London and Maudsley NHS Trust, London, UK; 21 Diabetes Research Centre, University of Leicester, Leicester, UK; 22 Population Health Sciences Institute, Newcastle University, Newcastle Upon Tyne, UK; 23 Newcastle upon Tyne Teaching Hospitals Trust, Newcastle upon Tyne, UK; 24 NIHR Oxford Health Biomedical Research Centre, University of Oxford, Oxford, UK; 25 Oxford Health NHS Foundation Trust, Oxford, UK; 26 University of Liverpool, Liverpool, UK; 27 The CRUK Liverpool Experimental Cancer Medicine Centre, Liverpool, UK; 28 Liverpool University Hospitals NHS Foundation Trust, Liverpool, UK; 29 Imperial College Healthcare NHS Trust, London, UK; 30 Imperial College London, London, UK; 31 Centre for Medical Image Computing, University College London, London, UK; 32 Lungs for Living Research Centre, University College London, London, UK; 33 Royal Brompton and Harefield Clinical Group, Guy's and St Thomas’ NHS Foundation Trust, London, UK; 34 NHLI, Imperial College London, London, UK; 35 King's Centre for Lung Health, Faculty of Life Sciences and Medicine, King's College London, London, UK; 36 Department of Cardiovascular Sciences, University of Leicester, Leicester, UK; 37 Division of Cardiovascular Medicine, Radcliffe Department of Medicine, University of Oxford, Oxford, UK; 38 UCL Respiratory, Department of Medicine, University College London, Rayne Institute, London, UK; 39 University College London Hospital (UCLH) NIHR Biomedical Research Centre, and ILD Service UCLH, London, UK; 40 Wellcome–Wolfson Institute for Experimental Medicine, Queen's University Belfast, Belfast, UK; 41 Cambridge NIHR Biomedical Research Centre, Cambridge, UK; 42 NIHR Cambridge Clinical Research Facility, Cambridge, UK; 43 Department of Oncology and Metabolism, University of Sheffield, Sheffield, UK; 44 MRC-University of Glasgow Centre for Virus Research, Glasgow, UK; 45 NIHR Health Protection Research Unit in Emerging and Zoonotic Infections, Institute of Infection, Veterinary and Ecological Sciences, University of Liverpool, Liverpool, UK; 46 Respiratory Medicine, Alder Hey Children's Hospital, Liverpool, UK; 47 St Georges University Hospitals NHS Foundation Trust, London, UK; 48 The Leeds Teaching Hospitals NHS Trust, Leeds, UK; 49 Centre for Respiratory Research, Translational Medical Sciences, School of Medicine, University of Nottingham, Nottingham, UK; 50 Nottingham University Hospitals NHS Trust, Nottingham, UK; 51 NIHR Nottingham Biomedical Research Centre, Nottingham, UK; 52 University of Edinburgh, Edinburgh, UK; 53 NHS Lothian, Edinburgh, UK; 54 Manchester Metropolitan University, Manchester, UK; 55 Salford Royal NHS Foundation Trust, Manchester, UK; 56 Infection Research Group, Hull University Teaching Hospitals, Hull, UK; 57 University of Hull, Hull, UK; 58 The Newcastle Upon Tyne Hospitals NHS Foundation Trust, Newcastle Upon Tyne, UK; 59 Translational and Clinical Research Institute, Newcastle University, Newcastle Upon Tyne, UK; 60 Department of Respiratory Medicine, Cambridge University Hospitals NHS Foundation Trust, Cambridge, UK; 61 University of Cambridge, Cambridge, UK; 62 University College London, London, UK; 63 Royal Free London NHS Foundation Trust, London, UK; 64 Clinical and Experimental Sciences, Faculty of Medicine, University of Southampton, Southampton, UK; 65 NIHR Southampton Biomedical Research Centre, University Hospitals Southampton, Southampton, UK; 66 University of Birmingham, Birmingham, UK; 67 University Hospital Birmingham NHS Foundation Trust, Birmingham, UK; 68 Barts Health NHS Trust, London, UK; 69 Queen Mary University of London, London, UK; 70 Oxford University Hospitals NHS Trust, Oxford, UK; 71 University of Oxford, Oxford, UK; 72 Oxford NIHR Biomedical Research Centre, Oxford, UK; 73 CAMS Oxford Institute, Oxford, UK; 74 University of Sheffield, Sheffield, UK; 75 Sheffield Teaching NHS Foundation Trust, Sheffield, UK; 76 Kings College London, London, UK; 77 Kings College London NHS Foundation Trust, London, UK; 78 Institute of Infection, Veterinary and Ecological Sciences, University of Liverpool, Liverpool, UK; 79 NIHR Health Protection Research Unit in Emerging and Zoonotic Infections, University of Liverpool, Liverpool, UK; 80 Hywel Dda University Health Board, Haverfordwest, UK; 81 University of Swansea, Swansea, UK; 82 Belfast Health and Social Care Trust, Belfast, UK; 83 MRC Human Immunology Unit, University of Oxford, Oxford, UK; 84 NIHR Oxford Biomedical Research Centre, Oxford, UK; 85 Division of Infection, Immunity and Respiratory Medicine, Faculty of Biology, Medicine and Health, University of Manchester, Manchester, UK; 86 Manchester University NHS Foundation Trust, Manchester, UK; 87 Department of Clinical Research, London School of Hygiene and Tropical Medicine, London, UK; 88 Hospital for Tropical Diseases, University College London Hospital, London, UK; 89 Division of Infection and Immunity, University College London, London, UK; 90 Asthma and Lung UK, London, UK; 91 Radcliffe Department of Medicine, University of Oxford, Oxford, UK; 92 Oxford University Hospitals NHS Foundation Trust, Oxford, UK

## Abstract

**Background:**

Acute COVID-19 alters the airway microbiota. Whether these changes persist post-COVID-19 and contribute to long COVID is unknown. We aimed to describe the microbiota by long COVID severity and subtype, and identify links with inflammation.

**Methods:**

Adults were prospectively recruited to the longitudinal UK Post-hospitalisation COVID-19 study (PHOSP-COVID). Patients were grouped by previously described “severity clusters” (of ongoing health impairments) and symptom subgroups. Oral and sputum microbiota (at 6 months post-discharge) were characterised through 16S ribosomal RNA gene amplicon sequencing, and microbiota diversity was assessed. Co-occurrence network analysis identified keystone taxa. Associations between keystone taxa and plasma inflammatory proteins were assessed through Spearman's rank correlations.

**Results:**

771 participants had their microbiota assessed. Of participants with oral rinses and “severity cluster” assignment (mean age 57.5 years), 278 out of 722 (38.5%) were women. The “very severe” cluster, in which disease was predominantly pulmonary in nature, was associated with a distinct microbiota profile post-COVID-19. Reduced lung function was associated with a distinct oral microbiota profile. Persistent neurocognitive symptoms were associated with a distinct oral microbiota profile, while gastrointestinal symptoms were associated with a distinct sputum microbiota profile. Microbiota keystone taxa shifted to a higher prevalence of lactic acid bacteria associated with worse health outcomes. Lactic acid bacteria abundance was associated with elevated blood inflammatory proteins.

**Conclusion:**

Distinct oral and sputum microbiota profiles were associated with pulmonary function and with neurocognitive and gastrointestinal symptoms in long COVID. Shifts in keystone microbiota taxa linked with inflammation, based on worse health outcomes, suggest a potential role change for lactic acid bacteria, which may be associated with the host immune response.

## Shareable abstract

**The post-COVID-19 airway microbiota is linked to long COVID severity and gastrointestinal and neurological symptoms. Keystone taxa shifts towards lactic acid bacteria are linked to inflammation, identifying airway microbiota changes as a feature of long COVID.** https://bit.ly/4vcBVYE

## Graphical abstract

**Figure:**
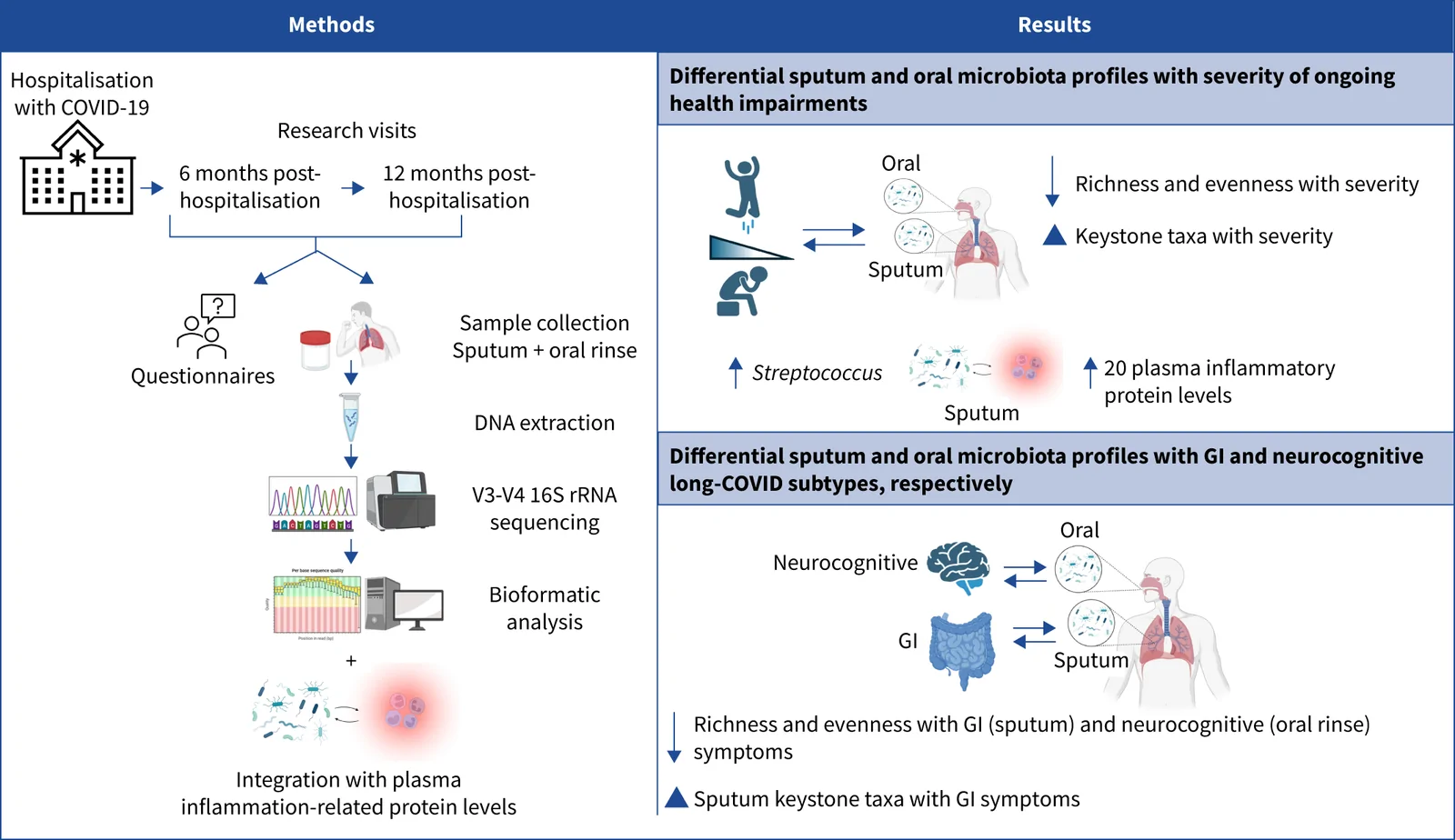
Overview of the study. GI: gastrointestinal.

## Introduction

SARS-CoV-2 infection may be associated with health impairments that persist for months after the initial illness, referred to as post-acute sequelae of SARS-CoV-2 infection, post-COVID-19 syndrome or long COVID (LC) [1]. Defined by the World Health Organization (WHO) as symptoms persisting or developing 3 months after initial infection and without an alternative explanation, LC affects multiple organs, including the respiratory, neurological, gastrointestinal (GI) and cardiovascular systems [2, 3]. Respiratory symptoms, such as dyspnoea and cough, are among the most frequent symptoms associated with LC and often referred to as pulmonary LC [4, 5]. The mechanisms of LC, including pulmonary LC, are not known, with severe symptoms not consistently associated with reduced pulmonary function or abnormal chest imaging [4–8]. The heterogeneity in LC presentation suggests complex and diverse underlying mechanisms, potentially involving systemic inflammation and microbial dysbiosis [9–13]. In view of the scale of LC, there is an urgent need to better understand potential mechanisms to guide identification of new treatment targets [14].

Emerging evidence indicates that the human microbiota, particularly in the oral cavity and lower airways, plays a critical role in health and disease. Sputum is a combination of upper and lower airway secretions and therefore can be thought of as an intermediate between the upper and lower airway microbiota, while the oral and nasal microbiota shape the respiratory microbiota through microaspiration [15, 16]. Acute COVID-19 is a pulmonary disease and the infection phase is known to alter the oral and airway microbiota, with shifts in microbial composition linked to disease severity and immune responses [17, 18]. These microbial communities are not isolated; they interact with other organ systems through communication pathways such as the gut–lung and the oral–brain axes, which have been associated with other chronic diseases [19–21]. However, the medium- and long-term effects of COVID-19 on these ecosystems and their potential role in the sustained multi-organ symptoms of LC remain poorly understood [22]. Persistent changes in the oral and airway microbiota could potentially contribute to ongoing health impairments through these multi-organ communication pathways.

The Post-hospitalisation COVID-19 (PHOSP-COVID) study, a large multicentre prospective cohort in the UK, provides a unique opportunity to assess these associations in individuals recovering from severe COVID-19 requiring hospitalisation. While most patients with COVID-19 were not hospitalised, the risk of ongoing health impairment is known to be related to the severity of initial illness and is greater in individuals with severe disease requiring hospitalisation [14, 23]. Previous analyses from this cohort have identified distinct health impairment phenotypes and inflammatory profiles associated with LC, suggesting a multifaceted pathophysiology [10, 14, 24]. We hypothesised that the oral and sputum microbiota may exhibit distinct microbial profiles post-COVID-19 linked to the severity of ongoing health impairments and LC symptom subgroups.

In this study, we aimed to 1) characterise the oral and sputum microbiotas at a median of 6 months post-hospitalisation in a subset of PHOSP-COVID participants; 2) investigate associations between microbiota composition and recovery status, ongoing health impairment clusters and specific LC symptom subgroups; and 3) explore potential links between microbial shifts and systemic inflammation.

## Methods

### Study design, participants and ethics

The PHOSP-COVID cohort is a multicentre prospective cohort study that has been previously described [14, 25]. In brief, the study recruited patients ≥18 years old who were discharged from one of 83 National Health Service (NHS) hospitals in the UK between 10 August 2020 and 31 March 2022, following admission due to PCR-confirmed or clinician-diagnosed COVID-19.

A subset of participants provided biological samples in addition to clinical data during research visits [25]. Spontaneous sputum and oral rinse samples, alongside environmental controls, were collected at median 6 months and 12 months post-hospitalisation. The first available sample for each participant was included for this analysis unless otherwise stated (*i.e.* a 12-month visit sample was only included when the corresponding 6-month visit sample was not available).

Symptom data from patient-reported outcome measures were collected at research visits. Patients were categorised into those with ongoing symptoms believed to be related to COVID-19 (LC) and those without ongoing symptoms. Among those with ongoing symptoms, patient's symptom patterns were grouped into subgroups of GI, cardiopulmonary, fatigue, neurocognitive and mental health based on having at least one symptom in each of these categories. Patients could be included in more than one subgroup (supplementary table S1) [24]. Patient-reported outcome measures were derived from questionnaires with predefined thresholds for symptom group assignment, as detailed in supplementary table S1. For downstream analysis, patients were classified as either belonging or not belonging to each symptom subgroup (supplementary table S8). Recovery is a binary measure, and previous research suggests it is crucial to take into account the severity of LC symptoms. To do this we used a previous unsupervised cluster analysis classification of severity clusters by ongoing health impairment at 6 months post-hospitalisation [14]. The four severity clusters are “mild”, “moderate/cognitive”, “severe” and “very severe”. The mild cluster was predominantly male, had the fewest symptoms with fewer comorbidities, and 42.7% of participants reported feeling fully recovered. The moderate/cognitive cluster was predominantly older and male; had moderate disability, fatigue and breathlessness; had fewer comorbidities; and 36.4% of participants reported feeling fully recovered. The severe cluster was associated with comorbidities; severe disability, fatigue and breathlessness; and 7.0% of participants reported feeling fully recovered. The very severe cluster was associated with comorbidities; very severe disability, fatigue and breathlessness; and 2.7% of participants reported feeling fully recovered. As previously described, the dominant symptoms leading to disability in the severe and very severe clusters were breathlessness and fatigue, with or without other symptoms [14]. For this analysis, only 6 months post-hospitalisation research visit samples were included.

Written informed consent was obtained from all patients. The study was approved by the Leeds West Research Ethics Committee (20/YH/0225) and is registered on the ISRCTN Registry (ISRCTN10980107).

### DNA extraction and sequencing

Sputum and oral rinse DNA were extracted using the DNeasy PowerSoil Pro Kit (Qiagen Cat. No. 47016). Sequencing of the V3–V4 region of 16S ribosomal RNA (rRNA) was performed on an Illumina MiSeq platform. DNA extraction controls, oral wash environmental controls and sequencing controls were included in the analysis to determine experimental background contamination. Full details on sequencing, quality control, pre-processing, decontamination and amplicon sequence variant generation are described in the supplementary methods. Sequencing data have been submitted to European Nucleotide Archive (project accession number PRJEB86010).

### Microbiota analysis

Microbiota and statistical analysis were performed using R version 4.4.2 (www.r-project.org). Alpha diversity was compared between mild and all other severity clusters using linear regression, with adjustments for age, sex, body mass index (BMI), number of comorbidities, presence of respiratory comorbidities, antibiotic treatment during hospitalisation, smoker status, WHO clinical progression score and sequencing read depth. Microbiota alpha diversity was measured using Shannon–Wiener Diversity index, with consistency assessed after repeating with Chao1 and Simpson indices, using R package “phyloseq” (version 1.48.0). A similar approach was used for the LC subgroups, with the corresponding comorbidity class (*i.e.* cardiovascular, neuropsychiatric or GI) included in the adjustments. No adjustment for multiple comparisons was performed when conducting the pre-planned comparisons of severity clusters and comorbidity groups.

Beta diversity was assessment by PERMANOVA based on weighted UniFrac using R package “vegan” (version 2.6–6.1), with the same factor adjustments as alpha diversity excluding sequencing read depth. Principal coordinate analysis was based on weighted UniFrac dissimilarity of microbiome using R package “phyloseq” (version 1.48.0).

Differential relative abundance analysis was performed using three methods, random forest (a classifier), MicrobiotaProcess package and DESeq2. Agreement by at least two methods was required to ensure robustness of findings. Full details, including multiple comparison adjustments, are described in the supplementary methods.

### Co-occurrence network

In health, the microbiota is composed of a community of organisms that interact and form stable associations. These associations can be affected by external influences (such as viral infections), and lead to disease and dysbiosis [26]. Co-occurrence networks were used to identify keystone taxa (taxa that play an important role in the stability of the network) and to assess the association between taxa dependent on clinical outcomes. Analysis was performed using the SPIEC-EASI signed network method applied by R package NetCoMi (version 1.1.0). Full details are described in the supplementary methods.

### Inflammatory protein assays and over-representation analysis

Previous PHOSP-COVID analysis found an association between very severe ongoing health impairment and symptom groups with systemic inflammation post-COVID-19 [10, 24]. Microbiota profile changes have been associated with systemic diseases and inflammation. To explore whether there is a link between microbiota shifts and systemic inflammation, the correlation of microbiota keystone taxa relative abundance and plasma inflammatory proteins was examined. Plasma samples were analysed for inflammation-related protein levels through an Olink Explore 384 Inflammation panel as previously described [24]. Over-representation analysis was used to identify biological process pathways over-represented by the identified microbiome-associated inflammatory proteins. Full details are presented in the supplementary methods.

### Role of the funding source

The funder of the study had no role in study design, data collection, data analysis, data interpretation or writing of the report.

## Results

2697 patients discharged after hospitalisation with COVID-19 between 10 August 2020 and 31 March 2022 provided biological samples and/or additional clinical data prospectively [25]. Of these, 1891 provided oral rinse samples and 364 provided sputum samples. In total, there were 3030 oral rinse and 521 sputum samples provided (figure 1). Following quality control, 771 participants were included for analysis of the oral microbiota, and 327 participants were included for analysis of the sputum microbiota (figure 1). Characteristics of the patients included are shown in table 1.

**FIGURE 1:**
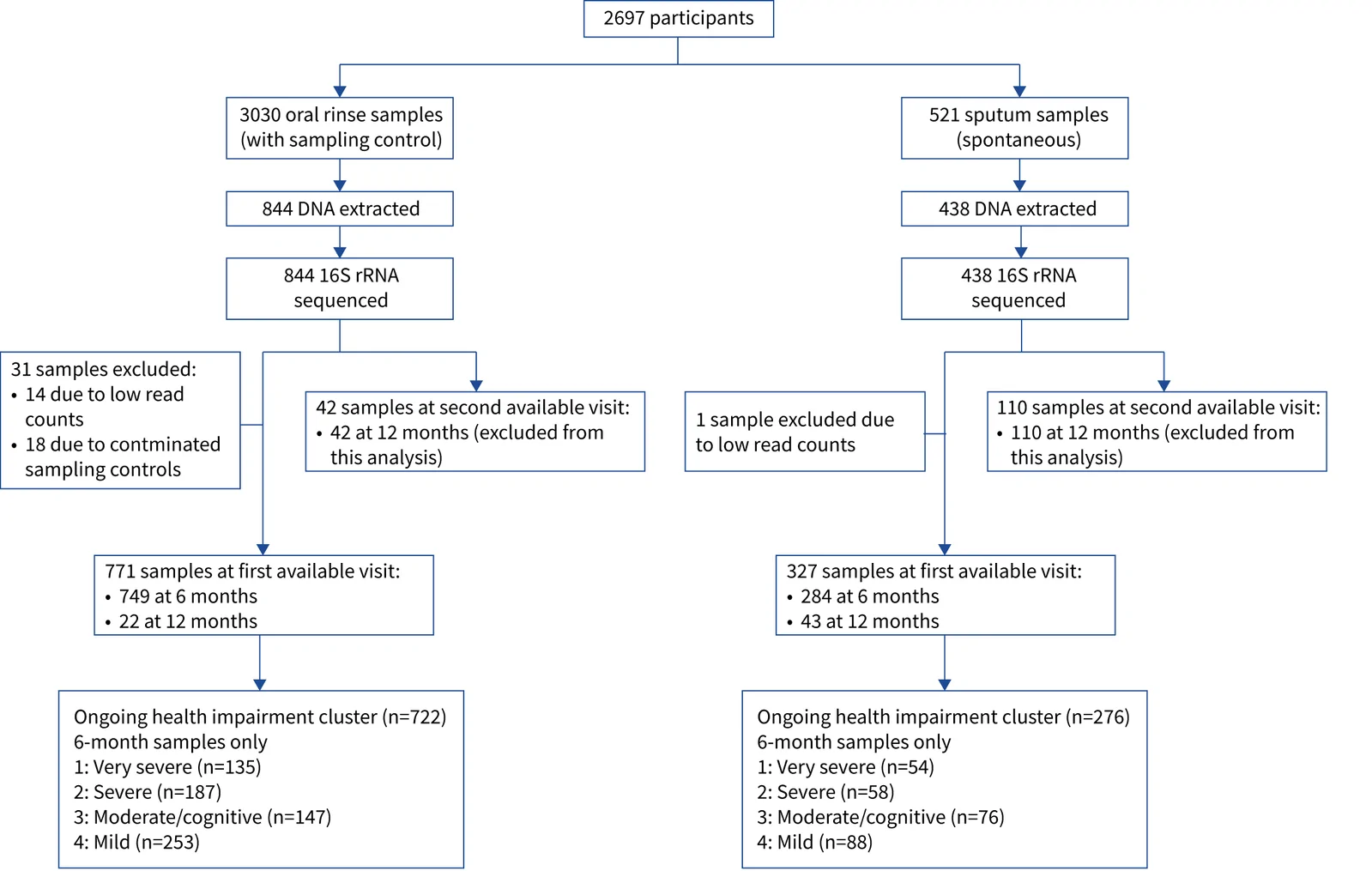
Flow diagram of oral and sputum sample processing for microbiota analysis.

**TABLE 1:** Participant demographics, clinical characteristics and illness severity stratified by sample type and ongoing health impairment cluster at 6 months post-hospitalisation

	Oral rinse samples^#^					Sputum samples^¶^				
	Very severe	Severe	Moderate/cognitive	Mild	Total	Very severe	Severe	Moderate/cognitive	Mild	Total
**Participants (n)**	135	187	147	253	722	54	58	76	88	276
**Age (years)**	54.3±11.4	54.8±11.3	65.3±12.1	56.6±12.5	57.5±12.6*	52.4±11.8	57.4±11.3	64.4±10.6	54.3±14.2	57.3±13
**Sex**										
Female	69 (51.1)	86 (46.0)	47 (32.0)	76 (30.0)	278 (38.5)***	28 (51.9)	24 (41.4)	22 (29.0)	27 (30.7)	101 (36.6)*
Male	66 (48.9)	101 (54.0)	100 (68.0)	177 (70.0)	444 (61.5)	26 (48.2)	34 (58.6)	54 (71.1)	61 (69.3)	175 (63.4)
**BMI (kg·cm^−2^)**	33.9±7.0	33.8±7.6	30.2±5.0	30.6±6.0	32±6.7***	34.2±8.7	31.7±6.7	31.2±5.6	30.3±7.5	31.6±7.2**
Missing (n)	15	26	22	42	105	12	12	18	21	63
**Comorbidities (n)**	3.4±3.0	2.1±2.1	2.3±2.1	1.5±1.5	2.2±2.2***	2.4±2.3	1.6±1.9	2.1±2.0	1.6±1.6	1.9±2*
**Comorbidities**										
Respiratory^+^	65 (48.2)	48 (25.7)	30 (20.4)	66 (26.1)	209 (28.9)***	26 (48.2)	15 (25.9)	11 (14.5)	20 (22.7)	72 (26.1)*
Cardiovascular	58 (43.0)	76 (40.6)	80 (54.4)	99 (39.1)	313 (43.4)	22 (40.7)	18 (31.0)	44 (57.9)	35 (39.8)	119 (43.1)
Neuro-psychiatric	74 (54.8)	46 (24.6)	11 (7.5)	16 (6.3)	147 (20.4)***	18 (33.3)	8 (13.8)	7 (9.2)	11 (12.5)	44 (15.9)**
Rheumatology	23 (17.0)	23 (12.3)	17 (11.6)	17 (6.7)	80 (11.1)**	4 (7.4)	7 (12.1)	10 (13.2)	5 (5.7)	26 (9.4)
Gastrointestinal	19 (14.1)	37 (19.8)	17 (11.6)	16 (6.3)	89 (12.3)*	5 (9.3)	10 (17.2)	9 (11.8)	12 (13.6)	36 (13.0)
** **Type 2 diabetes	25 (18.5)	35 (18.7)	40 (27.2)	42 (16.6)	142 (19.7)	10 (18.5)	7 (12.1)	21 (27.6)	13 (14.8)	51 (18.5)
**Hospital stay (days), median (IQR)**	11 (4–26)	8 (3–18)	10 (5–25)	8 (3–16)	9 (4–20)	8 (4–22.8)	11 (5.3–18.5)	7.5 (4.8–11.3)	9 (4.8–19)	8 (4–16.26)
**Treated with antibiotic**	107 (79.3)	147 (78.6)	129 (87.8)	207 (81.8)	590 (82.5)	41 (75.9)	41 (70.7)	62 (81.6)	68 (77.3)	212 (81.5)
Missing (n)	2	2	0	5	9	5	4	0	7	16
**WHO clinical progression scale** ^§^										
Class 3–4	30 (22.2)	49 (26.2)	20 (13.6)	45 (17.8)	144 (19.9)	8 (14.8)	9 (15.5)	7 (9.2)	11 (12.5)	35 (12.7)
Class 5	39 (28.9)	47 (25.1)	55 (37.4)	98 (38.7)	239 (33.1)	20 (37.0)	18 (31.0)	39 (51.3)	33 (37.5)	110 (39.9)
Class 6	25 (18.5)	40 (21.4)	29 (19.7)	49 (19.4)	143 (19.8)	13 (24.1)	11 (19.0)	14 (18.4)	19 (21.6)	57 (20.7)
Class 7–9	41 (30.4)	51 (27.3)	43 (29.3)	61 (24.1)	196 (27.1)	13 (24.1)	20 (34.5)	16 (21.1)	25 (28.4)	74 (26.8)
**Smoking status**										
Non-smoker	73 (54.1)	96 (51.3)	62 (42.9)	148 (58.5)	380 (59.2)	34 (63.0)	32 (55.2)	43 (56.6)	52 (59.1)	161 (61.9)
Ex-smoker	43 (31.9)	69 (36.9)	61 (41.5)	73 (28.9)	246 (38.3)	19 (35.2)	18 (31.0)	30 (39.5)	29 (33.0)	96 (36.9)
Current smoker	4 (3.0)	5 (2.7)	2 (1.4)	5 (2.0)	16 (2.5)	0 (0.00)	3 (5.2)	0 (0.00)	0 (0.00)	3 (1.2)
Missing (n)	15	17	21	27	80	1	5	3	7	16
**FEV_1_ % predicted**										
<80	21 (45.7)	12 (16.7)	17 (25.8)	20 (18.5)	70 (24.0)	11 (37.9)	7 (21.2)	11 (28.9)	9 (18.0)	38 (25.3)
>80	25 (54.3)	60 (83.3)	49 (74.2)	88 (81.5)	222 (76)	18 (62.1)	26 (78.8)	27 (71.1)	41 (82.0)	112 (74.7)
Missing (n)	89	115	81	145	430	25	25	38	38	126
**FVC % predicted**										
<80	25 (54.3)	13 (18.3)	20 (30.3)	21 (19.4)	79 (27.1)	13 (44.8)	8 (24.2)	14 (36.8)	13 (26.0)	48 (32.0)
>80	21 (45.7)	58 (81.7)	46 (69.7)	87 (80.6)	212 (72.9)	16 (55.2)	25 (75.8)	24 (63.2)	37 (74.0)	102 (68.0)
Missing (n)	89	116	81	145	431	25	25	38	38	126

Data are presented as mean±sd or n (%), unless otherwise indicated. Percentages were calculated per variable with missing data excluded. For categorical variables, chi-squared or Fisher's test were performed as appropriate. For continuous variables, Mann–Whitney tests were performed. BMI: body mass index; FEV_1_: forced expiratory volume in 1 s; FVC: forced vital capacity; IQR: interquartile range; WHO: World Health Organization. ^#^: n=722; ^¶^: n=276; ^+^: details of respiratory comorbidities can be found in supplementary table S15; ^§^: WHO clinical progression scale: 3–4=no continuous supplemental oxygen needed; 5=continuous supplemental oxygen only; 6=continuous or bi-level positive airway pressure ventilation or high-flow nasal oxygen; 7–9=invasive mechanical ventilation or other organ support. *: p<0.05; **: p<0.01; ***: p<0.0001.

### Distinct oral and sputum microbiota profiles are associated with very severe ongoing health impairment

All alpha diversity statistics were adjusted for age, sex, BMI, number of comorbidities, presence of respiratory comorbidities, antibiotic treatment during hospitalisation, smoker status, WHO clinical progression score and sequencing read depth. All beta diversity statistics had the same factor adjustments as alpha diversity, excluding sequencing read depth.

There was no difference in microbiota diversity or composition based on the broad definition of recovery for either microbiota (supplementary figure S1). However, antibiotic usage was associated with reduced sputum microbiota diversity (supplementary figure S2).

For the oral microbiota, all alpha diversity indices were statistically significantly lower (p≤0.001–0.03; estimate= −0.12 (Shannon–Wiener), −0.02 (Simpson), −3.57 (Chao1 diversity index)) in the very severe health impairment cluster than in the mild cluster (figure 2a–c), indicating a reduction in richness and evenness with health impairment severity at 6 months (median) post-COVID-19 hospitalisation. Microbial composition was also found to vary between health impairment cluster (p=0.01, r^2^=0.01) (figure 2d). Differential abundance analysis with agreement by at least two methods identified a reduction in relative abundance with severity for 10 genera, including four oral commensals: *Prevotella*, *Neisseria*, *Haemophilus* and *Fusobacterium*; and an increase in relative abundance with severity for other commensal oral genera: *Streptococcus*, *Rothia*, *Veillonella* and *Limosilactobacillus* (figure 2e).

**FIGURE 2:**
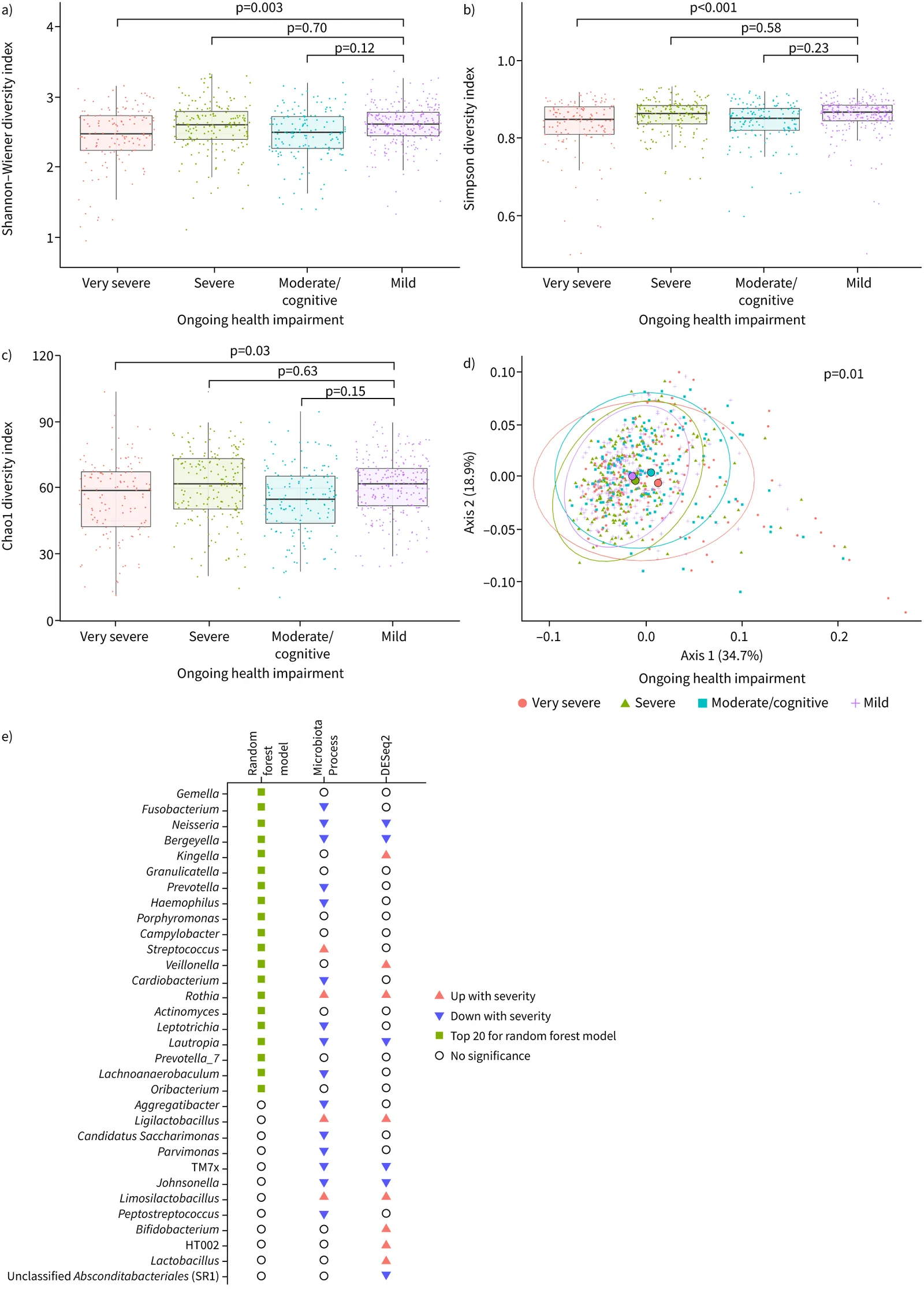
Oral microbiota diversity based on participant ongoing health impairment post-COVID-19 hospitalisation. a) Shannon–Wiener diversity index (alpha diversity). b) Simpson diversity index (alpha diversity). c) Chao1 diversity index (alpha diversity). d) Weighted UniFrac principal coordinates analysis plot comparing ongoing health impairment clusters. Large bold circle represents centroid of group. e) Differential abundance analysis summary of three methods: random forest model, MicrobiotaProcess and DESeq2.

Results of the sputum microbiota supported the findings in the oral microbiota. The Shannon–Wiener and Simpson diversity indices were statistically significantly lower (p=0.01–0.04, estimate= −0.12 (Shannon–Wiener), −0.03 (Simpson diversity index)) in the very severe cluster than in the mild cluster (figure 3a, b). The Chao1 diversity index was significantly lower (p=0.03, estimate= −3.70) in the mild cluster than in the severe cluster (figure 3c). This indicates a reduction in richness and evenness of genus abundance with very severe health impairments, but an increase in differential taxa with a severe health impairment at 6 months (median) post-COVID-19 hospitalisation. Microbial composition did not vary significantly between health impairment clusters (p=0.42, r^2^=0.01) (figure 3d). Differential abundance analysis with agreement by at least two methods did not identify differences for any genera (figure 3e).

**FIGURE 3:**
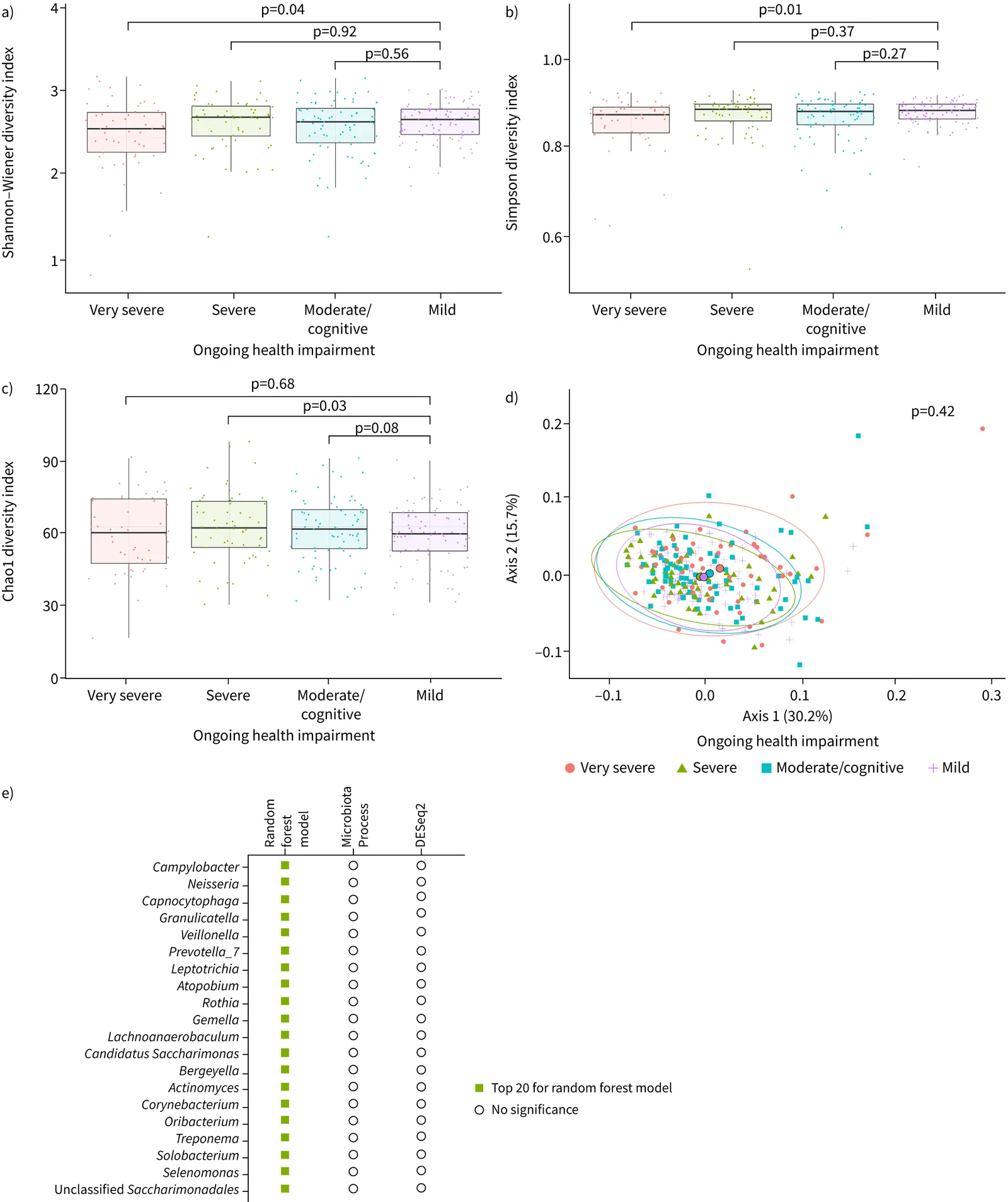
Sputum microbiota diversity based on participant ongoing health impairment post-COVID-19 hospitalisation. a) Shannon–Wiener diversity index (alpha diversity). b) Simpson diversity index (alpha diversity). c) Chao1 diversity index (alpha diversity). d) Weighted UniFrac principal coordinates analysis plot comparing ongoing health impairment clusters. Large bold circle represents centroid of group. e) Differential abundance analysis summary of three methods: random forest model, MicrobiotaProcess and DESeq2.

Keystone taxa differed between the mild and very severe clusters for both microbiotas (figure 4a, b). For the oral microbiota, the mild cluster keystone taxa were two core commensal taxa: *Veillonella* and *Streptococcus*; for the very severe cluster, the keystone taxa were *Rothia* and the less abundant (in their corresponding network) lactic acid bacteria (LAB) *Lactobacillus* and *Limosilactobacillus* (figure 4a). Co-occurrence networks were dissimilar in terms of centrality measurements (p=0.01–0.02) (supplementary table S4a). Full centrality measurements comparisons can be found in supplementary table S4b.

**FIGURE 4:**
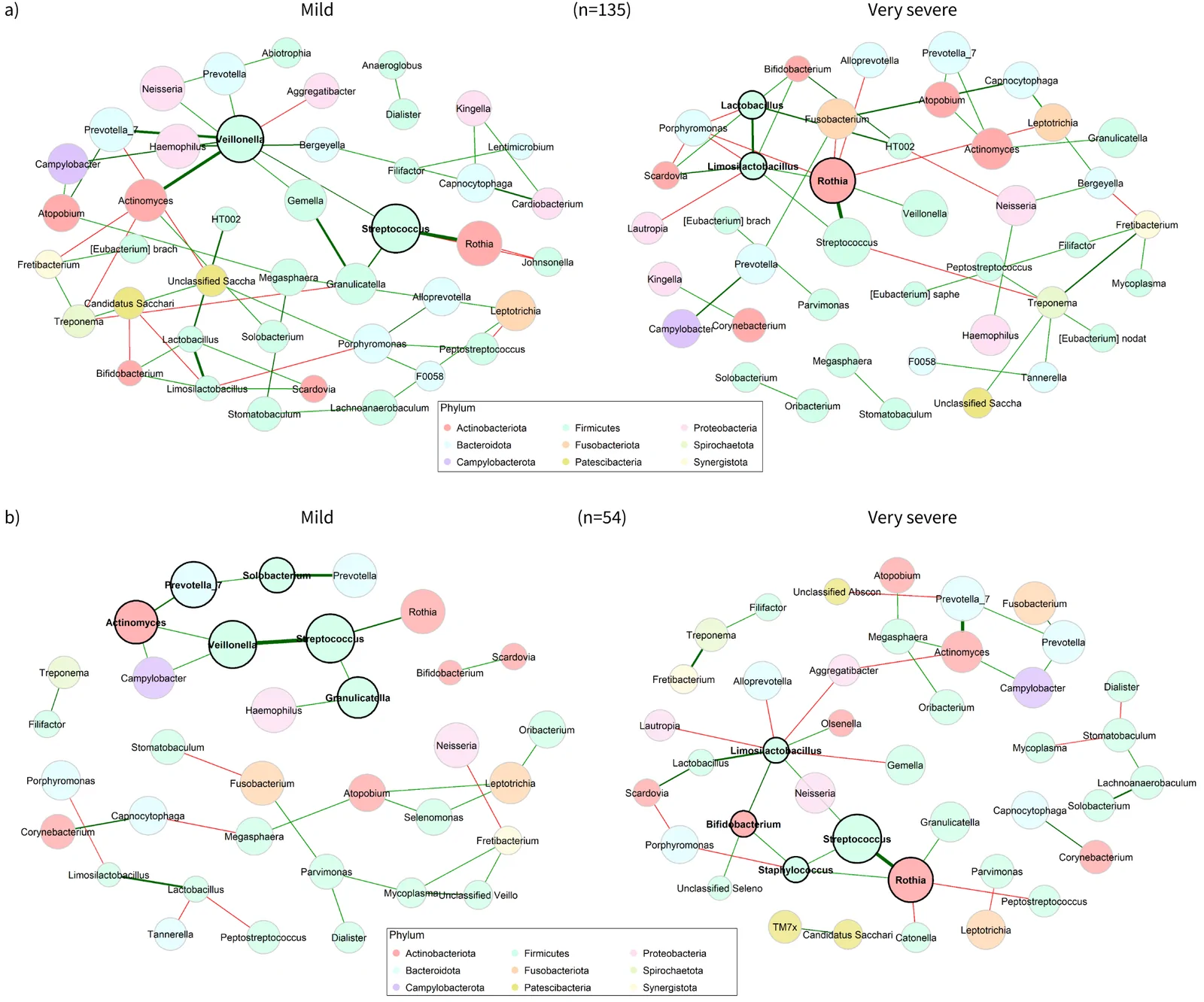
Oral (a) and sputum (b) microbiota co-occurrence network comparison based on participant ongoing health impairment post-COVID-19 hospitalisation. SPIEC-EASI method used for association measures. Green edges correspond to positive estimated association and red edges to negative ones. Edge weight corresponds to estimated correlations. Keystone taxa are highlighted by bold text and borders. Node size corresponds to its centred log-ratio normalised counts. Node colours represent phylum.

For the sputum microbiota, the mild cluster had an abundance of core commensal keystone taxa such as *Actinomyces*, *Prevotella* and *Streptococcus*, while the very severe cluster retained *Streptococcus* but shifted to *Rothia* and the less abundant (in their corresponding network) LAB *Bifidobacterium* and *Limosilactobacillus* (figure 4b). There was a loss of association of *Streptococcus* with other core commensal taxa in the very severe cluster, with a stronger association (edge weight) with *Rothia* and new associations including with *Staphylococcus*. The reduction in core commensal keystone taxa and change in associations for *Streptococcus* suggest a potential shift in behaviour by the taxa. The co-occurrence networks were dissimilar based on centrality measurements (p=0.02). In addition, the mild cluster network had a higher positive edge percentage (p=0.04), which is linked to more stable networks (supplementary table S5a). Full centrality measurements comparisons can be found in supplementary table S5b.

For the oral microbiota (101 samples), the relative abundance of *Lactobacillus* was positively correlated with plasma interleukin (IL)-17A and the relative abundance of *Streptococcus* was positively correlated with plasma C-type lectin domain family 4 member D (CLEC4D) (supplementary table S6). For the sputum microbiota (37 samples), the relative abundance of *Streptococcus* was positively correlated with 20 plasma inflammatory proteins (figure 5a, supplementary table S7). These were colony-stimulating factor-1 (CSF1), basigin (BSG), IL17D, matrilin-2 (MATN2), nucleoside diphosphate kinase-3 (NME3), CCL21, lymphotoxin β receptor (LTBR), CD58, creatine kinase U-type, mitochondrial 1A and 1B (CKMT1A_CKMT1B), IL17C, tumour necrosis factor receptor superfamily member 11b (TNFRSF11B), regenerating family member 4 (REG4), CXCL14, CCL25, CXCL17, transforming growth factor-α (TGFA), aldehyde dehydrogenase 3 family member A1 (ALDH3A1), calsyntenin-2 (CLSTN2), Fc α-receptor (FCAR) and IL33.

**FIGURE 5:**
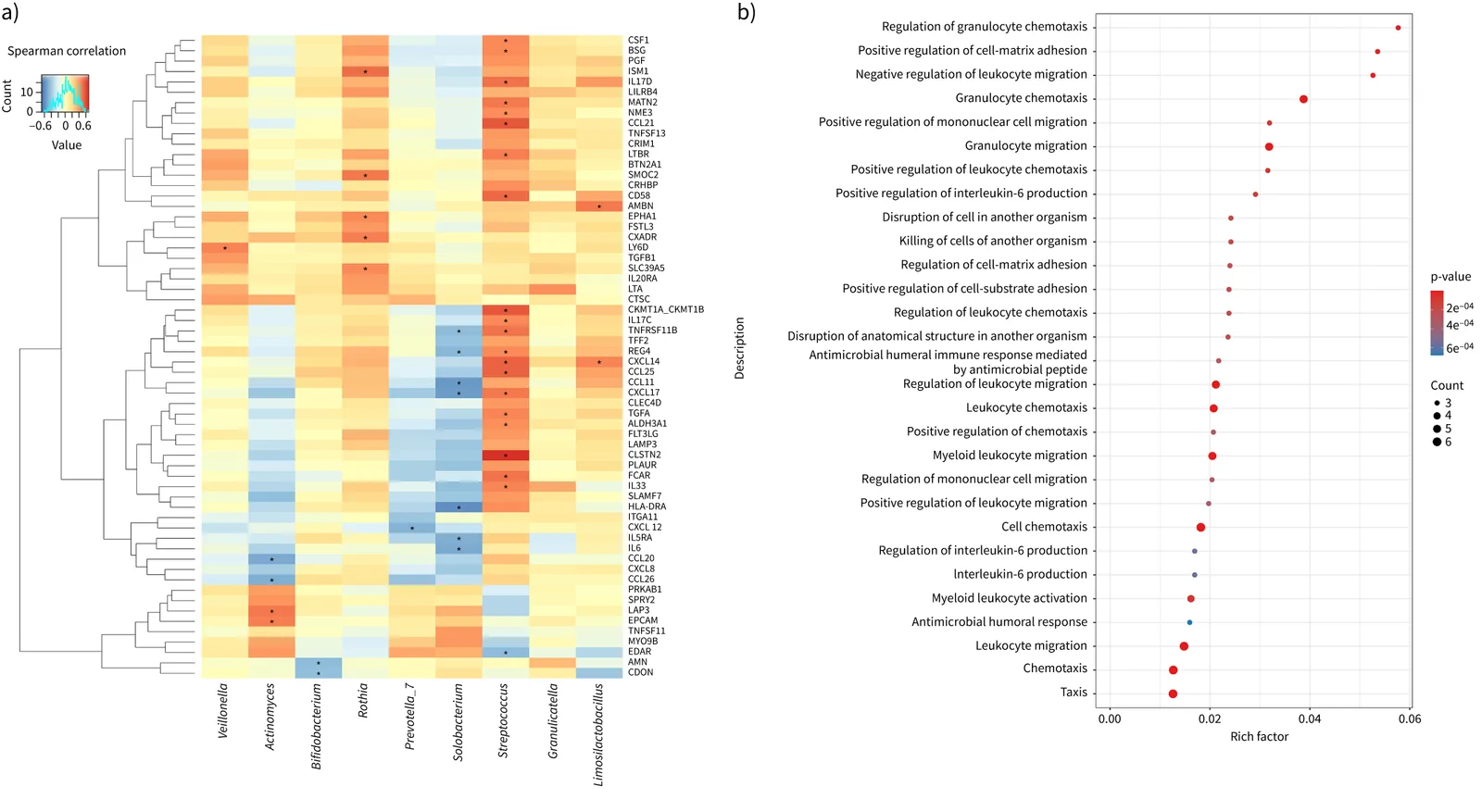
a) Correlation between sputum microbiota in patients with very severe ongoing health impairment and inflammation-related proteins. A Spearman rank correlation analysis for the co-occurrence network keystone taxa with inflammation-related proteins levels (NPX), from Olink Explore 384 Inflammation panel. *: p<0.05 (FDR adjusted) and absolute r^2^≥0.4. b) Over-representation analysis of the 20 inflammatory proteins with *Streptococcus* for the very severe ongoing health impairment group. Biological processes used a p-value cut-off of 0.01 (Benjamini–Hochberg adjusted), and a minimum count of three proteins.

An over-representation analysis was performed for the identified microbiome-associated inflammatory proteins. This identified pathways linked to chemotaxis; leukocyte production, migration and regulation; IL6 activation and regulation; and antimicrobial response (figure 5b).

### Oral and sputum microbiota are associated with long COVID subgroups

All alpha diversity statistics were adjusted for age, sex, BMI, number of comorbidities, presence of respiratory comorbidities, antibiotic treatment during hospitalisation, smoker status, WHO clinical progression score, sequencing read depth and presence of relevant pre-existing comorbidities (GI pre-existing comorbidities for the GI symptoms subgroup, neurological and psychiatric pre-existing comorbidities for the neurocognitive and mental health symptoms subgroup, cardiovascular pre-existing comorbidities for the cardiopulmonary symptoms subgroup). All beta diversity statistics had the same factor adjustments as alpha diversity, excluding sequencing read depth.

Participants with ongoing neurocognitive symptoms had a less rich oral microbiota (p=0.02, estimate= −0.06) and a composition that differed from participants without these symptoms (p=0.01, r^2^=0.006) (figure 6a, c). Participants with fatigue had an oral microbiota composition that differed from participants without these symptoms (p=0.04, r^2^=0.004) (figure 6c). Participants with GI symptoms had a less rich sputum microbiota (p=0.003, estimate= −0.16) and a composition that differed compared to participants without these symptoms (p=0.03, r^2^=0.010) (figure 6b, d).

**FIGURE 6:**
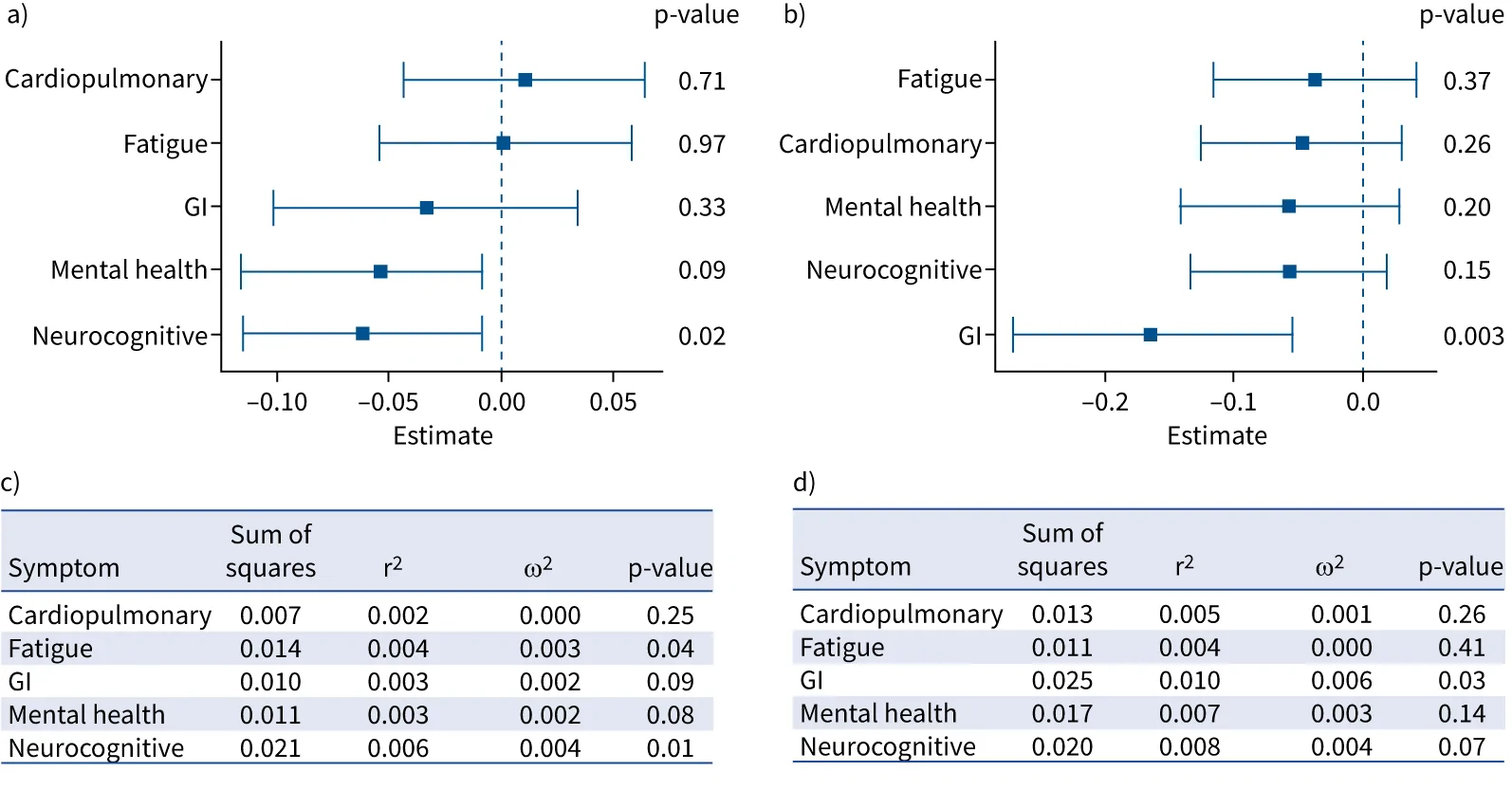
Oral and sputum microbiota comparison based on presence of long-term symptoms post-COVID-19 hospitalisation. a) Forest plot of estimate with 95% confidence interval of Shannon–Wiener diversity index (alpha diversity) for oral microbiome. Comparison between participants with symptoms and participants without symptoms or recovered. b) Forest plot of estimate with 95% confidence interval of Shannon–Wiener diversity index (alpha diversity) for sputum microbiota. Comparison between participants with symptoms and participants without symptoms or recovered. c) Table displaying weighted UniFrac summary PERMANOVA result for oral microbiota. Comparison between participants with symptoms and participants without symptoms. d) Table displaying weighted UniFrac summary PERMANOVA result for sputum microbiota. Comparison between participants with symptoms and participants without symptoms. GI: gastrointestinal.

Microbiota associations dependent on the LC symptom subgroup were investigated through co-occurrence network analysis. For the oral microbiota in the presence and absence of neurocognitive symptoms, two keystone taxa were shared: *Actinomyces* and *Treponema*. In addition, in the absence of symptoms, the following taxa were keystone taxa: *Veillonella*, *Granullicatella* and *Bergeyella.* While in the presence of symptoms, the following were keystone taxa: *Streptococcus*, *Scardovia* and *Lactobacillus* (figure 7a, supplementary table S9a). Full centrality measurements comparisons can be found in supplementary table S9b.

**FIGURE 7:**
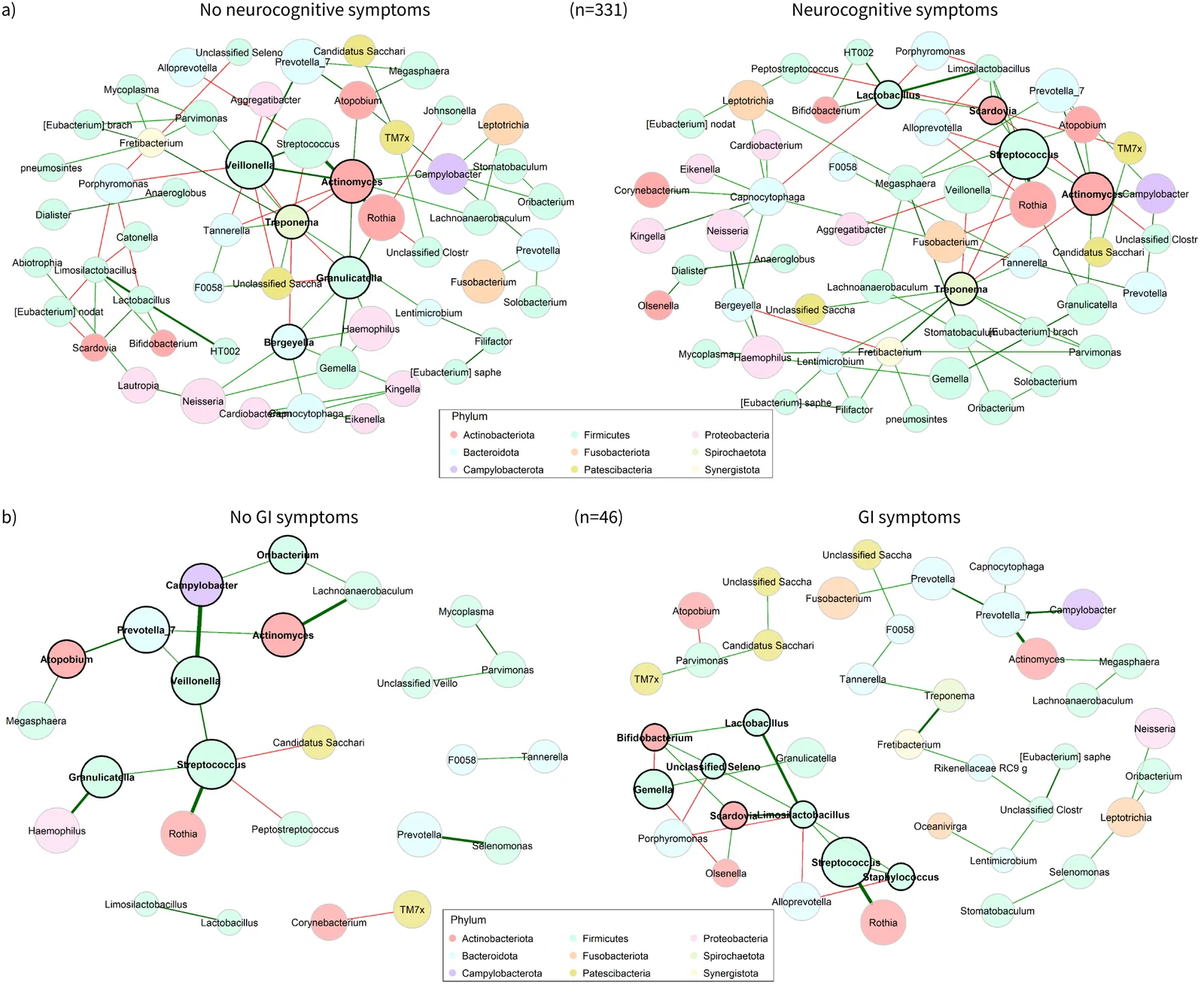
a) Oral microbiota co-occurrence network comparison based on ongoing neurocognitive symptoms post-COVID-19 hospitalisation. b) Sputum microbiota co-occurrence network comparison based on ongoing gastrointestinal (GI) symptoms post-COVID-19 hospitalisation. SPIEC-EASI method used for association measures. Green edges correspond to positive estimated association and red edges to negative ones. Edge weight corresponds to estimated correlations. Keystone taxa are highlighted by bold text and borders. Node size corresponds to its centred log-ratio normalised counts. Node colours represent phylum.

For the sputum microbiota in the absence of GI symptoms, the keystone taxa were mostly commensal taxa, such as *Streptococcus*, *Actinomyces*, *Veillonella* and *Prevotella_7*. By contrast, in the presence of GI symptoms, only *Streptococcus* was a shared keystone taxon, all other taxa were less abundant (in their corresponding network), such as *Limosilactobacillus*, *Bifidobacterium*, *Lactobacillus* and *Staphylococcus* (figure 7b)*.* The co-occurrence networks were dissimilar based on centrality measurements (p=0.01–0.02) (supplementary table S10a). Full centrality measurements comparisons can be found in supplementary table S10b.

No significant associations were found for the oral microbiota neurocognitive symptoms group and systemic inflammation. However, *Streptococcus* and *Lactobacillus* were positively correlated with five and four inflammatory proteins, respectively, for the sputum microbiota GI symptoms group (26 samples) (supplementary figure S11 and table S11).

### Oral microbiota is associated with lung function

All alpha diversity statistics were adjusted for age, sex, BMI, number of comorbidities, presence of respiratory comorbidities, antibiotic treatment during hospitalisation, smoker status, WHO clinical progression score and sequencing read depth. All beta diversity statistics had the same factor adjustments as alpha diversity, excluding sequencing read depth.

For the oral microbiota, Chao1 diversity index was significantly lower (p=0.03, estimate= −4.13; p=0.04, estimate= −4.02) in the forced expiratory volume in 1 s (FEV_1_) and forced vital capacity (FVC) <80% predicted groups, respectively (supplementary figures S13c and S14c). This indicates a reduction in differential taxa with reduced lung function. Microbial composition was significantly different between the FEV_1_ and FVC <80% and >80% predicted groups (p=0.03, r^2^=0.008; p=0.02, r^2^=0.009) (supplementary figures S13d and S14d). Differential abundance analysis with agreement by at least two methods identified a reduction in *Aggregatibacter*, *Candidatus Saccharimonas*, *Lachnoanaerobaculum* and *Megasphaera* with FEV_1_ <80% predicted; and a reduction in *Megasphaera, C. Saccharimonas, Oribacterium* and *Stomatobaculum* and an increase in *Capnocytophaga* with FVC <80% predicted (supplementary figures S13e and S14e).

There was no difference in the sputum microbiota diversity or composition based on FEV_1_ % and FVC % predicted being less or more than 80% (supplementary figures S15 and S16).

## Discussion

We observed distinct oral and sputum microbiota profiles associated with very severe ongoing health impairments and with LC subgroups. These findings provide insight into the contribution of the respiratory microbiota to pulmonary LC. The “very severe” ongoing health impairments cluster in this cohort had the highest frequency of breathlessness, fatigue, reduced lung function and markedly reduced exercise capacity on incremental shuttle walk test, consistent with pulmonary LC with and without involvement of other organ systems [14]. Our results demonstrate a relationship between distinct oral and sputum microbiota profiles and the severity of pulmonary and overall disability in patients with LC. In addition, distinct sputum microbiota profiles were observed for patients with GI symptoms. Co-occurrence network analysis identified a difference in *Streptococcus* behaviour with very severe ongoing health impairments and GI symptoms in sputum. *Streptococcus* abundance in the groups with more severe health impairments was associated with plasma inflammatory proteins, suggesting a link between local microbiota profile differences and systemic inflammation. Our results highlight a potential role of LAB-driven microbiota in worse health outcomes post-COVID-19.

Most patients in our cohort had symptoms across multiple organ systems, consistent with observations in other LC studies, but it is notable that the severe and very severe ongoing health impairment groups were dominated by pulmonary LC [14, 27, 28]. On average, patients with very severe ongoing health impairment had 20 distinct symptoms, but they had markedly increased dyspnoea (Dyspnoea-12 score 18.2 *versus* 1.6 in the mild group), fatigue (FACIT score 34.9 *versus* 7.0), reduced incremental shuttle walk distance (278 m *versus* 536 m) and lower lung function parameters (FEV_1_ <80% predicted: 49.1% *versus* 22.4%; FVC <80% predicted: 55.6% *versus* 26.3%) [14]. Owing to the multisystem nature of LC, it is not possible within the PHOSP data to study a “pure” pulmonary group; however, reduced lung function parameters (FEV_1_ % predicted and FVC % predicted) were associated with differential oral microbiota profiles and the strongest associations in our data are with the group with the highest pulmonary morbidity (very severe ongoing health impairment group).

In the oral microbiota, very severe ongoing health impairment was associated with an increase in abundance of the oral commensals *Streptococcus*, *Rothia*, *Veillonella* and *Limosilactobacillus* and a decrease in *Neisseria*, *Prevotella*, *Haemophilus* and *Fusobacterium* compared with mild impairment. These taxa have been reported as being differently abundant in several oral microbiota studies of acute COVID-19; observed discordances likely reflect differences in study design (supplementary table S12). The overlap between our findings and these studies at the time of infection suggest a possible medium-term sustained pulmonary microbiota alteration post-COVID-19. This corroborates the findings of Cui *et al.* [29] in a small study that compared the microbiota of patients with COVID-19 *versus* healthy controls at hospital discharge and 1 year later, in which they observed a lack of full normal oral microbiota restoration. The decrease in *Neisseria* was consistently identified [18, 29–32]. Furthermore, Wu *et al*. [18] found that a decrease in *Neisseria* in patients with COVID-19 coincided with a depletion of the tricarboxylic acid (TCA) cycle, which is linked to microbiome dysbiosis. The existing literature provide evidence that airway *Neisseria* is depleted during acute COVID-19 and that the persistent decrease in *Neisseria* is associated with long-term, severe, ongoing symptoms post-COVID-19.

Across oral and sputum microbiota, LAB (*Limosilactobacillus*, *Lactobacillus*, *Bifidobacterium* and *Streptococcus* (in sputum)) were identified as keystone taxa in poor outcome networks. It is important to note that not all species within these genera may contribute to the observed shifts. *Streptococcus* and taxa of the Lactobacillales order have been identified as core members of the oral microbiota [33, 34]. However, *Bifidobacterium* is primarily associated with the gut microbiota and is not considered a core member of the oral microbiota, although this taxon has been reported in relation to dental caries [33–37]. The presence of these taxa, typically associated with the gut and oral microbiota, was unexpected in sputum and we speculate that this could be due to “leaky gut” or potentially reflux followed by microaspiration [38–40]. It is important to note that this interpretation represents a hypothesis based on observational data, and mechanistic studies would be required to confirm the origin and functional role of these taxa. LAB are facultative anaerobes and do not use the full TCA cycle, suggesting a potential microbiota metabolic activity shift, although microbial functional profiling would be necessary to confirm this hypothesis. A plausible cause for a preference for facultative anaerobes is lung mucosal hypoxia, which has been linked to inflammation or infection [41–43]. Activity of inflammatory cells such as neutrophils can lead to localised oxygen depletion *via* reactive oxygen species production and increasing hypoxia-inducible gene transcription and responses in epithelial cells [44].

In line with potential inflammatory cell influence on the sputum microbiota, the relative abundance of *Streptococcus* in very severe ongoing health impairment was associated with inflammatory proteins involved in chemotaxis and in leukocyte production, migration and regulation (figure 3d). Excessive inflammatory cell recruitment can cause tissue damage and chronic inflammation [45]. The elevated proteins CSF1, IL33, FCAR (CD89), CCL21, IL17D and CCL25 have been associated with COVID-19 severity, mortality and prolonged COVID-19 [46–53]. CCL25 has been associated with gut homing and REG4 (RELP) is associated with GI inflammation, further suggesting gut–lung axis crosstalk [52, 54]. TNFRSF11B has been linked with neuroinflammatory processes and with LC severity; it was also elevated in the LC cognitive group in a previous PHOSP-COVID study [24, 55, 56]. TGFA, a growth factor associated with lung fibrosis after lung injury, was linked with very severe ongoing health impairment and several LC subgroups in previous PHOSP-COVID studies [10, 24, 57]. MATN2, involved in the recruitment of immune cells, was linked with all LC subgroups in a prior PHOSP-COVID study [24, 58]. Interestingly, we identified an over-representation of proteins involved in IL6 activation and regulation, although not significantly elevated itself. IL6 was elevated in those with very severe ongoing health impairments in a prior PHOSP-COVID study [10].

In the oral microbiota, *Lactobacillus* was associated with elevated IL17A and *Streptococcus* was associated with elevated CLEC4D for those with very severe ongoing health impairment. Elevated IL17A has been associated with COVID-19 severity and is thought to play a role in tissue damage [59]. CLEC4D was also found to be elevated with very severe ongoing health impairments in a prior PHOSP-COVID study [10]. The lack of association of the oral microbiota with systemic inflammation for neurocognitive symptoms is partially due to our threshold for correlation significance of 0.4 (for moderate or above correlations) and potentially indicates a role for non-inflammation-based oral–brain crosstalk [60].

In line with our findings, prior studies have also reported associations between LC and changes in oral and respiratory microbiota [61–63]. Although methodologies differed between studies, changes included dysbiosis, enrichment of inflammation-associated taxa and differences in microbial metabolism [61–63].

Although causality cannot be inferred with this study, both microbiota profile differences and chronic inflammation appear to be key components of ongoing COVID-19. Similar to bronchiectasis, a form of vicious cycle may exist in which inflammation drives microbiota profile shifts, which in turn drive inflammation [64]. Mechanistic studies are needed to better understand the microbiota–inflammation relationship to ascertain whether this could be a viable therapeutic target in LC.

There are limitations to our study. Our cohort lacked a non-COVID-19 control group, making it difficult to ascertain whether the microbiota differences observed are specific to COVID-19 or due to general respiratory illness and/or hospitalisation [29–31, 65]. However, the taxa shifts identified here were previously observed in acute COVID-19 studies using non-COVID-19 control groups (hospitalised or healthy). No baseline samples were available, so we could not establish whether the microbiota differences preceded or were a consequence of COVID-19. Antibiotic use during hospitalisation was associated with reduced microbiota diversity, and although this was accounted for through covariant adjustment, patients may have received different antibiotic treatments for different durations during the 6-month period, which is difficult to fully account for. Taxa assignment was only possible to genus level, so species-specific behaviour remains unclear. Sputum samples were only available from patients with ongoing production, which is an indicator of worse health and potential respiratory comorbidity. However, respiratory comorbidities for both microbiotas are comparable (table 1), suggesting that the majority of individuals with available sputum samples had pulmonary LC. The group size for the microbiota and inflammatory proteins correlation analysis was sometimes small (26–101 samples) and therefore should be validated in a larger study. The cause of associations between worse health outcomes, inflammation and microbiota profile differences could not be ascertained and should be investigated by further mechanistic studies to identify potential therapeutic targets. Importantly, our hospitalised cohort may not fully capture the spectrum of LC, particularly milder disease. Disease severity during infection was associated with microbiota profile differences (supplementary figure S3). However, this was not associated with the ongoing health impairment cluster in our participant subset (table 1) and was accounted for in our analysis through covariant adjustment.

In conclusion, we found ongoing health impairment (dominated by pulmonary LC) was associated with differential oral and sputum microbiota profiles post-COVID-19. Reduced FEV_1_ % predicted and FVC % predicted were associated with a differential oral profile post-COVID-19. Associations were found for neurocognitive and GI symptoms with oral and sputum microbiota, respectively, post-COVID-19. Differences in keystone taxa within sputum/oral microbiota, and their link to inflammation based on worse health outcomes, suggest a potential role change for LAB, which may be associated with host immune responses.

## Data Availability

The protocol, consent form, definition and derivation of clinical characteristics and outcomes, training materials, regulatory documents, information about requests for data access, and other relevant study materials are available online.

## Supplementary material

**Please note:** supplementary material is not edited by the Editorial Office, and is uploaded as it has been supplied by the author. Supplementary material ERJ-01162-2026.Supplement
