# Supplementary material for: Sputum and oral microbiota profiles are associated with long COVID severity and phenotype post-hospitalisation: a UK multicentre, prospective cohort study
Source: Eur Respir J. 2026 Nov 5;68(5):2601162. doi: 10.1183/13993003.01162-2026 (PMC13735185; doi:10.1183/13993003.01162-2026)
Supplement: Supplementary file 1 [file ERJ-01162-2026.Supplement.pdf]

Supplement

Contents

The PHOSP-COVID collaborative group 1
Supplementary Methods 11
Supplementary Data - Results 13
Figures 13
Tables 25
References 59

The PHOSP-COVID collaborative group

Writing Group

Daniela Alferes de Lima Headley[1], Hollian Richardson[1], Chandani Hennayake[1], Zsofia Eke[1], Jamie Stobo[1], Merete B Long[1], Olivia C Leavy[2,3], Matthew Richardson[3], Omer Elneima[3], Hamish J C McAuley[3], Aarti Shikotra[4], Amisha Singapuri[3], Marco Sereno[3], Ruth M Saunders[3], Victoria C Harris[3,5], Linzy Houchen-Wolloff[6,7,8], Neil J Greening[3], Kimon Ntotsis[3], Daniel Lozano-Rojas[3], Beatriz Guillen-Guio[2,3], Ewen M Harrison[9], Annemarie B Docherty[9], Steven Kerr[9,10], Nazir I Lone[11,12], Jennifer K Quint[13], Aziz Sheikh[14],Mathew Thorpe[9], Bang Zheng[15], Claudia Efstathiou[16], Maxime Taquet[17,18], Iain D Stewart[16], Trudie Chalder[19,20], Melanie J Davies[4,5,21], Anthony De Soyza[22,23], John R Geddes[24,25],William Greenhalf[26,27,28],Luke S Howard[29,30], Joseph Jacob[31,33], R Gisli Jenkins[16], William D-C Man[34,35,36], Gerry P McCann[4,37], Stefan Neubauer[24,38], Peter JM Openshaw[16], Joanna C Porter[39,40], Matthew J Rowland[41], David Thomas[30], Mark Toshner[42,43], Simon Heller[44], Janet T Scott[45], Malcolm G Semple[46,47], Sally J Singh[3], Raminder Aul[48], Paul Beirne[49], Charlotte E Bolton[50,51,52],Jeremy S Brown[39], Gourab Choudhury[53,54], Nawar Diar Bakerly[55,56], Nicholas Easom[57,58], Carlos Echevarria[59,60], Jonathan Fuld[43,61,62], John R Hurst[32,63], Mark Jones[64,65], Dhruv Parekh[66,67], Paul Pfeffer[68,69], Najib M Rahman[70,71,72,73], Sarah Rowland-Jones[74,75], Ajay M Shah[76,77], Daniel G Wootton[28,78,79], Keir Lewis[80,81], Liam G Heaney[41,82], Ling-Pei Ho[83,84], Alex Horsley[85,86], Michael Marks[87,88,89], Krisnah Poinasamy[90], Betty Raman[91,92], Louise V Wain[2,3], Rachael A Evans[3,5], Christopher E Brightling[3], Philip L Molyneaux[16], James D Chalmers[1,91] on behalf of the PHOSP-COVID Collaborative group

1. University of Dundee, Ninewells Hospital and Medical School, Dundee, UK
2. Department of Population Health Sciences, University of Leicester, Leicester, UK
3. The Institute for Lung Health, NIHR Leicester Biomedical Research Centre, University of Leicester, Leicester, UK
4. NIHR Leicester Biomedical Research Centre, University of Leicester, Leicester, UK
5. University Hospitals of Leicester NHS Trust, Leicester, UK
6. Centre for Exercise and Rehabilitation Science, NIHR Leicester Biomedical Research Centre-Respiratory, University of Leicester, Leicester, UK
7. Department of Respiratory Sciences, University of Leicester, Leicester, UK
8. Therapy Department, University Hospitals of Leicester, NHS Trust, Leicester, UK
9. Centre for Medical Informatics, The Usher Institute, University of Edinburgh, Edinburgh, UK
10. London School of Hygiene & Tropical Medicine, London, UK
11. Roslin Institute, University of Edinburgh, Edinburgh, UK
12. The Usher Institute, University of Edinburgh, Edinburgh, UK
13. Royal Infirmary of Edinburgh, NHS Lothian, Edinburgh, UK
14. School of Public Health, Imperial College London, London, UK
15. Nuffield Department of Primary Care Health Sciences, University of Oxford, Oxford, UK
16. National Heart and Lung Institute, Imperial College London, London, UK
17. Department of Psychiatry, University of Oxford, Oxford OX3 7JX, UK
18. Oxford Health NHS Foundation Trust, Oxford OX3 7JX, UK
19. Department of Psychological Medicine, Institute of Psychiatry, Psychology and Neuroscience, Kings College London, London, UK
20. South London and Maudsley NHS Trust, London, UK
21. Diabetes Research Centre, University of Leicester, Leicester, UK
22. Population Health Sciences Institute, Newcastle University, Newcastle Upon Tyne, UK
23. Newcastle upon Tyne Teaching Hospitals Trust, Newcastle upon Tyne, UK
24. NIHR Oxford Health Biomedical Research Centre, University of Oxford, Oxford, UK
25. Oxford Health NHS Foundation Trust, Oxford, UK
26. University of Liverpool, Liverpool, UK
27. The CRUK Liverpool Experimental Cancer Medicine Centre, Liverpool, UK
28. Liverpool University Hospitals NHS Foundation Trust, Liverpool, UK
29. Imperial College Healthcare NHS Trust, London, UK
30. Imperial College London, London, UK
31. Centre for Medical Image Computing, University College London, London, UK
32. University College London, London, UK
33. Lungs for Living Research Centre, University College London, London, UK
34. Royal Brompton and Harefield Clinical Group, Guys and St Thomas NHS Foundation Trust, London, UK
35. NHLI, Imperial College London, London, UK
36. King’s Centre for Lung Health, Faculty of Life Sciences & Medicine, King’s College London
37. Department of Cardiovascular Sciences, University of Leicester, Leicester, UK

38. Division of Cardiovascular Medicine, Radcliffe Department of Medicine, University of Oxford, Oxford, UK
39. UCL Respiratory, Department of Medicine, University College London, Rayne Institute, London, UK
40. University College London Hospital (UCLH) NIHR BRC, and ILD Service UCLH, London, UK
41. Wellcome-Wolfson Institute for Experimental Medicine, Queen's University Belfast, Belfast, UK
42. Cambridge NIHR BRC, Cambridge, UK
43. NIHR Cambridge Clinical Research Facility, Cambridge, UK
44. Department of Oncology and Metabolism, University of Sheffield, Sheffield, UK
45. MRC-University of Glasgow Centre for Virus Research, Glasgow, UK
46. NIHR Health Protection Research Unit in Emerging and Zoonotic Infections, Institute of Infection, Veterinary and Ecological Sciences, University of Liverpool, UK
47. Respiratory Medicine, Alder Hey Children's Hospital, Liverpool, UK
48. St Georges University Hospitals NHS Foundation Trust, London, UK
49. The Leeds Teaching Hospitals NHS Trust, Leeds, UK
50. Centre for Respiratory Research, Translational Medical Sciences, School of Medicine, University of Nottingham, Nottingham, UK
51. Nottingham University Hospitals NHS Trust, Nottingham, UK
52. NIHR Nottingham Biomedical Research Centre, Nottingham, UK
53. University of Edinburgh, Edinburgh, UK
54. NHS Lothian, Edinburgh, UK
55. Manchester Metropolitan University, Manchester, UK
56. Salford Royal NHS Foundation Trust, Manchester, UK
57. Infection Research Group, Hull University Teaching Hospitals, Hull, UK
58. University of Hull, Hull, UK
59. The Newcastle Upon Tyne Hospitals NHS Foundation Trust, Newcastle Upon Tyne, UK
60. Translational and Clinical Research Institute, Newcastle University, Newcastle Upon Tyne, UK
61. Department of Respiratory Medicine, Cambridge University Hospitals NHS Foundation Trust, Cambridge, UK
62. University of Cambridge, Cambridge, UK
63. Royal Free London NHS Foundation Trust, London, UK
64. Clinical and Experimental Sciences, Faculty of Medicine, University of Southampton, Southampton, UK
65. NIHR Southampton Biomedical Research Centre, University Hospitals Southampton, Southampton, UK
66. University of Birmingham, Birmingham, UK
67. University Hospital Birmingham NHS Foundation Trust, Birmingham, UK
68. Barts Health NHS Trust, London, UK
69. Queen Mary University of London, London, UK
70. Oxford University Hospitals NHS Trust, Oxford, UK
71. University of Oxford, Oxford, UK
72. Oxford NIHR Biomedical Research Centre, Oxford, UK
73. CAMS Oxford Institute, Oxford, UK
74. University of Sheffield, Sheffield, UK
75. Sheffield Teaching NHS Foundation Trust, Sheffield, UK
76. Kings College London, London, UK
77. Kings College London NHS Foundation Trust, London, UK
78. Institute of Infection, Veterinary and Ecological Sciences, University of Liverpool, Liverpool, UK
79. NIHR Health Protection Research Unit in Emerging and Zoonotic Infections, University of Liverpool, Liverpool, UK
80. Hywel Dda University Health Board, Wales, UK
81. University of Swansea, Wales, UK
82. Belfast Health & Social Care Trust, Belfast, UK
83. MRC Human Immunology Unit, University of Oxford, Oxford, UK
84. NIHR Oxford Biomedical Research Centre, Oxford, UK
85. Division of Infection, Immunity & Respiratory Medicine, Faculty of Biology, Medicine and Health, University of Manchester, Manchester, UK
86. Manchester University NHS Foundation Trust, Manchester, UK
87. Department of Clinical Research, London School of Hygiene & Tropical Medicine, London, UK
88. Hospital for Tropical Diseases, University College London Hospital, London, UK
89. Division of Infection and Immunity, University College London, London, UK

90. Asthma and Lung UK, London, UK
91. Radcliffe Department of Medicine, University of Oxford, Oxford, UK
92. Oxford University Hospitals NHS Foundation Trust, Oxford, UK

Core Management Group

Chief Investigator C E Brightling, *Members* R A Evans (Lead Co-I), L V Wain (Lead Co-I), J D Chalmers, V C Harris, L P Ho, A Horsley, M Marks, K Poinasamy, B Raman, A Shikotra, A Singapuri

PHOSP-COVID Study Central Coordinating Team

C E Brightling (Chief Investigator), R A Evans (*Lead Co-I*), L V Wain (*Lead Co-I*), R Dowling, C Edwardson, O Elneima, S Finney, N J Greening, B Guillen-Guio, B Hargadon, V C Harris, L Houchen--Wolloff, O C Leavy, D Lozano-Rojas, H J C McAuley, K Ntotsis, C Overton, T Plekhanova, R M Saunders, M Sereno, A Singapuri, A Shikotra, C Taylor, S Terry, C Tong, B Zhao,

Steering Committee

Co-chairs D Lomas, E Sapey, *Institution representatives* C Berry, C E Bolton, N Brunskill, E R Chilvers, R Djukanovic, Y Ellis, D Forton, N French, J George, N A Hanley, N Hart, L McGarvey, N Maskell, H McShane, M Parkes, D Peckham, P Pfeffer, A Sayer, A Sheikh, A A R Thompson, N Williams and core management group representation

Executive Board

Chair C E Brightling, representation from the core management group, each working group and platforms

Platforms

Bioresource

W Greenhalf (*Co-Lead*), M G Semple (*Co-Lead*), M Ashworth, H E Hardwick, L Lavelle-Langham, W Reynolds, M Sereno, R M Saunders, A Singapuri, V Shaw, A Shikotra, B Venson, L V Wain

Data Hub

A B Docherty (*Co-Lead*), E M Harrison (*Co-Lead*), A Sheikh (*Co-Lead*), J K Baillie, C E Brightling, L Daines, C M Efstathiou, R A Evans, R Free, L Gardiner, A F Goemans, B Guillen-Guio, M M Iqbal, S Kerr, O C Leavy, F Liew, N I Lone, D Lozano-Rojas, H J C McAuley, K Ntotsis, R Pius, J Quint, M Richardson, , M Sereno, I D Stewart, M Taquet, M Thorpe, L V Wain, L Wang.

Genetic Analysis

L V Wain (Co-Lead), J K Baillie (*Co-Lead*), N Avramidis, E Coughlan, B Guillen-Guio, O C Leavy, E Pairo-Castineira, K Rawlik

Imaging Alliance

M Halling-Brown (*Co-Lead*), F Gleeson (*Co-Lead*), J Jacob (*Co-Lead*), S Neubauer (*Co-Lead*) B Raman (*Co-Lead*) S Siddiqui (*Co-Lead*) J M Wild (*Co-Lead*), S Aslani, G Baxter, M Beggs, C Bloomfield, M P Cassar, A Chiribiri, E Cox, D J Cuthbertson, M Halling-Brown, V M Ferreira, L Finnigan, S Francis, P Jezzard, G J Kemp, H Lamlum, E Lukaschuk, C Manisty, G P McCann , C McCracken, K McGlynn , R Menke , C A Miller , A J Moss, T E Nichols, C Nikolaidou , C O'Brien , G Ogbole, B Rangelov, D P O'Regan , A Pakzad, S Piechnik , S Plein, I Propescu, A A Samat, L Saunders, Z B Sanders, R Steeds, T Treibel, E M Tunnicliffe, M Webster, J Willoughby, J Weir McCall, C Xie, M Xu

Omics

L V Wain (*Co-Lead),* J K Baillie (*Co-Lead*), H Baxendale, C E Brightling, M Brown, J D Chalmers, R A Evans, B Gooptu, W Greenhalf, H E Hardwick, R G Jenkins, D Jones, I Koychev, C Langenberg, A Lawrie, P L Molyneaux, A Shikotra, J Pearl, M Ralser, N Sattar, R M Saunders, J T Scott, T Shaw, D Thomas, D Wilkinson

Working Groups

Airways

L G Heaney (*Co-Lead*), A De Soyza (*Co-Lead*), D Adeloye, C E Brightling, J S Brown, J Busby, J D Chalmers, C Echevarria, L Daines, O Elneima, RA Evans, J Hurst, P Novotny, C Nicolaou, P Pfeffer, K Poinasamy, J Quint, I Rudan, E Sapey, M Shankar-Hari, A Sheikh, S Siddiqui, S Walker, B Zheng

Brain

J R Geddes (*Lead*), M Hotopf *(Co-Lead)*, K Abel, R Ahmed, L Allan, C Armour, D Baguley, D Baldwin, C Ballard, K Bhui, G Breen, K Breeze, M Broome, T Brugha, E Bullmore, D Burn, F Callard, J Cavanagh, T Chalder, D Clark, A David, B Deakin, H Dobson, B Elliott, J Evans, RA Evans, R Francis, E Guthrie, P Harrison, M Henderson, A Hosseini, N Huneke, M Husain, T Jackson, I Jones, T Kabir, P Kitterick, A Korszun, I Koychev, J Kwan, A Lingford-Hughes, P Mansoori, H McAllister-Williams, K McIvor, B Michael, L Milligan, R Morriss, E Mukaetova-Ladinska, K Munro, A Nevado-Holgado, T Nicholson, C Nicolaou, S Paddick, C Pariante, J Pimm, K Saunders, M Sharpe, G Simons, J P Taylor, R Upthegrove, S Wessely

Cardiac

G P McCann (*Lead*), S Amoils, C Antoniades, A Banerjee, A Bularga, C Berry, P Chowienczyk, J P Greenwood, A D Hughes, K Khunti, C Lawson, N L Mills, A J Moss, S Neubauer, B Raman, A N Sattar, C L Sudlow, M Toshner,

Immunology

P J M Openshaw (*Lead*), D Altmann, J K Baillie, R Batterham, H Baxendale, N Bishop, C E Brightling, P C Calder, C M Efstathiou, R A Evans, J L Heeney, T Hussell, P Klenerman, F Liew, J M Lord, P Moss, S L Rowland-Jones, W Schwaeble, M G Semple, R S Thwaites, L Turtle, L V Wain, S Walmsley, D Wraith

Intensive Care

M J Rowland (*Lead*), A Rostron (*Co-Lead*), J K Baillie, B Connolly, A B Docherty, N I Lone, D F McAuley, D Parekh, A Rostron, J Simpson, C Summers

Lung Fibrosis

R G Jenkins (*Co-Lead*), J Porter (*Co-Lead*), R J Allen, R Aul, J K Baillie, S Barratt, P Beirne, J Blaikley, R C Chambers, N Chaudhuri, C Coleman, E Denneny, L Fabbri, P M George, M Gibbons, F Gleeson, B Gooptu, B Guillen-Guio, I Hall, N A Hanley, L P Ho, E Hufton, J Jacob, I Jarrold, G Jenkins, S Johnson, M G Jones, S Jones, F Khan, P Mehta, J Mitchell, P L Molyneaux, J E Pearl, K Piper Hanley, K Poinasamy, J Quint, D Parekh, P Rivera-Ortega, L C Saunders, M G Semple, J Simpson, D Smith, M Spears, L G Spencer, S Stanel, I D Stewart, A A R Thompson, D Thickett, R Thwaites, L V Wain, S Walker, S Walsh, J M Wild, D G Wootton, L Wright

Metabolic

S Heller (*Co-Lead*), M J Davies (*Co-Lead*), H Atkins, S Bain, J Dennis, K Ismail, D Johnston, P Kar, K Khunti, C Langenberg, P McArdle, A McGovern, T Peto, J Petrie, E Robertson, N Sattar, K Shah, J Valabhji, B Young

Pulmonary and Systematic Vasculature

L S Howard (*Co-Lead*), Mark Toshner (*Co-Lead*), C Berry, P Chowienczyk, A Lawrie, O C Leavy, J Mitchell, J Newman, L Price, J Quint, A Reddy, J Rossdale, N Sattar, C Sudlow, A A R Thompson, J M Wild, M Wilkins

Rehabilitation, Sarcopenia and Fatigue

S J Singh (*Co-Lead*), W D-C Man (*Co-Lead*), J M Lord (*Co-Lead*), N J Greening (*Co-Lead*), T Chalder (*Co-Lead*), J T Scott (*Co-Lead*), N Armstrong, E Baldry, M Baldwin, N Basu, M Beadsworth, L Bishop, C E Bolton, A Briggs, M Buch, G Carson, J Cavanagh, H Chinoy, C Dawson, E Daynes, S Defres, R A Evans, L Gardiner, P Greenhaff, S Greenwood, M Harvie, L HOuchen-Wolloff, M Husain, S MacDonald, A McArdle, H J C McAuley, A McMahon, M McNarry, G Mills, C Nolan, K O'Donnell, D Parekh, Pimm, J Sargent, L Sigfrid, M Steiner, D Stensel, A L Tan, I Vogiatzis, J Whitney, D Wilkinson, D Wilson, M Witham, D G Wootton, T Yates

Renal

D Thomas (*Lead*), N Brunskill (*Co-Lead*), S Francis (*Co-Lead*), S Greenwood (*Co-Lead*), C Laing (*Co-Lead*), K Bramham, P Chowdhury, A Frankel, L Lightstone, S McAdoo, K McCafferty, M Ostermann, N Selby, C Sharpe, M Willicombe

Patient Public Engagement Group

L Houchen-Wolloff (*Lead*), J Bunker, R Gill, C Hastie, R Nathu, N Rogers, N Smith

Local Clinical Centre PHOSP-COVID trial staff

(listed in alphabetical order)

Airedale NHS Foundation Trust

A Shaw (PI), L Armstrong, B Hairsine, H Henson, C Kurasz, L Shenton

Aneurin Bevan University Health Board

S Fairbairn (PI), A Dell, N Hawkings, J Haworth, M Hoare, A Lucey, V Lewis, G Mallison, H Nassa, C Pennington, A Price, C Price, A Storrie, G Willis, S Young

Barts Health NHS Trust & Queen Mary University of London

P Pfeffer (PI), K Chong-James, C David, W Y James, C Manisty, A Martineau, O Zongo

Barnsley Hospital NHS Foundation Trust

A Sanderson (PI)

Belfast Health and Social Care Trust & Queen's University Belfast

L G Heaney (PI), C Armour, V Brown, T Craig, S Drain, B King, N Magee, D McAulay, E Major, L McGarvey, J McGinness, R Stone

Betsi Cadwaladr University Health Board

A Haggar (PI), A Bolger, F Davies, J Lewis, A Lloyd, R Manley, E McIvor, D Menzies, K Roberts, W Saxon, D Southern, C Subbe, V Whitehead

Borders General Hospital, NHS Borders

H El-Taweel (PI), J Dawson, L Robinson

Bradford Teaching Hospitals NHS Foundation Trust

D Saralaya (PI), L Brear, K Regan, K Storton

Cambridge University Hospitals NHS Foundation Trust, NIHR Cambridge Clinical Research Facility & University of Cambridge

J Fuld (PI), A Bermperi, I Cruz, K Dempsey, A Elmer, H Jones, S Jose, S Marciniak, M Parkes, C Ribeiro, J Taylor, M Toshner, L Watson, J Weir McCall, J Worsley

Cardiff and Vale University Health Board

R Sabit (PI), L Broad, A Buttress, T Evans, M Haynes, L Jones, L Knibbs, A McQueen, C Oliver, K Paradowski, J Williams

Chesterfield Royal Hospital NHS Trust

E Harris (PI), C Sampson

Cwm Taf Morgannwg University Health Board

C Lynch (PI), E Davies, C Evenden , A Hancock, K Hancock, M Rees , L Roche, N Stroud, T Thomas-Woods

East Cheshire NHS Trust

M Babores (PI), J Bradley-Potts, M Holland, N Keenan, S Shashaa, H Wassall

East Kent Hospitals University NHS Foundation Trust

E Beranova (PI), H Weston (PI), T Cosier, L Austin, J Deery, T Hazelton, C Price, H Ramos, R Solly, S Turney

Gateshead NHS Trust

L Pearce (PI), W McCormick, S Pugmire, W Stoker, A Wilson

Guy’s and St Thomas’ NHS Foundation Trust

N Hart (PI), LA Aguilar Jimenez, G Arbane, S Betts, K Bisnauthsing, A Dewar, P Chowdhury, A Chiribiri, A Dewar, G Kaltsakas, H Kerslake, MM Magtoto, P Marino, LM Martinez, C O'Brien, M Ostermann, J Rossdale, TS Solano, E Wynn

Hampshire Hospitals NHS Foundation Trust

N Williams (PI), W Storrar (PI), M Alvarez Corral, A Arias, E Bevan, D Griffin, J Martin, J Owen, S Payne, A Prabhu, A Reed, C Wrey Brown

Harrogate and District NHD Foundation Trust

C Lawson (PI), T Burdett, J Featherstone, A Layton, C Mills, L Stephenson,

Hull University Teaching Hospitals NHS Trust & University of Hull

N Easom (PI), P Atkin, K Brindle, M G Crooks, K Drury, R Flockton, L Holdsworth, A Richards, D L Sykes, S Thackray-Nocera, C Wright

Hywel Dda University Health Board

K E Lewis (PI), A Mohamed (PI), G Ross (PI), S Coetzee, K Davies, R Hughes, R Loosley, L O’Brien, Z Omar, H McGuinness, E Perkins, J Phipps, A Taylor, H Tench, R Wolf-Roberts

Imperial College Healthcare NHS Trust & Imperial College London

L S Howard (PI), O Kon (PI), D C Thomas (PI), S Anifowose, L Burden, E Calvelo, B Card, C Carr, E R Chilvers, D Copeland, P Cullinan, P Daly, C M Efstathiou, L Evison, T Fayzan, H Gordon, S Haq, R G Jenkins, C King, F Liew, K March, M Mariveles, L McLeavey, N Mohamed, S Moriera, U Munawar, J Nunag, U Nwanguma, L Orriss- Dib, D P O'Regan, A Ross, M Roy, E Russell, K Samuel, J Schronce, N Simpson, I D Stewart, L Tarusan, C Wood, N Yasmin

Kettering General Hospital NHS Trust

R Reddy (PI), A-M, Guerdette, M Hewitt, K Warwick, S White

King’s College Hospital NHS Foundation Trust & Kings College London

A M Shah (PI), C J Jolley (PI), O Adeyemi, R Adrego, H Assefa-Kebede, J Breeze, M Brown, S Byrne, T Chalder, A Chiribiri, P Dulawan, N Hart, A Hayday, A Hoare, A Knighton, M Malim, C O'Brien, S Patale, I Peralta, N Powell, A Ramos, K Shevket, F Speranza, A Te

Leeds Teaching Hospitals & University of Leeds

P Beirne (PI), A Ashworth, J Clarke, C Coupland, M Dalton, E Wade, C Favager, J Greenwood, J Glossop, L Hall, T Hardy, A Humphries, J Murira, D Peckham, S Plein, J Rangeley, G Saalmink, A L Tan, B Whittam, N Window, J Woods,

Lewisham & Greenwich NHS Trust

G Coakley (PI)

Liverpool University Hospitals NHS Foundation Trust & University of Liverpool

D G Wootton (PI), L Turtle (PI), L Allerton, AM All, , J Austin, M Beadsworth, A Berridge, J Brown, S Cooper, A Cross, D J Cuthbertson, S Defres, S L Dobson, J Earley, N French, W Greenhalf, H E Hardwick, K Hainey, J Hawkes, V Highett, S Kaprowska, G J Kemp, AL Key, S Koprowska, L Lavelle-Langham, N Lewis-Burke, G Madzamba, F Malein, S Marsh, C Mears, L Melling, M J Noonan, L Poll, J Pratt, E Richardson, A Rowe, M G Semple, V Shaw, K A Tripp, B Vinson, L O Wajero, S A Williams-Howard, J Wyles

London North West University Healthcare NHS Trust

S N Diwanji (PI), P Papineni (PI), S Gurram, S Quaid, G F Tiongson, E Watson

Manchester University NHS Foundation Trust & University of Manchester

B Al-Sheklly (PI), A Horsley (PI), C Avram, P Barran, J Blaikely, M Buch, N Choudhury, D Faluyi, T Felton, T Gorsuch, N A Hanley, T Hussell, Z Kausar, C A Miller, N Odell, R Osbourne, K Piper Hanley, K Radhakrishnan, S Stockdale, D Trivedi

Newcastle upon Tyne Hospitals NHS Foundation Trust & University of Newcastle

A De Soyza (PI), C Echevarria (PI), A Ayoub, J Brown, G Burns, G Davies, H Fisher, C Francis, A Greenhalgh, P Hogarth, J Hughes, K Jiwa, G Jones, G MacGowan, D Price, A Sayer, J Simpson, H Tedd, S Thomas, S West, M Witham, S Wright, A Young

NHS Dumfries and Galloway

M J McMahon (PI), P Neill

NHS Greater Glasgow and Clyde Health Board & University of Glasgow

D Anderson (PI), H Bayes (PI), C Berry (PI), D Grieve (PI), I B McInnes (PI), N Basu, A Brown, A Dougherty, K Fallon, L Gilmour, K Mangion, A Morrow, K Scott, R Sykes, R Touyz

NHS Highland

E K Sage (PI), F Barrett, A Donaldson

NHS Lanarkshire

M Patel (PI), D Bell, A Brown, M Brown, R Hamil, K Leitch, L Macliver, J Quigley, A Smith, B Welsh

NHS Lothian & University of Edinburgh

G Choudhury (PI), J K Baillie, S Clohisey, A Deans, A B Docherty, J Furniss, E M Harrison, S Kelly, N I Lone, D E Newby, A Sheikh

NHS Tayside & University of Dundee

J D Chalmers (PI), D Connell, A Elliott, C Deas, J George, S Mohammed, J Rowland, A R Solstice, D Sutherland, C J Tee

North Bristol NHS Trust & University of Bristol

N Maskell (PI), D Arnold, S Barrett, H Adamali, A Dipper, S Dunn, A Morley, L Morrison, L Stadon, S Waterson, H Welch

North Middlesex Hospital NHS Trust

B Jayaraman (PI), T Light

Nottingham University Hospitals NHS Trust & University of Nottingham

C E Bolton (PI), P Almeida, J Bonnington, M Chrystal, E Cox, C Dupont, S Francis, P Greenhaff, A Gupta, L Howard, W Jang, S Linford, L Matthews, R Needham, A Nikolaidis, S Prosper, K Shaw, A K Thomas

Oxford University Hospitals NHS Foundation Trust & University of Oxford

L P Ho (PI), N M Rahman (PI), M Ainsworth, A Alamoudi, M Beggs, A Bates, A Bloss, A Burns, P Carter, M Cassar, K M Channon, J Chen, F Conneh, T Dong, R I Evans, E Fraser, X Fu, J R Geddes, F Gleeson, P Harrison, M Havinden-Williams, P Jezzard, N Kanellakis, I Koychev, P Kurupati, X Li, E Lukaschuk, K McGlynn, H McShane, C Megson, K Motohashi, S Neubauer, D Nicoll, G Ogg, E Pacpaco, M Pavlides, Y Peng, N Petousi, J Propescu, N Rahman, B Raman, M J Rowland, K Saunders, M Sharpe, N Talbot, E Tunnicliffe

Royal Brompton and Harefield Clinical Group, Guy’s and St Thomas’ NHS Foundation Trust.

W D-C Man (PI), B Patel (PI), R E Barker, D Cristiano, N Dormand, M Gummadi, S Kon, K Liyanage, C M Nolan, S Patel, O Polgar, P Shah, S J Singh, J A Walsh

Royal Free London NHS Foundation Trust

J Hurst (PI), H Jarvis (PI), S Mandal (PI), S Ahmad, S Brill, L Lim, D Matila, O Olaosebikan, C Singh

Royal Papworth Hospital NHS Foundation Trust

M Toshner (PI), H Baxendale, L Garner, C Johnson, J Mackie, A Michael, J Pack, K Paques, H Parfrey, J Parmar

Salford Royal NHS Foundation Trust

N Diar Bakerly (PI), P Dark, D Evans, E Hardy, A Harvey, D Holgate, S Knight, N Mairs, N Majeed, L McMorrow, J Oxton, J Pendlebury, C Summersgill, R Ugwuoke, S Whittaker

Salisbury NHS Foundation Trust

W Matimba-Mupaya (PI), S Strong-Sheldrake

Sheffield Teaching NHS Foundation Trust & University of Sheffield

S L Rowland-Jones (PI), A A R Thompson (Co PI), J Bagshaw, M Begum, K Birchall, R Butcher, H Carborn, F Chan, K Chapman, Y Cheng, L Chetham, C Clark, Z Coburn, J Cole, M Dixon, A Fairman, J Finnigan, L Finnigan, H Foot, D Foote, A Ford, R Gregory, K Harrington, L Haslam, L Hesselden, J Hockridge, A Holbourn, B Holroyd-Hind, L Holt, A Howell, E Hurditch, F Ilyas, C Jarman, A Lawrie, E Lee, J-H Lee, R Lenagh, A Lye, I Macharia, M Marshall, A Mbuyisa, J McNeill, S Megson, J Meiring, L Milner, S Misra, H Newell, T Newman, C Norman, L Nwafor, D Pattenadk, M Plowright, J Porter, P Ravencroft, C Roddis, J Rodger, P Saunders, J Sidebottom, J Smith, L Smith, N Steele, G Stephens, R Stimpson, B Thamu, N Tinker, K Turner, H Turton, P Wade, S Walker, J Watson, J M Wild, I Wilson, A Zawia

St George's University Hospitals NHS Foundation Trust

R Aul (PI), M Ali, A Dunleavy (PI), D Forton, N Msimanga, M Mencias, T Samakomva, S Siddique, J Teixeira, V Tavoukjian

Sherwood Forest Hospitals NHS Foundation Trust

J Hutchinson (PI), L Allsop, K Bennett, P Buckley, M Flynn, M Gill, C Goodwin, M Greatorex, H Gregory, C Heeley, L Holloway, M Holmes, J Kirk, W Lovegrove, TA Sewell, S Shelton, D Sissons, K Slack, S Smith, D Sowter, S Turner, V Whitworth, I Wynter

Shropshire Community Health NHS Trust

L Warburton (PI), S Painter, J Tomlinson

Somerset NHS Foundation Trust

C Vickers (PI), T Wainwright, D Redwood, J Tilley, S Palmer

Swansea Bay University Health Board

G A Davies (PI), L Connor, A Cook, T Rees, F Thaivalappil, C Thomas

Tameside and Glossop Integrated Care NHS Foundation

A Butt (PI), M Coulding, H Jones, S Kilroy, J McCormick, J McIntosh, H Savill, V Turner, J Vere

The Great Western Hospital Foundation Trust

E Fraile (PI), J Ugoji

The Hillingdon Hospitals NHS Foundation Trust

S S Kon (PI), H Lota, G Landers, M Nasseri, S Portukhay

The Rotherham NHS Foundation Trust

A Hormis (PI), A Daniels, J Ingham, L Zeidan

United Lincolnshire Hospitals NHS Trust

M Chablani (PI), L Osborne

University College London Hospital & University College London

M Marks (PI), J S Brown (PI), N Ahwireng, B Bang, D Basire, R C Chambers, A Checkley, R Evans, M Heightman, T Hillman, J Hurst, J Jacob, S Janes, R Jastrub, M Lipman, S Logan, D Lomas, M Merida Morillas, A Pakzad, H Plant, J C Porter, K Roy, E Wall, B Williams, M Xu

University Hospital Birmingham NHS Foundation Trust & University of Birmingham

D Parekh (PI), N Ahmad Haider, C Atkin, R Baggott, M Bates, A Botkai, A Casey, B Cooper, J Dasgin, K Draxlbauer, N Gautam, J Hazeldine, T Hiwot, S Holden, K Isaacs, T Jackson, S Johnson, V Kamwa, D Lewis, J M Lord, S Madathil, C McGhee, K Mcgee, A Neal, A Newton Cox, J Nyaboko, D Parekh, Z Peterkin, H Qureshi, B Rangelov, L Ratcliffe, E Sapey, J Short, T Soulsby, R Steeds, J Stockley, Z Suleiman, T Thompson, M Ventura, S Walder, C Welch, D Wilson, S Yasmin, K P Yip

University Hospitals of Derby and Burton

P Beckett (PI) C Dickens, U Nanda

University Hospitals of Leicester NHS Trust & University of Leicester

C E Brightling (CI), R A Evans (PI), M Aljaroof, N Armstrong, H Arnold, H Aung, M Bakali, M Bakau, M Baldwin, M Bingham, M Bourne, C Bourne, N Brunskill, P Cairns, L Carr, A Charalambou, C Christie, M J Davies, S Diver, S Edwards, C Edwardson, O Elneima, H Evans, J Finch, S Glover, N Goodman, B Gootpu, N J Greening, B Guillen-Guio, K Hadley, P Haldar, B Hargadon, V C Harris, L Houchen-Wolloff, W Ibrahim, L Ingram, K Khunti, A Lea, D Lee, D Lozano-Rojas, G P McCann, H J C McAuley, P McCourt, T Mcnally, G Mills, A Moss, W Monteiro, K Ntotsis, M Pareek, S Parker, A Rowland, A Prickett, I N Qureshi, R Russell, N Samani, M Sereno, M Sharma, A Shikotra, S Siddiqui, A Singapuri, S J Singh, J Skeemer, M Soares, E Stringer, T Thornton, M Tobin, E Turner, L V Wain, T J C Ward, F Woodhead, J Wormleighton, T Yates, A Yousuf,

University Hospital Southampton NHS Foundation Trust & University of Southampton

M G Jones (PI), C Childs, R Djukanovic, S Fletcher, M Harvey, E Marouzet, B Marshall, R Samuel, T Sass, T Wallis, H Wheeler

Whittington Health NHS

R Dharmagunawardena (PI), E Bright, P Crisp, M Stern

Wirral University Teaching Hospital

A Wight (PI), L Bailey, A Reddington

Wrightington Wigan and Leigh NHS trust

A Ashish (PI), J Cooper, E Robinson

Yeovil District Hospital NHS Foundation Trust

A Broadley (PI)

York & Scarborough NHS Foundation Trust

K Howard (PI), L Barman, C Brookes, K Elliott. L Griffiths, Z Guy, D Ionita, H Redfearn, C Sarginson, A Turnbull

Health and Care Research Wales

Y Ellis

London School of Hygiene & Tropical Medicine (LSHTM)

M Marks, A Briggs

NIHR Office for Clinical Research Infrastructure

K Holmes

Patient Public Involvement Leads

Asthma UK and British Lung Foundation Partnership - K Poinasamy, S Walker

Royal Surrey NHS Foundation Trust

M Halling-Brown

South London and Maudsley NHS Foundation Trust & Kings College London

G Breen, M Hotopf

Swansea University & Swansea Welsh Network

K Lewis, N Williams

Supplementary methods

DNA extraction and sequencing

0.1g of sputum or pelleted 2ml of oral wash were homogenised with lysis buffer by bead-beating for three minutes using the TissueLyser LT (Qiagen), followed by centrifugation. The lysate was processed utilising the QIAcube connect according to manufacturers' instructions. Samples were processed in batches alongside 100µl of molecular grade water as DNA extraction controls. For every ten oral rinses from a participating site, one oral rinse negative control was DNA extracted as an environmental control. Two participating sites had all oral wash negative controls extracted to verify suitability of random negative sampling. DNA was quantified using Qubit dsDNA broad range kit (Thermo Fisher Cat No. Q32853) and Qubit dsDNA HS kit (Thermo Fisher Cat No. Q33231).

Sequencing of the V3-V4 region of 16S ribosomal RNA (rRNA) was performed on an Illumina MiSeq platform utilising a 600 cycle V3 MiSeq cartridge (Illumina), following the Illumina protocol: https://support.illumina.com/content/dam/illumina-support/documents/documentation/chemistry_documentation/16s/16smetagenomic-library-prep-guide-15044223-b.pdf with some modifications. Four forward and four reverse primers with variable length poly N regions were used to reduce amplification bias (Table S13). 30 cycles were used for each PCR due to the low biomass of airway samples. Sputum samples and oral rinse samples were processed in separate sequencing runs. For each sequencing run PCR (molecular water) and QEB negative controls, plus a positive control (ZymoBIOMICS Microbial Community DNA Standard-D6305) were included.

Cutadapt 3.1 with Python 3.8.6 was used to remove Illumina adapters from the pair-end raw sequencing reads (1). Quality of reads was checked using FASTQC in R 4.1.3 (2022-03-10) (2). ASVs were generated through DADA2 version 1.22.0 with taxonomy assignment using Silva v.138.1 database.

Potential contaminants were determined using 'decontam' package (version 1.13) in R 4.1.3 (2022-03-10) using the prevalence method (0.10 threshold)(3). Potential contaminant ASVs were sense checked prior removal from dataset (Supplementary tables S2-3). Taxa unclassified to phylum level, Mitochondria, Erysipelothrix and Chloroplast were removed prior to analysis. A minimum number of reads threshold was set based on the maximum read counts of negative controls per sequencing run, this removed one sample from the analysis. Taxa were agglomerated at genus level.

Microbiota analysis

Multiple imputation R package 'mice' (version 3.16.0) imputed missing data for linear regression models. A pre-processing step removed taxa present in singletons and in <1% of samples prior to beta diversity assessment.

Random Forest was performed using the 'randomForest' R package (version 4.7-1.1) with 500 trees for the top 20 differential taxa (error rates reported in Table S14). MicrobiotaProcess diff_analysis function R package (version 1.16.1) as default with FDR correction, the threshold was set to adjusted p-value <0.05 (4). DESeq2 was performed using geometric mean of positive counts and Wald test with Benjamini-Hochberg (BH) correction, the threshold was set to adjusted p-value <0.05.

Co-occurrence network

To reduce co-occurrence network noise a taxa filter was applied. Sputum microbiota analysis included only taxa abundant ≥0.1% in at least 5% of samples. To account for greater diversity, oral microbiota analysis included only taxa abundant ≥0.1% in at least 10% of samples. Networks were generated using the same number of samples for each group (i.e. the sample size of the smallest group) to ensure comparability. To ensure network robustness, the Stability Approach to Regularization Selection (StARS) was applied as part of SPIEC-EASI using 50 subsampling replicates with a 0.8 subsample ratio and a threshold of 0.05, corresponding to edges that are highly stable across subsamples (present in approximately 95% of resampled networks). Hubs were identified using all centrality measurements (betweenness, closeness, eigenvector, degree) with a hub threshold set at the 85th percentile, using NetCoMi function netAnalyze. Network comparisons were performed for largest connected component and whole network using NetCoMi function netCompare. Jaccard index was used for centrality measurements comparisons, adjusted rand index was used for clustering similarity comparisons. Permutation test with Benjamini-Hochberg adjustment and 1000 permutations was applied for all network comparisons.

Inflammatory protein assay and over representation analysis

To investigate the relationship between systemic inflammation and oral/sputum microbiota, Spearmans' rank correlation analysis was performed using microbiota taxa relative abundance and protein levels (NPX) utilising

Hmisc R package (version 5.1-3). Only co-occurrence network hub taxa were selected for this analysis. A threshold of 0.4 for correlation coefficient and <0.05 for adjusted p-value by False Discovery Rate (FDR) correction was set. Heatmaps were prepared using ggplot2 package in R (version 3.1.3).

Over representation analysis was performed utilising the genekitr R package (version 1.2.8). To account for the proteins tested, the Olink Explore 384 Inflammation panel was used as the background for the over representation analysis. A minimum count of three was applied, with a threshold of <0.01 FDR adjusted p-value.

General approach to multiple testing

A pragmatic approach to adjustment for multiple testing has been taken throughout, in line with the ideas described by Boulesteix and Hoffmann, and Rubin (2021); among others (5, 6).

No adjustment has been performed where a relatively small number of pre-planned comparisons have been performed, and reporting is not selective based on the results obtained. This is the case for the comparisons of alpha diversity by severity cluster (with analysis repeated on other alpha diversity measures as sensitivity analyses) and by LC symptom subgroup.

Adjustment has been performed, for example, for differential abundance comparing multiple features between groups, and where the planned reporting and interpretation of results would focus on the biggest differences.

Supplementary figures

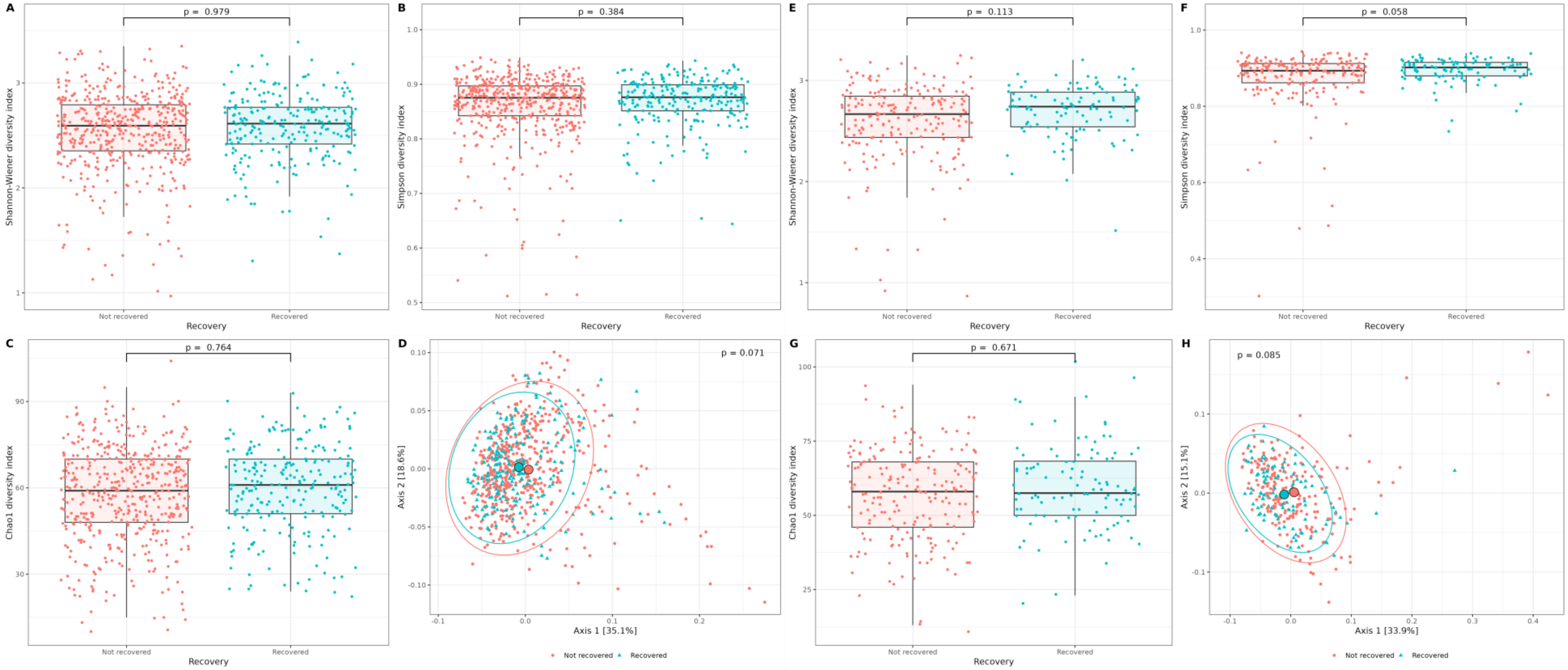

Figure S1 Shannon-Wiener diversity index (alpha diversity) based on participant recovery post-COVID-19 hospitalisation for **(A)** oral and **(E)** sputum microbiota. Simpson diversity index (alpha diversity) based on participant recovery post-COVID-19 hospitalisation for **(B)** oral and **(F)** sputum microbiota. Chao1 diversity index (alpha diversity) based on participant recovery post-COVID-19 hospitalisation for **(C)** oral and **(G)** sputum microbiota. Weighted UniFrac principal coordinates analysis plot comparing recovered (blue triangles) and non-recovered (red circles) participants for **(D)** oral and **(H)** sputum microbiota. Large bold circle represents centroid of group.

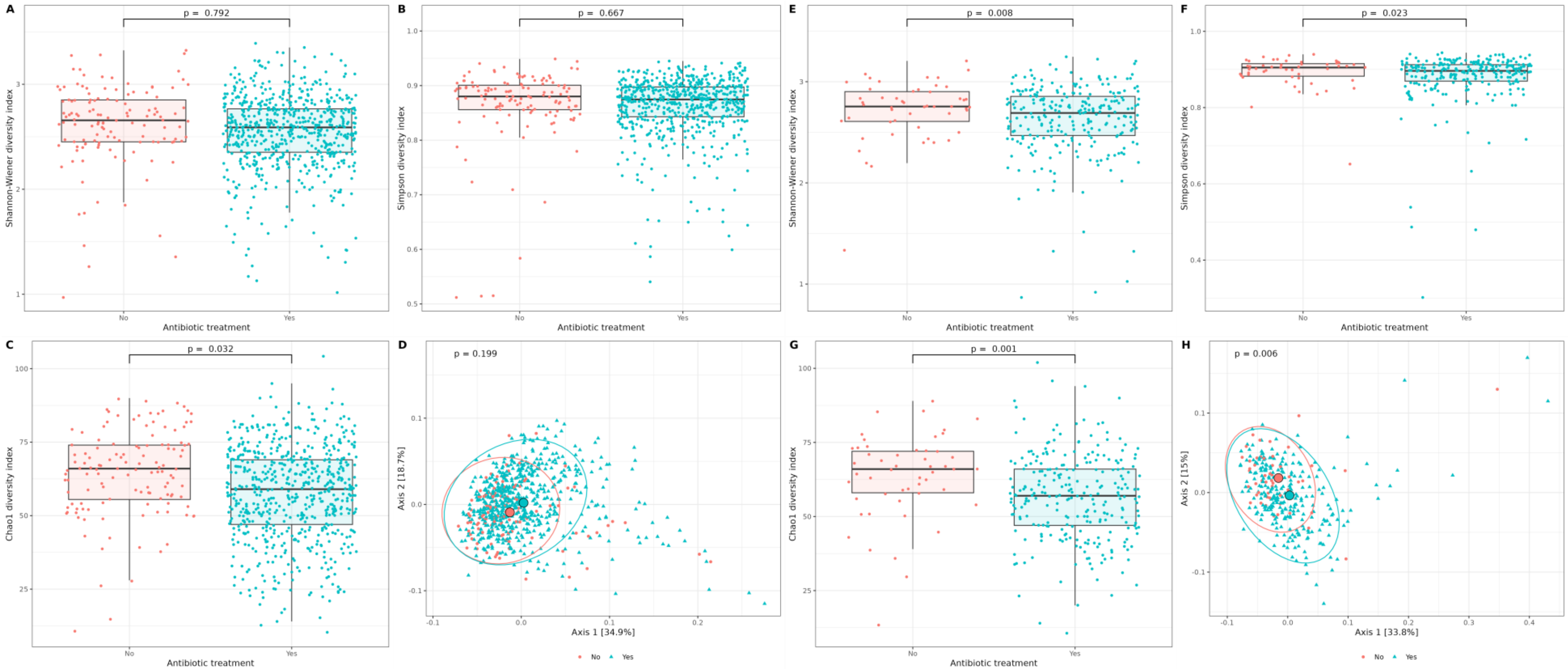

Figure S2 Shannon-Wiener diversity index (alpha diversity) based on antibiotic treatment during COVID-19 hospitalisation for **(A)** oral and **(E)** sputum microbiota. Simpson diversity index (alpha diversity) based on antibiotic treatment during COVID-19 hospitalisation for **(B)** oral and **(F)** sputum microbiota. Chao1 diversity index (alpha diversity) based on antibiotic treatment during COVID-19 hospitalisation for **(C)** oral and **(G)** sputum microbiota. Weighted UniFrac principal coordinates analysis plot comparing receiving antibiotic treatment (blue triangles) and not (red circles) for **(D)** oral and **(H)** sputum microbiota. Large bold circle represents centroid of group.

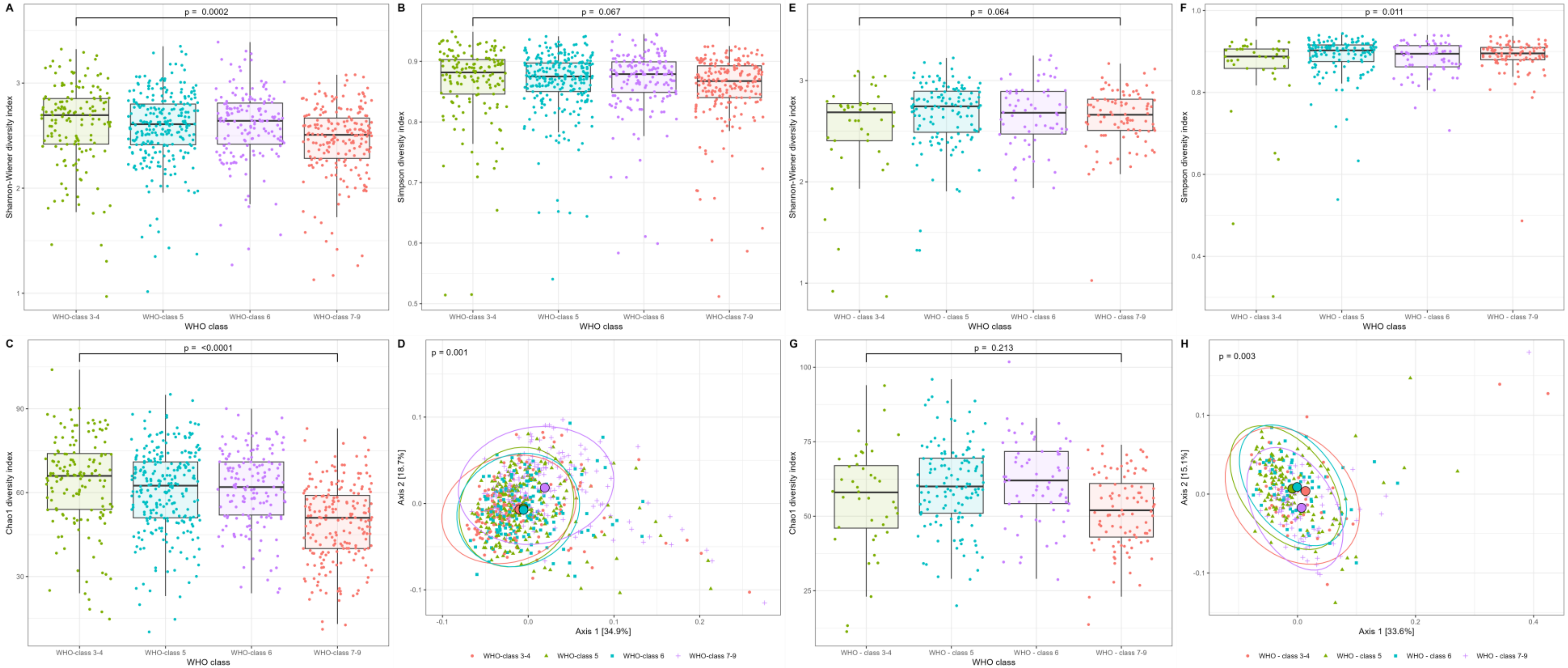

Figure S3 Shannon-Wiener diversity index (alpha diversity) based on participant WHO class at hospitalisation for **(A)** oral and **(E)** sputum microbiota. Simpson diversity index (alpha diversity) based on participant WHO class at hospitalisation for **(B)** oral and **(F)** sputum microbiota. Chao1 diversity index (alpha diversity) based on participant WHO class at hospitalisation for **(C)** oral and **(G)** sputum microbiota. Weighted UniFrac principal coordinates analysis plot comparing participant WHO class at hospitalisation for **(D)** oral and **(H)** sputum microbiota. Large bold circle represents centroid of group.

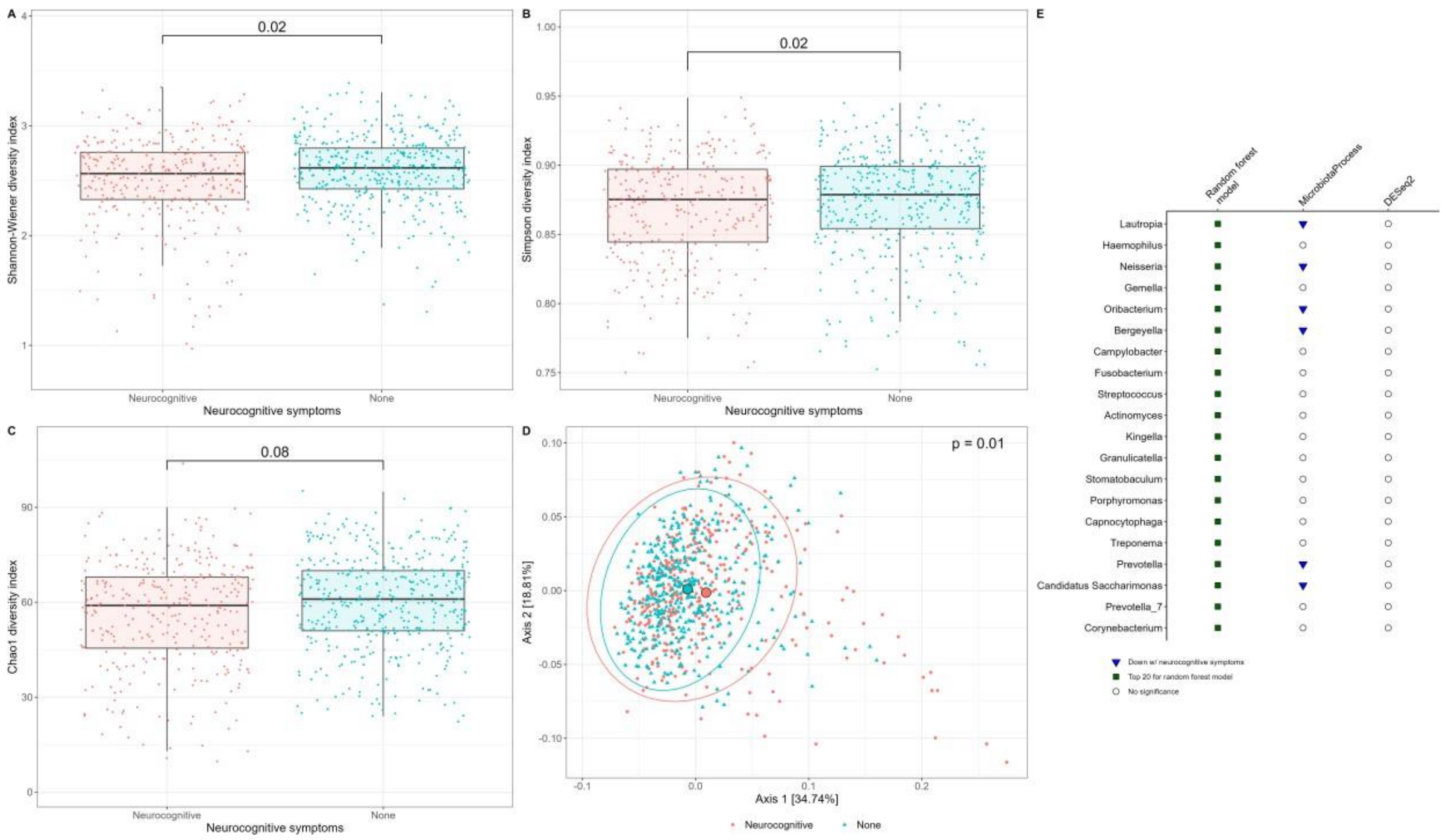

Figure S4 Comparison of oral microbiota for participants with and without neurocognitive symptoms. **(A)** Shannon-Wiener diversity index (alpha diversity). **(B)** Simpson diversity index (alpha diversity). **(C)** Chao1 diversity index (alpha diversity). **(D)** Weighted UniFrac principal coordinates analysis plot. Large bold circle represents centroid of group. **(E)** differential abundance analysis summary of three methods: random forest model, MicrobiotaProcess and DESeq2.

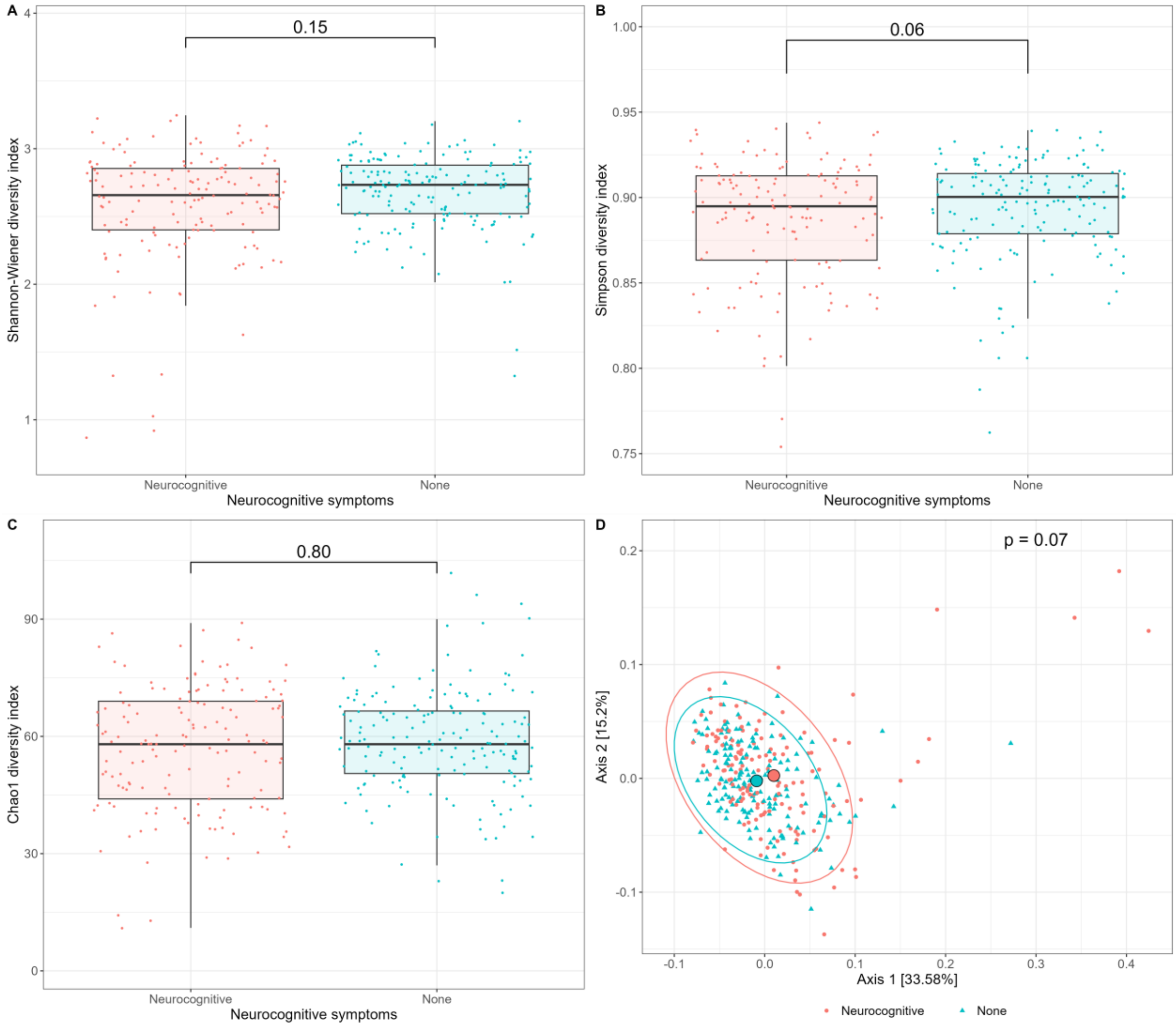

Figure S5 Comparison of sputum microbiota for participants with and without neurocognitive symptoms. **(A)** Shannon-Wiener diversity index (alpha diversity). **(B)** Simpson diversity index (alpha diversity). **(C)** Chao1 diversity index (alpha diversity). **(D)** Weighted UniFrac principal coordinates analysis plot. Large bold circle represents centroid of group.

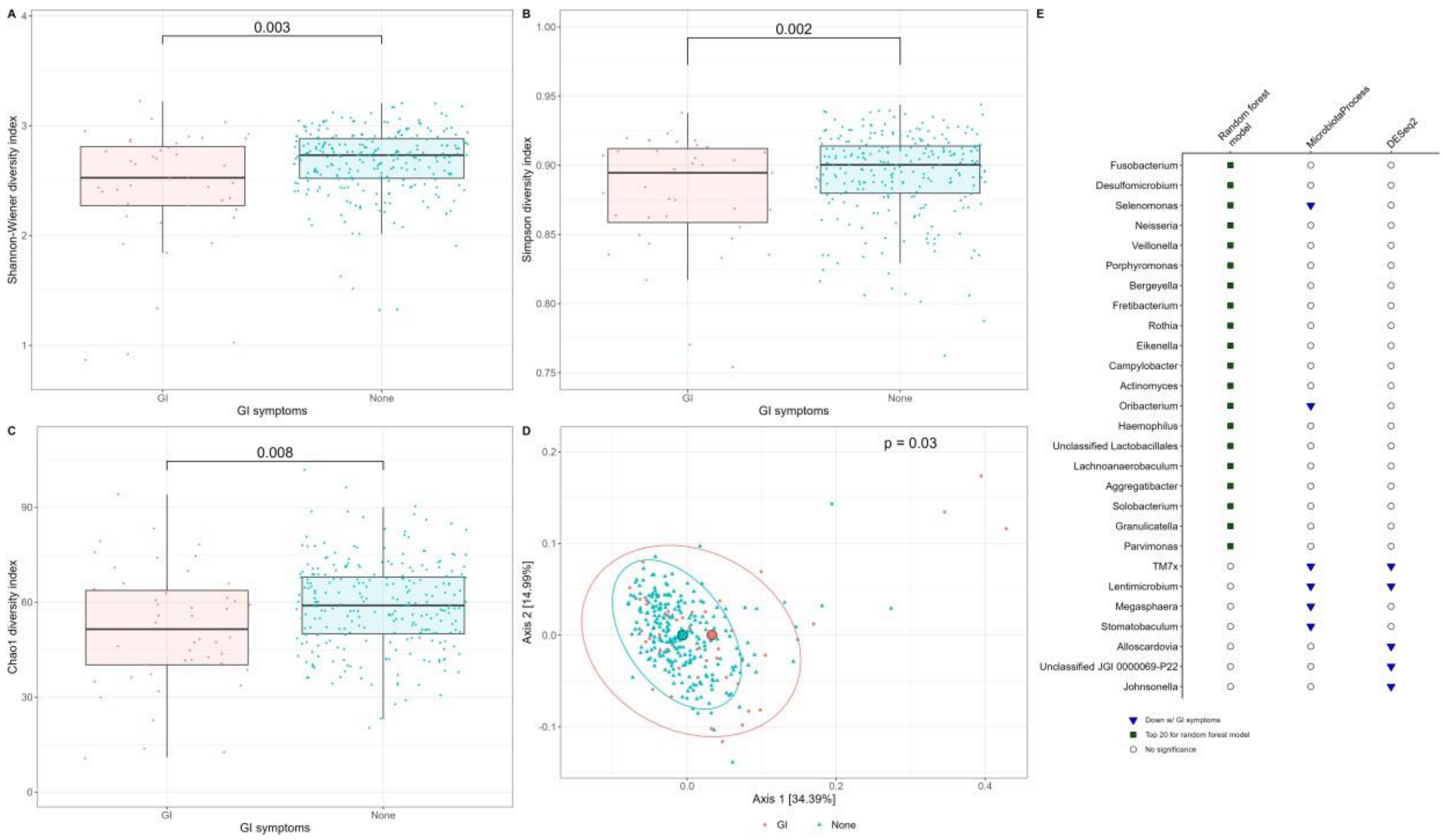

Figure S6 Comparison of sputum microbiota for participants with and without gastrointestinal symptoms. **(A)** Shannon-Wiener diversity index (alpha diversity). **(B)** Simpson diversity index (alpha diversity). **(C)** Chao1 diversity index (alpha diversity). **(D)** Weighted UniFrac principal coordinates analysis plot. Large bold circle represents centroid of group. **(E)** differential abundance analysis summary of three methods: random forest model, MicrobiotaProcess and DESeq2.

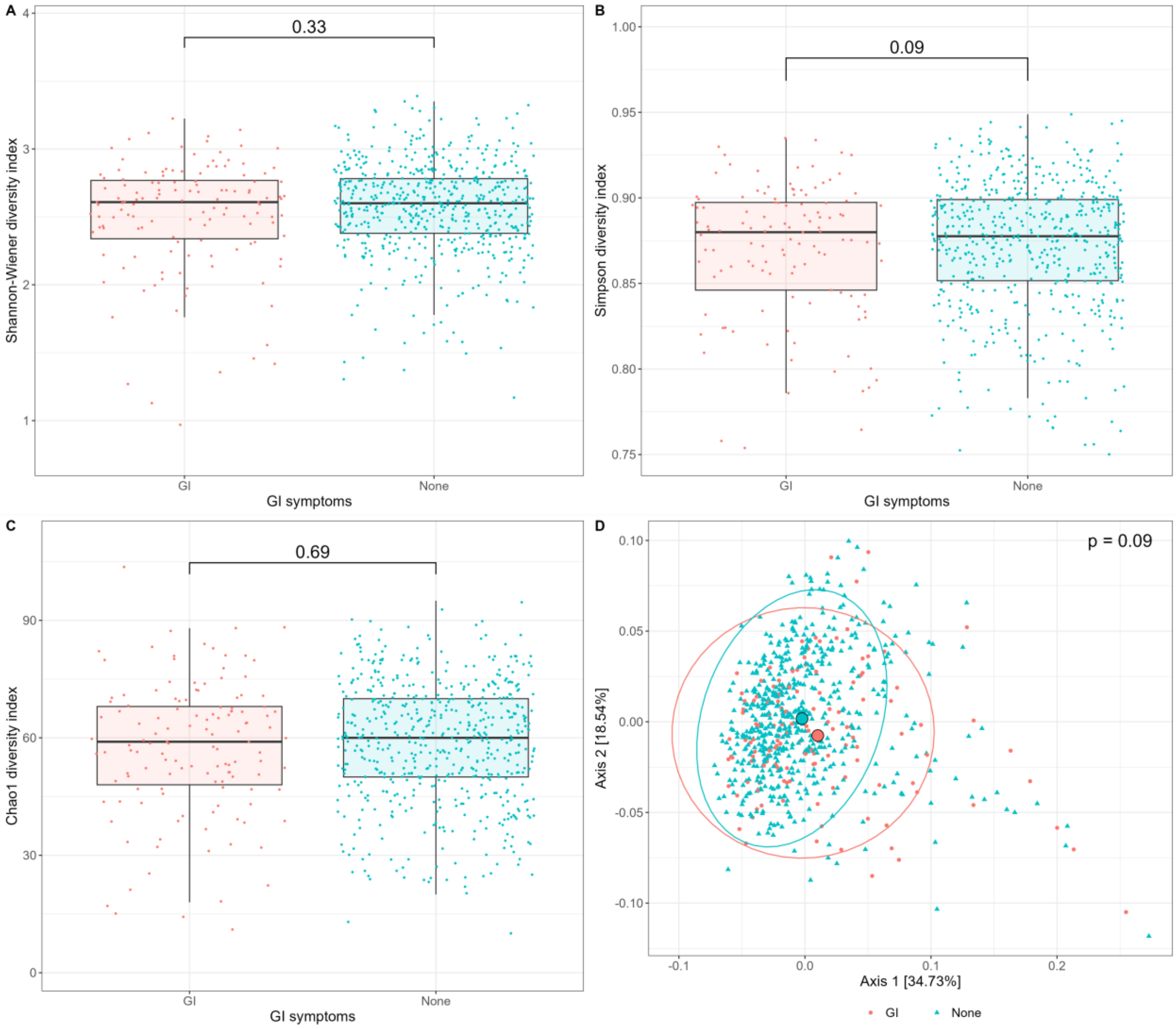

Figure S7 Comparison of oral microbiota for participants with and without gastrointestinal symptoms. **(A)** Shannon-Wiener diversity index (alpha diversity). **(B)** Simpson diversity index (alpha diversity). **(C)** Chao1 diversity index (alpha diversity). **(D)** Weighted UniFrac principal coordinates analysis plot. Large bold circle represents centroid of group.

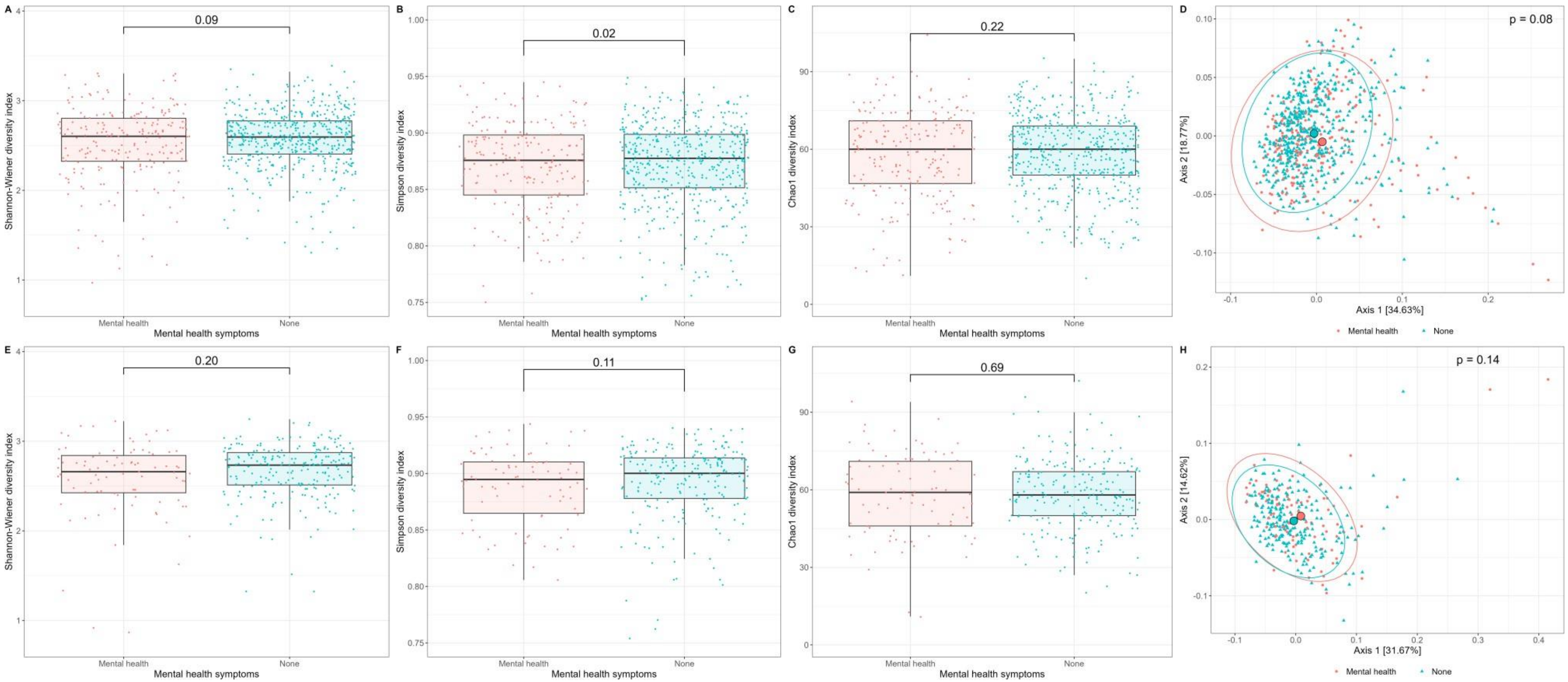

Figure S8 Comparison of microbiota for participants with and without mental health symptoms. **(A)** Shannon-Wiener diversity index (alpha diversity) for oral microbiota. **(B)** Simpson diversity index (alpha diversity) for oral microbiota. **(C)** Chao1 diversity index (alpha diversity) for oral microbiota. **(D)** Weighted UniFrac principal coordinates analysis plot for oral microbiota. Large bold circle represents centroid of group. **(E)** Shannon-Wiener diversity index (alpha diversity) for sputum microbiota. **(F)** Simpson diversity index (alpha diversity) for sputum microbiota. **(G)** Chao1 diversity index (alpha diversity) for sputum microbiota. **(H)** Weighted UniFrac principal coordinates analysis plot for sputum microbiota. Large bold circle represents centroid of group.

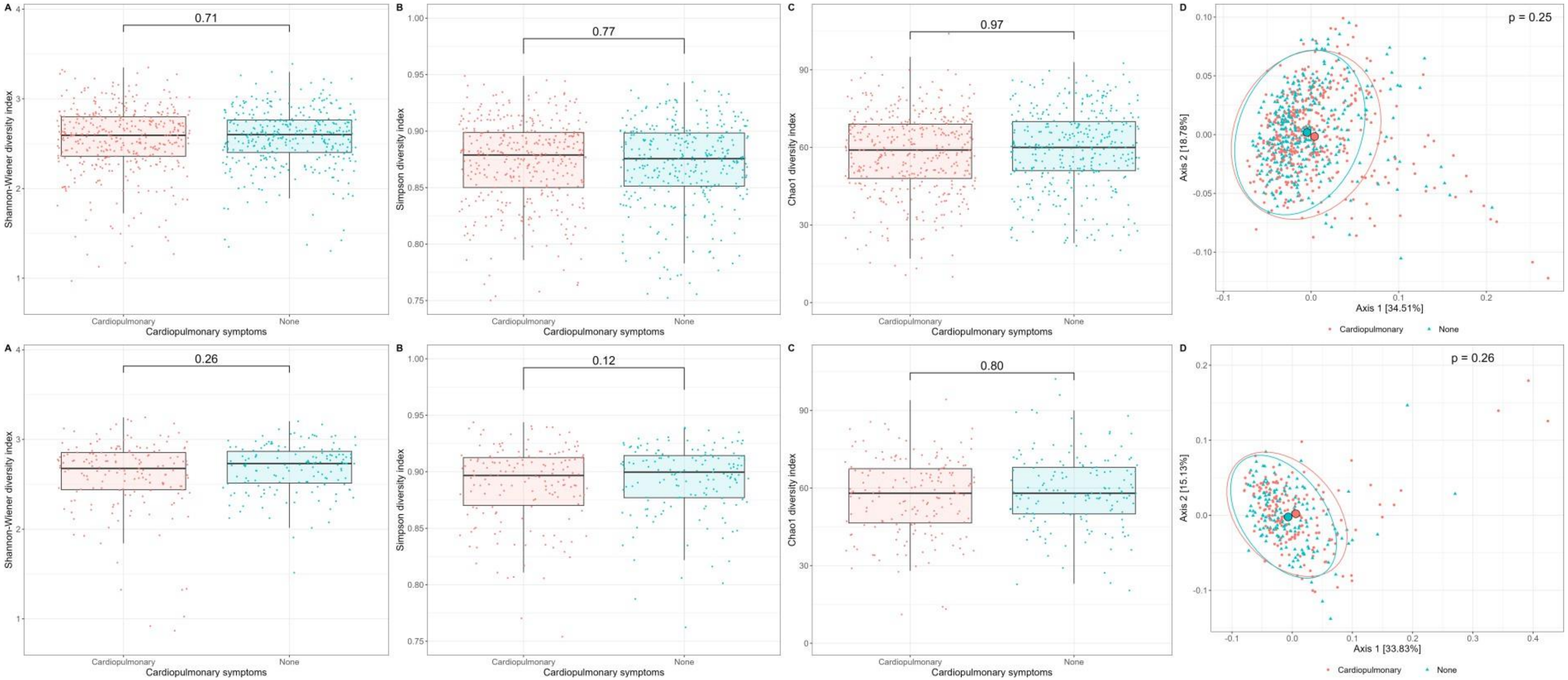

Figure S9 Comparison of microbiota for participants with and without cardiopulmonary symptoms. **(A)** Shannon-Wiener diversity index (alpha diversity) for oral microbiota. **(B)** Simpson diversity index (alpha diversity) for oral microbiota. **(C)** Chao1 diversity index (alpha diversity) for oral microbiota. **(D)** Weighted UniFrac principal coordinates analysis plot for oral microbiota. Large bold circle represents centroid of group. **(E)** Shannon-Wiener diversity index (alpha diversity) for sputum microbiota. **(F)** Simpson diversity index (alpha diversity) for sputum microbiota. **(G)** Chao1 diversity index (alpha diversity) for sputum microbiota. **(H)** Weighted UniFrac principal coordinates analysis plot for sputum microbiota. Large bold circle represents centroid of group.

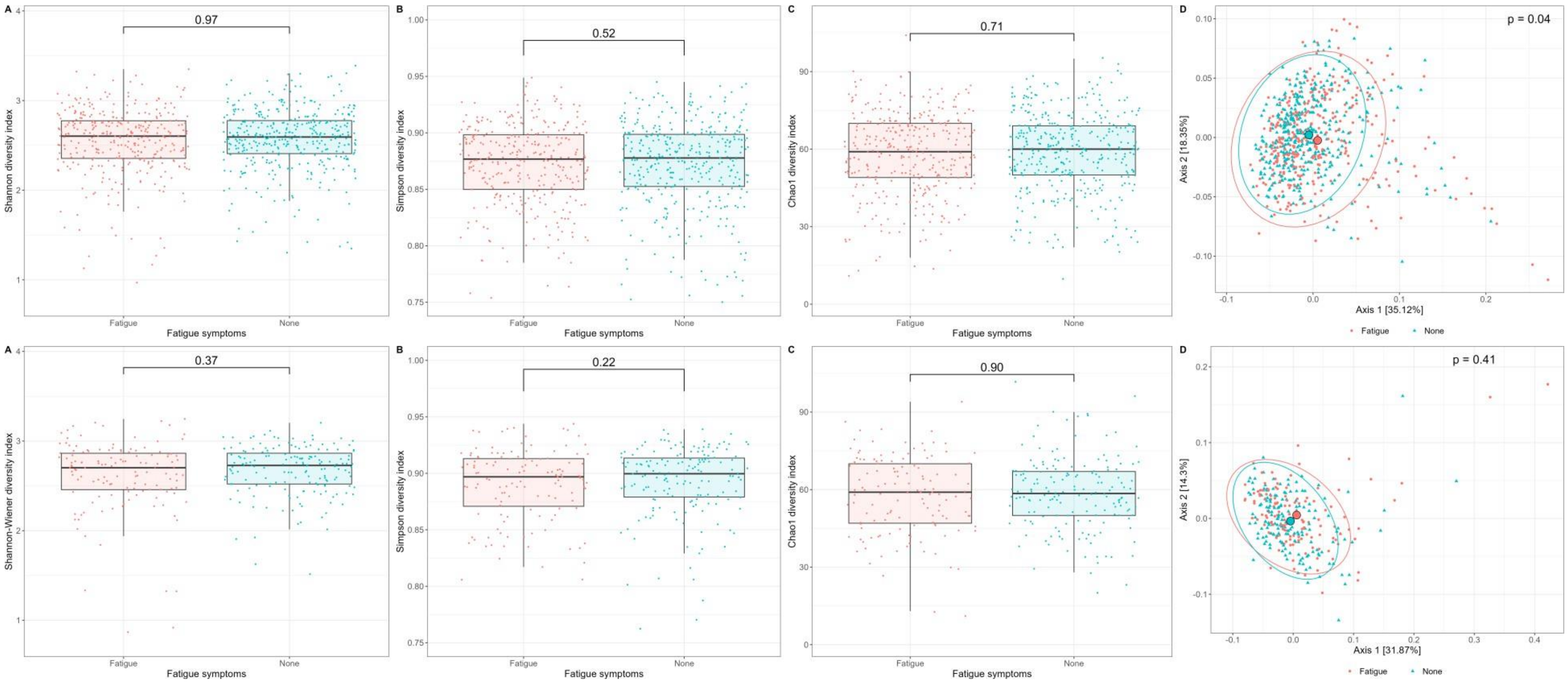

Figure S10 Comparison of microbiota for participants with and without fatigue symptoms. **(A)** Shannon-Wiener diversity index (alpha diversity) for oral microbiota. **(B)** Simpson diversity index (alpha diversity) for oral microbiota. **(C)** Chao1 diversity index (alpha diversity) for oral microbiota. **(D)** Weighted UniFrac principal coordinates analysis plot for oral microbiota. Large bold circle represents centroid of group. **(E)** Shannon-Wiener diversity index (alpha diversity) for sputum microbiota. **(F)** Simpson diversity index (alpha diversity) for sputum microbiota. **(G)** Chao1 diversity index (alpha diversity) for sputum microbiota. **(H)** Weighted UniFrac principal coordinates analysis plot for sputum microbiota. Large bold circle represents centroid of group.

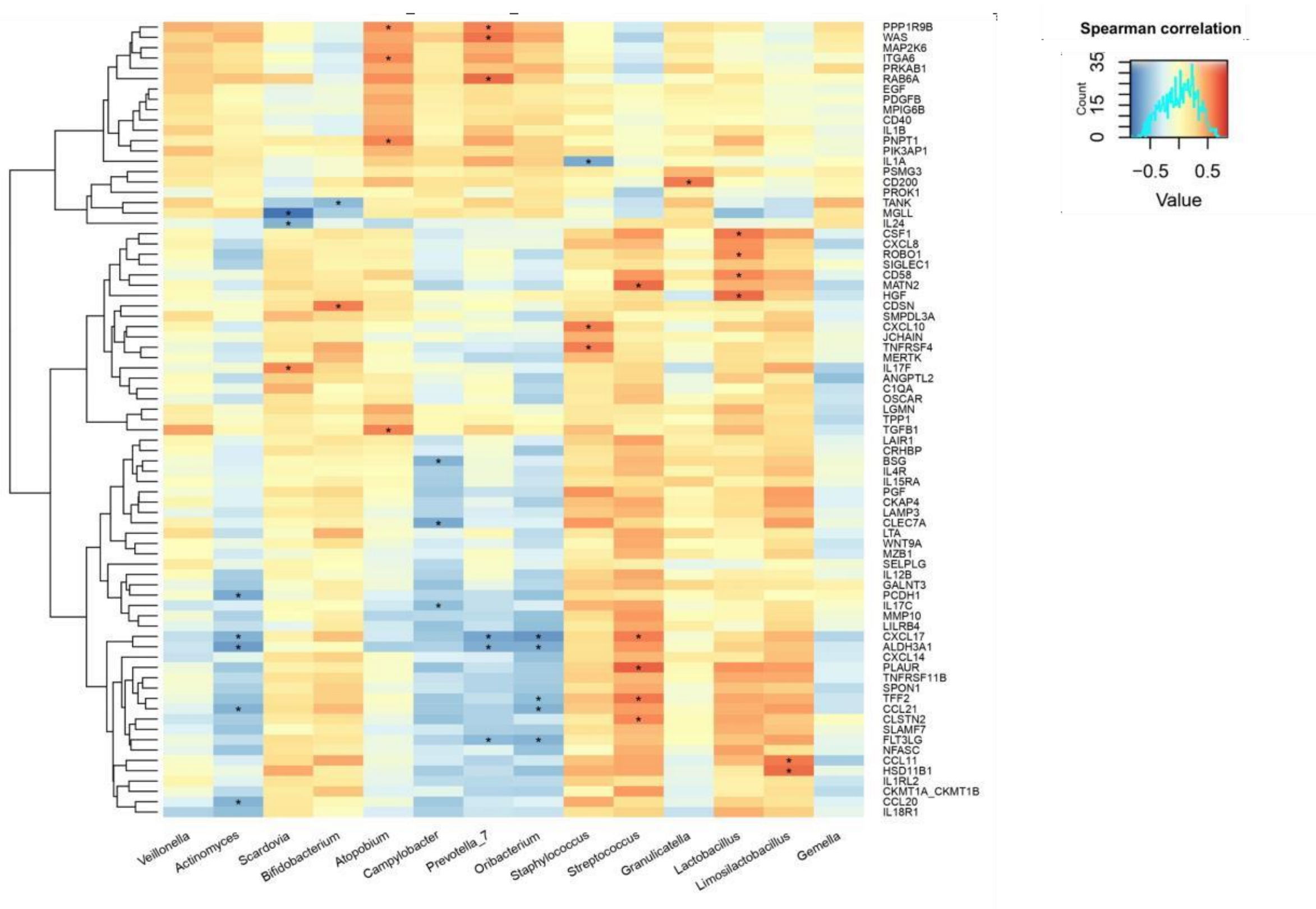

Figure S11 Sputum microbiota in patients with GI symptoms correlation with inflammation related proteins. A spearman rank correlation analysis for the co-occurrence network hubs with inflammation related proteins levels (NPX), from O-link Explore 384 Inflammation panel. * p-value < 0.05 (FDR adjusted) and absolute $r^2 \geq 0.4$.

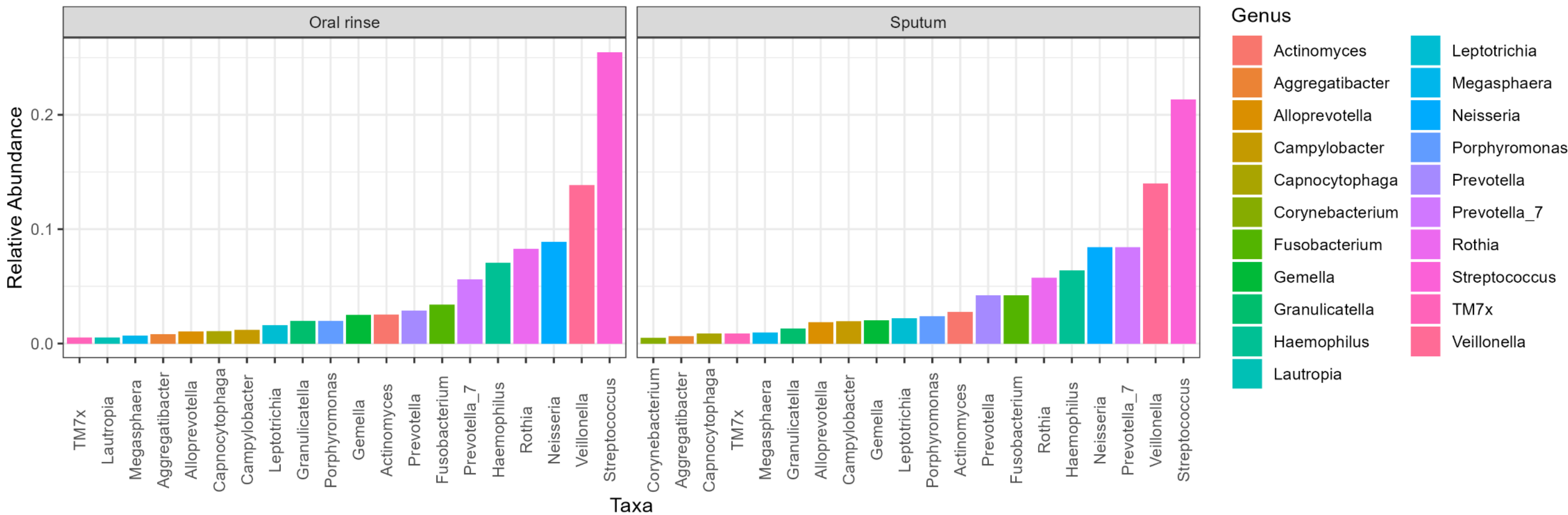

Figure S12 Relative abundance of the top 20 taxa for sputum and oral rinse samples.

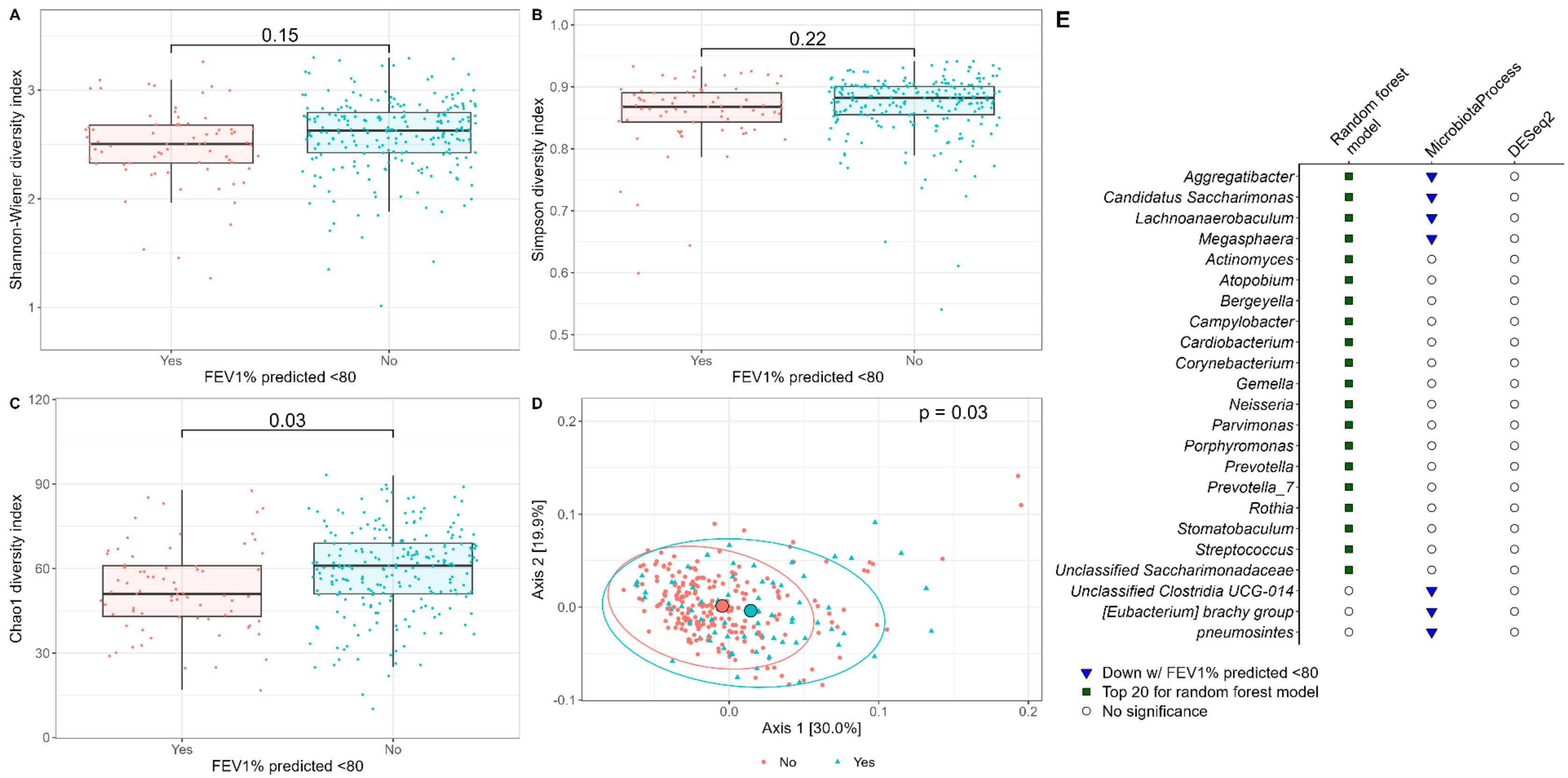

Figure S13 Comparison of oral microbiota for participants with FEV1 <80% and >80 % predicted. **(A)** Shannon-Wiener diversity index (alpha diversity). **(B)** Simpson diversity index (alpha diversity). **(C)** Chao1 diversity index. **(D)** Weighted UniFrac principal coordinates analysis plot. Large bold circle represents centroid of group. **(E)** Differential abundance analysis summary of three methods: random forest model, MicrobiotaProcess and DESeq2.

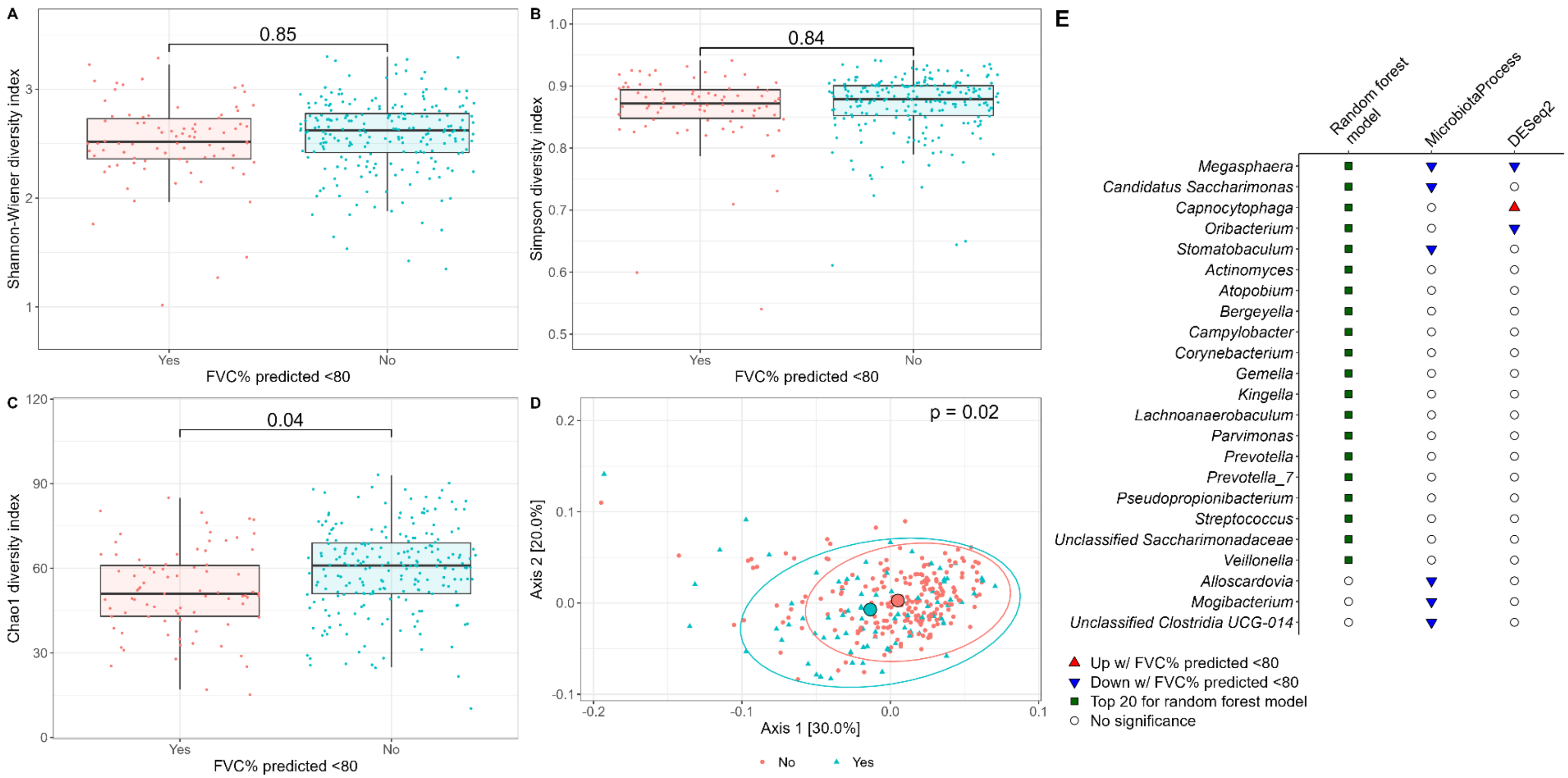

Figure S14 Comparison of oral microbiota for participants with FVC <80% and >80% predicted. **(A)** Shannon-Wiener diversity index (alpha diversity). **(B)** Simpson diversity index (alpha diversity). **(C)** Chao1 diversity index. **(D)** Weighted UniFrac principal coordinates analysis plot. Large bold circle represents centroid of group. **(E)** Differential abundance analysis summary of three methods: random forest model, MicrobiotaProcess and DESeq2.

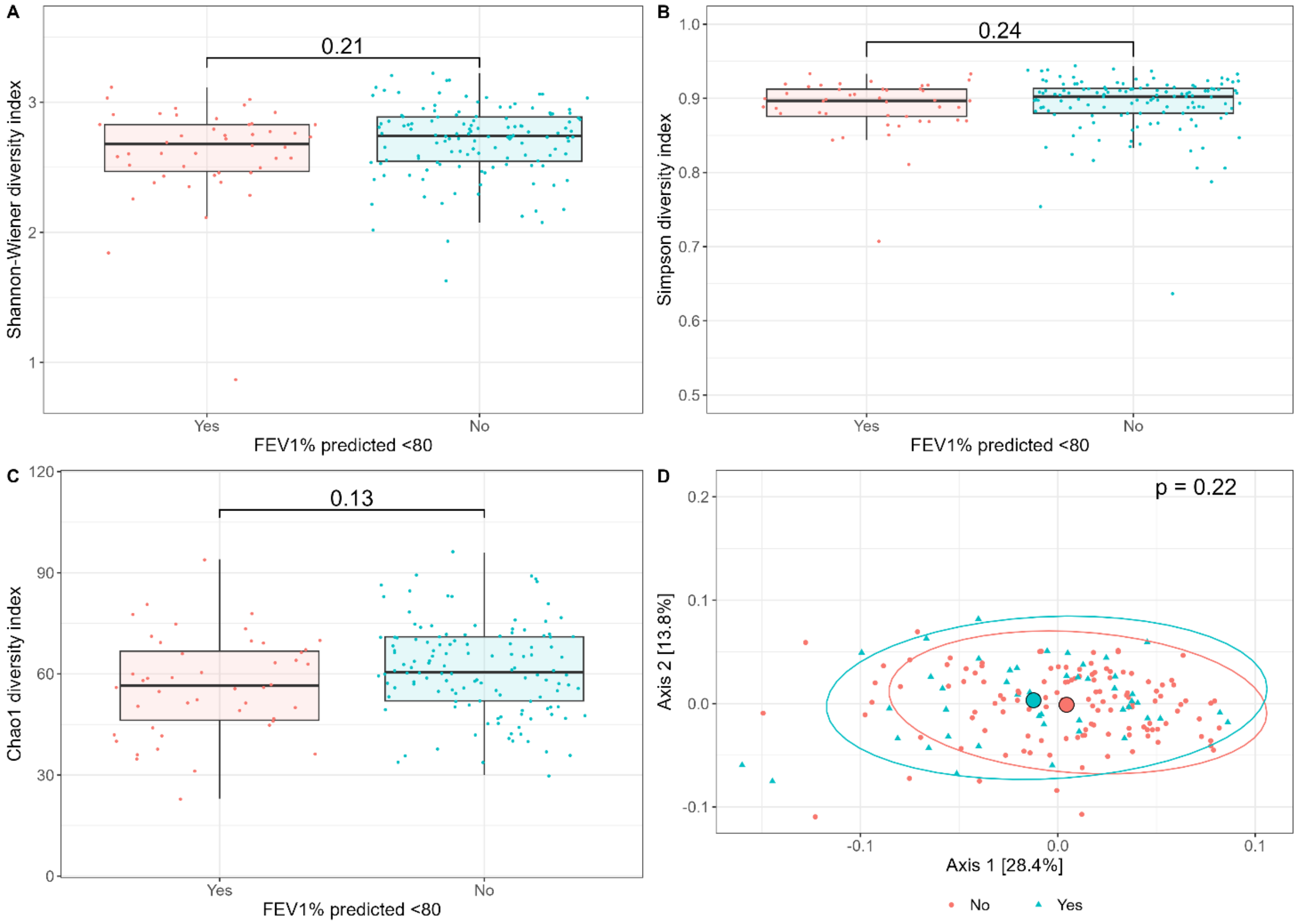

Figure S15 Comparison of sputum microbiota for participants with FEV1 <80% and >80% predicted. **(A)** Shannon-Wiener diversity index (alpha diversity). **(B)** Simpson diversity index (alpha diversity). **(C)** Chao1 diversity index. **(D)** Weighted UniFrac principal coordinates analysis plot. Large bold circle represents centroid of group.

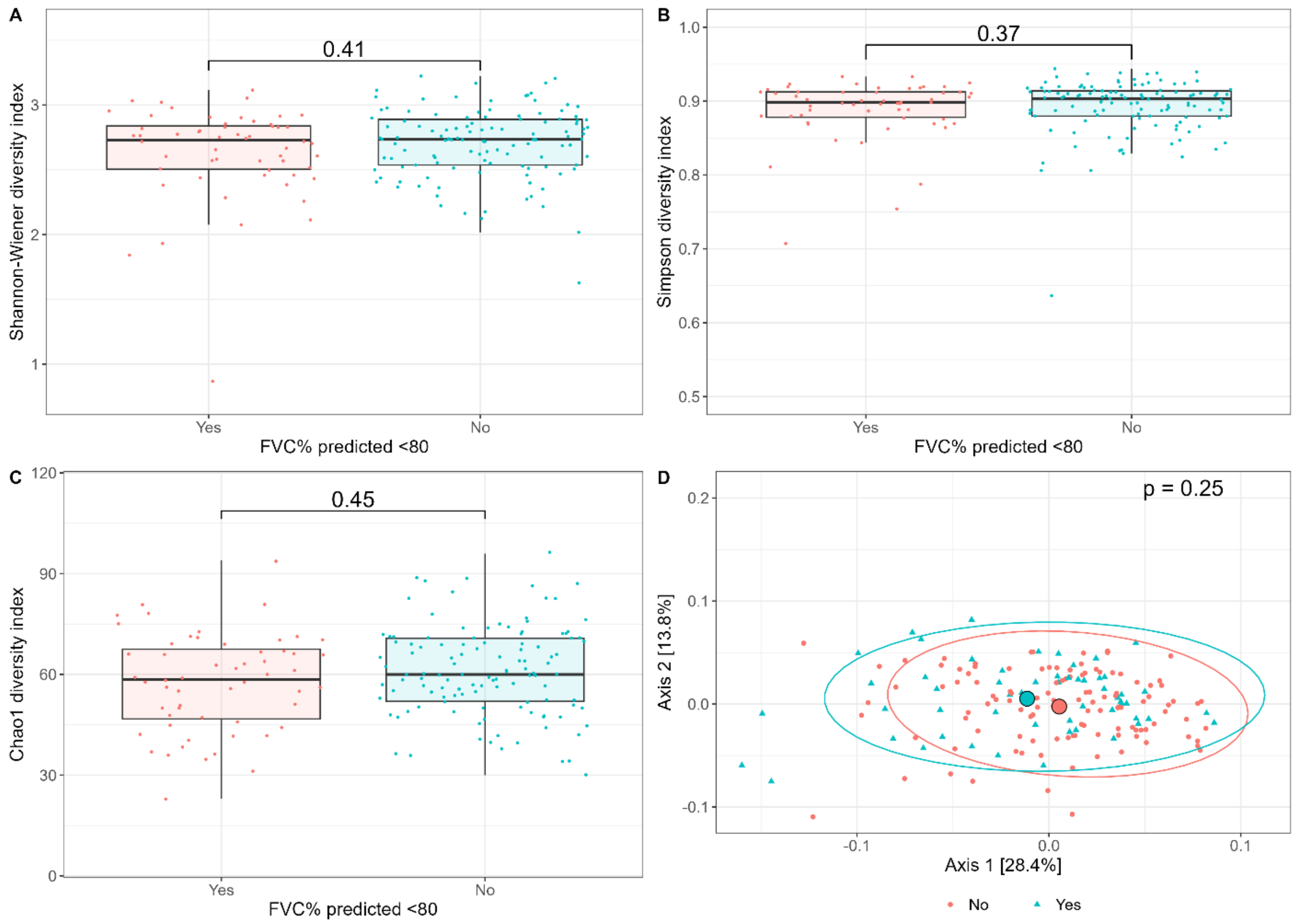

Figure S16 Comparison of sputum microbiota for participants with FVC <80% and >80 % predicted. **(A)** Shannon-Wiener diversity index (alpha diversity). **(B)** Simpson diversity index (alpha diversity). **(C)** Chao1 diversity index. **(D)** Weighted UniFrac principal coordinates analysis plot. Large bold circle represents centroid of group.

Table S1. Criteria for LC symptom groups assignment.

Symptom group	Questionnaire	Response type	Threshold
**Cardiopulmonary**	MRC dyspnoea scale	Numerical scale	Score >1
	Dyspnoea-12	Numerical scale	Score >=12
	Chest pain	Yes/No	Yes
	Chest tightness	Yes/No	Yes
	Palpitations	Yes/No	Yes
	Leg or ankle swelling	Yes/No	Yes
	Leicester Cough physical domain	Numerical scale	Score >5
**Fatigue**	FACIT Fatigue scale	Numerical scale	Score < 40
**Mental health**	PHQ-9	Numerical scale	Score >=10
	GAD-7	Numerical scale	Score >8
**Neurocognitive**	GAD-7	Numerical scale	Score >8
	Brief pain inventory severity	Numerical scale	Score >25
	Montreal Cognitive Assessment (MoCA)	Numerical scale	Score <26
	Pittsburgh sleep quality index	Numerical scale	Score >16
	A worsening in health state from pre-COVID 19 (EQ-5D-5L)	No change/Better/Worse	Worse
**GI**	Stomach pain	Yes/No	Yes
	Constipation	Yes/No	Yes
	Diarrhoea	Yes/No	Yes
	Weight loss	Yes/No	Yes
	Loss of appetite	Yes/No	Yes
	Nausea and/or vomiting	Yes/No	Yes
**Recovered**	Any LC symptom (above)	Yes/No	No
	Do you feel fully recovered from COVID-19?	Yes/No/Not sure	Yes

Table S2. ASVs removed from oral microbiota analysis by Decontam

ASV	Taxonomy (Species)	Present in n samples (% of total samples)	Present in n controls (% of total controls)
TGGGGAATATTGGACAATGGGCGAAAGCCTGATCCAGCCATGCCGCGTGTGTGAAGAAGGTCTTCGGATTGTAAAGCACTTTAAGTTGGGAGGAAGGGCAGTTACCTAATACGTAATTGTTTTGACGTTACCGACAGAATAAGCACCGGCTAACTCTGTGCCAGCAGCCGCGGTAATACAGAGGGTGCAAGCGTTAATCGGAATTACTGGGCGTAAAGCGCGCGTAGGTGGTTTGTTAAGTTGGATGTGAAATCCCCGGGCTCAACCTGGGAACTGCATTCAAAACTGACTGACTAGAGTATGGTAGAGGGTGGTGGAATTTCCTGTGTAGCGGTGAAATGCGTAGATATAGGAAGGAACACCAGTGGCGAAGGCGACCACCTGGACTAATACTGACACTGAGGTGCGAAAGCGTGGGGAGCAAACA	*Pseudomonas fluorescens/poae*	74 (9.1%)	43 (22.4%)
TGGGGAATATTGGACAATGGGCGAAAGCCTGATCCAGCAATGCCGCGTGAGTGATGAAGGCCTTAGGGTTGTAAAGCTCTTTTACCCGGGATGATAATGACAGTACCGGGAGAATAAGCTCCGGCTAACTCCGTGCCAGCAGCCGCGGTAATACGGAGGGAGCTAGCGTTATTCGGAATTACTGGGCGTAAAGCGCACGTAGGCGGCTTTGTAAGTTAGAGGTGAAAGCCTGGAGCTCAACTCCAGAATTGCCTTTAAGACTGCATCGCTTGAATCCAGGAGAGGTGAGTGGAATTCCGAGTGTAGAGGTGAAATTCGTAGATATTCGGAAGAACACCAGTGGCGAAGGCGGCTCACTGGACTGGTATTGACGCTGAGGTGCGAAAGCGTGGGGAGCAAACA	*Sphingomonas alpina/echinoides/mali/oligophenolica/rhizogenes/sanxanigenens*	47 (5.8%)	34 (17.7%)
TGGGGAATTTTGGACAATGGGCGCAAGCCTGATCCAGCCATGCCGCGTGCAGGATGAAGGCCTTCGGGTTGTAAACTGCTTTTGTACGGAACGAAAAGGTTTGACTTAATACGTTGGACTCATGACGGTACCGTAAGAATAAGCACCGGCTAACTACGTGCCAGCAGCCGCGGTAATACGTAGGGTGCGAGCGTTAATCGGAATTACTGGGCGTAAAGCGTGCGCAGGCGGTTATGTAAGACAGATGTGAAATCCCCGGGCTCAACCTGGGAACTGCATTAGTGACTGCATAGCTGGAGTACGGCAGAGGGGGATGGAATTCCGCGTGTAGCAGTGAAATGCGTAGATATGCGGAGGAACACCGATGGCGAAGGCAATCCCCTGGGCCTGTACTGACGCTCATGCACGAAAGCGTGGGGAGCAAACA	*Curvibacter gracilis*	36 (4.4%)	35 (18.2%)
TGGGGAATATTGGACAATGGGCGAAAGCCTGATCCAGCCATGCCGCGTGTGTGAAGAAGGTCTTCGGATTGTAAAGCACTTTAAGTTGGGAGGAAGGGTTGTAGATTAATACTCTGCAATTTTGACGTTACCGACAGAATAAGCACCGGCTAACTCTGTGCCAGCAGCCGCGGTAATACAGAGGGTGCAAGCGTTAATCGGAATTACTGGGCGTAAAGCGCGCGTAGGTGGTTTGTTAAGTTGGATGTGAAATCCCCGGGCTCAACCTGGGAACTGCATTCAAAACTGACTGACTAGAGTATGGTAGAGGGTGGTGGAATTTCCTGTGTAGCGGTGAAATGCGTAGATATAGGAAGGAACACCAGTGGCGAAGGCGACCACCTGGACTAATACTGACACTGAGGTGCGAAAGCGTGGGGAGCAAACA	*Pseudomonas azotoformans/brenneri/cedrina/cf./fluorescens/gessardii/grimontii/lactis/libanensis/marginalis/paralactis/poae/putida/reactans/syncyanea/synxantha*	13 (1.6%)	8 (4.2%)

TGGGGAATTTTGGACAATGGGGGCAACCCTGATCCAGCAATGCCGCGTGAGTGAAGAAGGCCTTCGGGTTGTAAAGCTCTTTTGTCAGGGAAGAAACGGTAGTAGCTAATATCTATTACTAATGACGGTACCTGAAGAATAAGCACCGGCTAACTACGTGCCAGCAGCCGCGGTAATACGTAGGGTGCAAGCGTTAATCGGAATTACTGGGCGTAAAGCGTGCGCAGGCGGTTGTGTAAGTCAGATGTGAAATCCCCGGGCTCAACCTGGGAATTGCATTTGAGACTGCACGGCTAGAGTGTGTCAGAGGGGGGTAGAATTCCACGTGTAGCAGTGAAATGCGTAGATATGTGGAGGAATACCGATGGCGAAGGCAGCCCCCTGGGATAACACTGACGCTCATGCACGAAAGCGTGGGGAGCAAACA	*Herbaspirillum aquaticum/huttiense*	3 (0.4%)	9 (4.7%)
TGGGGAATATTGGACAATGGGCGAAAGCCTGATCCAGCCATGCCGCGTGTGTGAAGAAGGTCTTCGGATTGTAAAGCACTTTAAGTTGGGAGGAAGGGCAGTAAATTAATACTTTGCTGTTTTGACGTTACCGACAGAATAAGCACCGGCTAACTCTGTGCCAGCAGCCGCGGTAATACAGAGGGTGCAAGCGTTAATCGGAATTACTGGGCGTAAAGCGCGCGTAGGTGGTTCGTTAAGTTGGATGTGAAATCCCCGGGCTCAACCTGGGAACTGCATTCAAAACTGACGAGCTAGAGTATGGTAGAGGGTGGTGGAATTTCCTGTGTAGCGGTGAAATGCGTAGATATAGGAAGGAACACCAGTGGCGAAGGCGACCACCTGGACTGATACTGACACTGAGGTGCGAAAGCGTGGGGAGCAAACA	*Pseudomonas brenneri/collierea/fluorescens/libanensis/migulae/panacis/proteolytica/putida/veronii/yamanorum*	6 (0.7%)	84 (43.8%)
TGGGGAATTTTCCGCAATGGGCGAAAGCCTGACGGAGCAATGCCGCGTGGAGGTAGAAGGCCTACGGGTCGTGAACTTCTTTTCCCGGAGAAGAAGCAATGACGGTATCTGGGGAATAAGCATCGGCTAACTCTGTGCCAGCAGCCGCGGTAATACAGAGGATGCAAGCGTTATCCGGAATGATTGGGCGTAAAGCGTCTGTAGGTGGCTTTTTAAGTCCGCCGTCAAATCCCAGGGCTCAACCCTGGACAGGCGGTGGAAACTACCAAGCTGGAGTACGGTAGGGGCAGAGGGAATTTCCGGTGGAGCGGTGAAATGCGTAGAGATCGGAAAGAACACCAACGGCGAAAGCACTCTGCTGGGCCGACACTGACACTGAGAGACGAAAGCTAGGGGAGCGAATG	Unclassified *Chloroplast*	7 (0.9%)	3 (1.6%)
TGGGGAATTTTGGACAATGGGCGAAAGCCTGATCCAGCAATGCCGCGTGCAGGATGAAGGCCTTCGGGTTGTAAACTGCTTTTGTACGGAACGAAAAAGCTCCTTCTAATACAGGGGGCCCATGACGGTACCGTAAGAATAAGCACCGGCTAACTACGTGCCAGCAGCCGCGGTAATACGTAGGGTGCGAGCGTTAATCGGAATTACTGGGCGTAAAGCGTGCGCAGGCGGTTATGTAAGACAGATGTGAAATCCCCGGGCTCAACCTGGGAACTGCATTTGTGACTGCATGGCTAGAGTACGGTAGAGGGGGATGGAATTCCGCGTGTAGCAGTGAAATGCGTAGATATGCGGAGGAACACCGATGGCGAAGGCAATCCCCTGGACCTGTACTGACGCTCATGCACGAAAGCGTGGGGAGCAAACA	*Delftia acidovorans/lacustris/tsuruhatensis*	3 (0.4%)	6 (3.1%)
TGGGGAATATTGCACAATGGGCGAAAGCCTGATGCAGCGACGCCGCGTGAGGGATGACGGCCTTCGGGTTGTAAACCTCTTTCAGCAGGGACGAAGCGCAAGTGACGGTACCTGCAGAAGAAGCACCGGCTAACTACGTGCCAGCAGCCGCGGTAATACGTAGGGTGCAAGCGTTGTCCGGAATTACTGGGCGTAAAGAGTTCGTAGGCGGTTTGTCGCGTCGTTTGTGAAAACCAGCAGCTCAACTGCTGGCTTGCAGGCGATACGGGCAGACTTGAGTACTGCAGGGGAGACTGGAATTCCTGGTGTAGCGGTGAAATGCGCAGATATCAGGAGGAACACCGGTGGCGAAGGCGGGTCTCTGGGCAGTAACTGACGCTGAGGAACGAAAGCGTGGGTAGCGAACA	*Rhodococcus baikonurensis/boritolerans/degradans/erythropolis/globerulus/hoagii/opacus/qingshengii/rhodochrous*	4 (0.5%)	6 (3.1%)

TGGGGAATATTGCACAATGGGCGCAAGCCTGATGCAGCCATGCCGCGTGTATGAAGAAGGCCTTCGGGTTGTAAAGTACTTTCAGCGGGGAGGAAGGGAGTAAAGTTAATACCTTTGCTCATTGACGTTACCCGCAGAAGAAGCACCGGCTAACTCCGTGCCAGCAGCCGCGGTAATACGGAGGGTGCAAGCGTTAATCGGAATTACTGGGCGTAAAGCGCACGCAGGCGGTTTGTTAAGTCAGATGTGAAATCCCCGGGCTCAACCTGGGAACTGCATCTGATACTGGCAAGCTTGAGTCTCGTAGAGGGGGGTAGAATTCCAGGTGTAGCGGTGAAATGCGTAGAGATCTGGAGGAATACCGGTGGCGAAGGCGGCCCCCTGGACGAAGACTGACGCTCAGGTGCGAAAGCGTGGGGAGCAAACA	*Escherichia-Shigella albertii/boydii/coli/dysenteriae/fergusonii/flexneri/marmotae/sonnei*	20 (2.5%)	65 (33.9%)

Table S3. ASVs removed from sputum microbiota analysis by Decontam

ASV	Taxonomy (Species)	Present in n samples (% of total samples)	Present in n controls (% of total controls)
TGGGGAATATTGGACAATGGGCGAAAGCCTGATCCAGCCATGCCGCGTGTGTGAAGAAGGTCT<br>TCGGATTGTAAAGCACTTTAAGTTGGGAGGAAGGGCAGTTACCTAATACGTAATTGTTTTGAC<br>GTTACCGACAGAATAAGCACCGGCTAACTCTGTGCCAGCAGCCGCGGTAATACAGAGGGTGC<br>AAGCGTTAATCGGAATTACTGGGCGTAAAGCGCGCGTAGGTGGTTTGTTAAGTTGGATGTGAA<br>ATCCCCGGGCTCAACCTGGGAACTGCATTCAAAACTGACTGACTAGAGTATGGTAGAGGGTGG<br>TGGAATTTCCTGTGTAGCGGTGAAATGCGTAGATATAGGAAGGAACACCAGTGGCGAAGGCG<br>ACCACCTGGACTAATACTGACACTGAGGTGCGAAAGCGTGGGGAGCAAACA	*Pseudomonas fluorescens/poae*	87 (19.6%)	12 (27.3%)
TGGGGAATTTTGGACAATGGGCGCAAGCCTGATCCAGCCATGCCGCGTGCAGGATGAAGGCCT<br>TCGGGTTGTAAACTGCTTTTGTACGGAACGAAAAGGTTTGACTTAATACGTTGGACTCATGAC<br>GGTACCGTAAGAATAAGCACCGGCTAACTACGTGCCAGCAGCCGCGGTAATACGTAGGGTGC<br>GAGCGTTAATCGGAATTACTGGGCGTAAAGCGTGCGCAGGCGGTTATGTAAGACAGATGTGAA<br>ATCCCCGGGCTCAACCTGGGAACTGCATTAGTGACTGCATAGCTGGAGTACGGCAGAGGGGG<br>ATGGAATTCCGCGTGTAGCAGTGAAATGCGTAGATATGCGGAGGAACACCGATGGCGAAGGC<br>AATCCCCTGGGCCTGTACTGACGCTCATGCACGAAAGCGTGGGGAGCAAACA	*Curvibacter gracilis*	40 (9.0%)	10 (22.7%)
TGGGGAATATTGGACAATGGGCGAAAGCCTGATCCAGCAATGCCGCGTGAGTGATGAAGGCC<br>TTAGGGTTGTAAAGCTCTTTTACCCGGGATGATAATGACAGTACCGGGAGAATAAGCTCCGGC<br>TAACTCCGTGCCAGCAGCCGCGGTAATACGGAGGGAGCTAGCGTTATTCGGAATTACTGGGCG<br>TAAAGCGCACGTAGGCGGCTTTGTAAGTTAGAGGTGAAAGCCTGGAGCTCAACTCCAGAATTG<br>CCTTTAAGACTGCATCGCTTGAATCCAGGAGAGGTGAGTGGAATTCCGAGTGTAGAGGTGAAA<br>TTCGTAGATATTCGGAAGAACACCAGTGGCGAAGGCGGCTCACTGGACTGGTATTGACGCTGA<br>GGTGCGAAAGCGTGGGGAGCAAACA	*Sphingomonas alpina/echinoides/mali/oligophenolica/rhizogenes/sanxanigenens*	41 (9.3%)	9 (20.5%)
TGGGGAATATTGGACAATGGGCGAAAGCCTGATCCAGCCATGCCGCGTGTGTGAAGAAGGTCT<br>TCGGATTGTAAAGCACTTTAAGTTGGGAGGAAGGGCAGTAAATTAATACTTTGCTGTTTTGAC<br>GTTACCGACAGAATAAGCACCGGCTAACTCTGTGCCAGCAGCCGCGGTAATACAGAGGGTGC<br>AAGCGTTAATCGGAATTACTGGGCGTAAAGCGCGCGTAGGTGGTTCGTTAAGTTGGATGTGAA<br>ATCCCCGGGCTCAACCTGGGAACTGCATTCAAAACTGACGAGCTAGAGTATGGTAGAGGGTGG<br>TGGAATTTCCTGTGTAGCGGTGAAATGCGTAGATATAGGAAGGAACACCAGTGGCGAAGGCG<br>ACCACCTGGACTGATACTGACACTGAGGTGCGAAAGCGTGGGGAGCAAACA	*Pseudomonas brenneri/collierea/fluorescens/libanensis/migulae/panacis/proteolytica/putida/veronii/yamanorum*	10 (2.3%)	42 (95.5%)

TGGGGAATATTGGACAATGGGCGAAAGCCTGATCCAGCCATGCCGCGTGTGTGAAGAAGGTCT<br>TCGGATTGTAAAGCACTTTAAGTTGGGAGGAAGGGTTGTAGATTAATACTCTGCAATTTTGAC<br>GTTACCGACAGAATAAGCACCGGCTAACTCTGTGCCAGCAGCCGCGGTAATACAGAGGGTGC<br>AAGCGTTAATCGGAATTACTGGGCGTAAAGCGCGCGTAGGTGGTTTGTTAAGTTGGATGTGAA<br>ATCCCCGGGCTCAACCTGGGAACTGCATTCAAAACTGACTGACTAGAGTATGGTAGAGGGTGG<br>TGGAATTTCCTGTGTAGCGGTGAAATGCGTAGATATAGGAAGGAACACCAGTGGCGAAGGCG<br>ACCACCTGGACTAATACTGACACTGAGGTGCGAAAGCGTGGGGAGCAAACA	*Pseudomonas azotoformans/brenneri/cedrina/cf./fluorescens/gessardii/grimontii/lactis/libanensis/marginalis/paralactis/poae/putida/reactans/syncyanea/synxantha*	9 (2.0%)	10 (22.7%)
TGGGGAATATTGCACAATGGGCGCAAGCCTGATGCAGCCATGCCGCGTGTATGAAGAAGGCCT<br>TCGGGTTGTAAAGTACTTTCAGCGGGGAGGAAGGGAGTAAAGTTAATACCTTTGCTCATTGAC<br>GTTACCCGCAGAAGAAGCACCGGCTAACTCCGTGCCAGCAGCCGCGGTAATACGGAGGGTGC<br>AAGCGTTAATCGGAATTACTGGGCGTAAAGCGCACGCAGGCGGTTTGTTAAGTCAGATGTGAA<br>ATCCCCGGGCTCAACCTGGGAACTGCATCTGATACTGGCAAGCTTGAGTCTCGTAGAGGGGGG<br>TAGAATTCCAGGTGTAGCGGTGAAATGCGTAGAGATCTGGAGGAATACCGGTGGCGAAGGCG<br>GCCCCCTGGACGAAGACTGACGCTCAGGTGCGAAAGCGTGGGGAGCAAACA	*Escherichia-Shigella albertii/boydii/coli/dysenteriae/fergusonii/flexneri/marmotae/sonnei*	26 (5.9%)	42 (95.5%)
TAGGGAATCTTCCGCAATGGGCGAAAGCCTGACGGAGCAACGCCGCGTGAGTGAAGAAGGTC<br>TTCGGATCGTAAAACTCTGTTATTAGGGAAGAACAAATGTGTAAGTAACTATGCACGTCTTGA<br>CGGTACCTAATCAGAAAGCCACGGCTAACTACGTGCCAGCAGCCGCGGTAATACGTAGGTGG<br>CAAGCGTTATCCGGAATTATTGGGCGTAAAGCGCGCGTAGGCGGTTTTTTAAGTCTGATGTGA<br>AAGCCCACGGCTCAACCGTGGAGGGTCATTGGAAACTGGAAAACTTGAGTGCAGAAGAGGAA<br>AGTGGAATTCCATGTGTAGCGGTGAAATGCGCAGAGATATGGAGGAACACCAGTGGCGAAGG<br>CGACTTTCTGGTCTGTAACTGACGCTGATGTGCGAAAGCGTGGGGATCAAACA	*Staphylococcus aureus/capitis/caprae/epidermidis/haemolyticus/saprophyticus/warneri*	2 (0.5%)	1 (2.3%)
TGGGGAATATTGGACAATGGGCGAAAGCCTGATCCAGCCATGCCGCGTGTGTGAAGAAGGTCT<br>TCGGATTGTAAAGCACTTTAAGTTGGGAGGAAGGGCAGTAAGCTAATATCTTGCTGTTTTGAC<br>GTTACCGACAGAATAAGCACCGGCTAACTCTGTGCCAGCAGCCGCGGTAATACAGAGGGTGC<br>AAGCGTTAATCGGAATTACTGGGCGTAAAGCGCGCGTAGGTGGTTTGTTAAGTTGGATGTGAA<br>AGCCCCGGGCTCAACCTGGGAACTGCATCCAAAACTGGCAAGCTAGAGTATGGTAGAGGGTG<br>GTGGAATTTCCTGTGTAGCGGTGAAATGCGTAGATATAGGAAGGAACACCAGTGGCGAAGGC<br>GACCACCTGGACTGATACTGACACTGAGGTGCGAAAGCGTGGGGAGCAAACA	*Pseudomonas aeruginosa/putida*	5 (1.1%)	2 (4.5%)
TAGGGAATCTTCCGCAATGGGCGAAAGCCTGACGGAGCAACGCCGCGTGAGTGATGAAGGTC<br>TTCGGATCGTAAAACTCTGTTATCAGGGAAGAACAAATGTGTAAGTAACTGTGCACATCTTGA<br>CGGTACCTGATCAGAAAGCCACGGCTAACTACGTGCCAGCAGCCGCGGTAATACGTAGGTGG<br>CAAGCGTTATCCGGAATTATTGGGCGTAAAGCGCGCGTAGGCGGTTTTTTAAGTCTGATGTGA<br>AAGCCCACGGCTCAACCGTGGAGGGTCATTGGAAACTGGAAAACTTGAGTGCAGAAGAGGAA<br>AGTGGAATTCCATGTGTAGCGGTGAAATGCGCAGAGATATGGAGGAACACCAGTGGCGAAGG<br>CGACTTTCTGGTCTGTAACTGACGCTGATGTGCGAAAGCGTGGGGATCAAACA	*Staphylococcus aureus/pasteuri/warneri*	1 (0.2%)	1 (2.3%)

Table S4a. Co-occurrence network comparison for oral microbiota ongoing health impairment

Metric	4: Mild	1: Very severe	Absolute difference	Similarity/Distance metric value	p-value	Notes
**Global Network (LCC)**						
Relative LCC size	0.516	0.422	0.094	–	0.73	
Clustering coefficient	0.246	0.220	0.026	–	0.85	
Modularity	0.653	0.510	0.144	–	0.25	
Positive edge percentage	73.171	69.444	3.726	–	0.73	
Edge density	0.078	0.103	0.025	–	0.62	
Natural connectivity	0.038	0.048	0.010	–	0.72	
Vertex connectivity	1.000	1.000	0.000	–	1.00	
Edge connectivity	1.000	1.000	0.000	–	1.00	
Average dissimilarity*	0.975	0.968	0.008	–	0.63	*Dissimilarity = 1 - edge weight
Average path length**	3.690	2.355	1.334	–	0.15	**Path length = Units with average dissimilarity
**Global Network (Whole Network)**						
Number of components	26.000	30.000	4.000	–	0.71	
Clustering coefficient	0.293	0.300	0.007	–	0.96	
Modularity	0.700	0.629	0.071	–	0.31	
Positive edge percentage	77.083	75.556	1.528	–	0.81	
Edge density	0.024	0.022	0.001	–	0.86	
Natural connectivity	0.018	0.018	0.000	–	0.83	
**Jaccard Index (Central Nodes)**						Jaccard index in [0,1]; 1 = perfect agreement
Degree	–	–	–	0.318	P≤: 0.54<br>P≥: 0.64	
Betweenness centrality	–	–	–	0.120	P≤: 0.01*<br>P≥: 1.00	
Closeness centrality	–	–	–	0.333	P≤: 0.60<br>P≥: 0.58	
Eigenvector centrality	–	–	–	0.143	P≤: 0.02*<br>P≥: 1.00	
Hub taxa	–	–	–	0.000	P≤: 0.13<br>P≥: 1.00	

LCC: Largest Connected Component. Significance codes: ***: 0.001, **: 0.01, *: 0.05, .: 0.1

Table S4b. Co-occurrence network centrality measures comparison for oral microbiota ongoing health impairment with hubs identified

	Centrality measure								
	Degree		Betweeness		Closeness		Eigenvector		
**Taxa**	**1: Mild**	**4: Very severe**	**1: Mild**	**4: Very severe**	**1: Mild**	**4: Very severe**	**1: Mild**	**4: Very severe**	**Hub group**
Streptococcus	0.063	0.048	0.274	0.283	0.551	0.701	0.686	0.522	1: Mild
Veillonella	0.095	0.016	0.522	0.000	0.641	0.495	1.000	0.173	1: Mild
Rothia	0.032	0.111	0.000	0.351	0.429	0.775	0.373	0.725	4: Very severe
Limosilactobacillus	0.079	0.111	0.280	0.302	0.449	0.748	0.010	1.000	4: Very severe
Lactobacillus	0.063	0.079	0.063	0.108	0.400	0.644	0.008	0.807	4: Very severe
Dialister	0.000	0.000	0.000	0.000	0.000	0.000	0.000	0.000	None
pneumosintes	0.000	0.000	0.000	0.000	0.000	0.000	0.000	0.000	None
Selenomonas	0.000	0.000	0.000	0.000	0.000	0.000	0.000	0.000	None
Centipeda	0.000	0.000	0.000	0.000	0.000	0.000	0.000	0.000	None
Unclassified Selenomonadaceae	0.000	0.000	0.000	0.000	0.000	0.000	0.000	0.000	None
Atopobium	0.048	0.000	0.498	0.000	0.501	0.000	0.333	0.000	None
Olsenella	0.000	0.000	0.000	0.000	0.000	0.000	0.000	0.000	None
Bifidobacterium	0.048	0.048	0.000	0.000	0.361	0.568	0.007	0.511	None
Scardovia	0.032	0.048	0.000	0.000	0.340	0.558	0.005	0.599	None
Actinomyces	0.063	0.000	0.159	0.000	0.557	0.000	0.693	0.000	None
F0332	0.000	0.000	0.000	0.000	0.000	0.000	0.000	0.000	None
Corynebacterium	0.000	0.000	0.000	0.000	0.000	0.000	0.000	0.000	None
Cardiobacterium	0.000	0.000	0.000	0.000	0.000	0.000	0.000	0.000	None
Neisseria	0.032	0.048	0.063	0.111	0.340	0.551	0.053	0.097	None
Eikenella	0.000	0.000	0.000	0.000	0.000	0.000	0.000	0.000	None
Kingella	0.000	0.000	0.000	0.000	0.000	0.000	0.000	0.000	None
Lautropia	0.000	0.016	0.000	0.000	0.000	0.475	0.000	0.218	None
Haemophilus	0.000	0.016	0.000	0.000	0.000	0.400	0.000	0.023	None
Actinobacillus	0.000	0.000	0.000	0.000	0.000	0.000	0.000	0.000	None
Aggregatibacter	0.016	0.000	0.000	0.000	0.411	0.000	0.259	0.000	None
Treponema	0.063	0.063	0.065	0.345	0.529	0.658	0.492	0.145	None
Lentimicrobium	0.000	0.000	0.000	0.000	0.000	0.000	0.000	0.000	None
Capnocytophaga	0.000	0.048	0.000	0.108	0.000	0.582	0.000	0.111	None
Bergeyella	0.000	0.048	0.000	0.169	0.000	0.599	0.000	0.061	None
Porphyromonas	0.048	0.063	0.365	0.000	0.425	0.623	0.008	0.682	None
F0058	0.016	0.016	0.000	0.000	0.358	0.366	0.095	0.008	None
Alloprevotella	0.016	0.016	0.000	0.000	0.317	0.485	0.002	0.152	None
Prevotella	0.016	0.032	0.000	0.077	0.418	0.473	0.290	0.051	None
Prevotella_7	0.048	0.000	0.508	0.000	0.554	0.000	0.582	0.000	None
Tannerella	0.000	0.032	0.000	0.077	0.000	0.484	0.000	0.034	None
Fretibacterium	0.048	0.063	0.063	0.271	0.458	0.631	0.359	0.059	None

Fusobacterium	0.000	0.048	0.000	0.191	0.000	0.617	0.000	0.202	None
Leptotrichia	0.048	0.032	0.417	0.000	0.446	0.558	0.047	0.182	None
Catonella	0.000	0.000	0.000	0.000	0.000	0.000	0.000	0.000	None
Oribacterium	0.000	0.000	0.000	0.000	0.000	0.000	0.000	0.000	None
Stomatobaculum	0.032	0.000	0.042	0.000	0.382	0.000	0.046	0.000	None
Lachnoanaerobaculum	0.032	0.000	0.000	0.000	0.364	0.000	0.026	0.000	None
Johnsonella	0.032	0.000	0.000	0.000	0.394	0.000	0.291	0.000	None
Unclassified Clostridia UCG-014	0.000	0.000	0.000	0.000	0.000	0.000	0.000	0.000	None
Campylobacter	0.032	0.016	0.000	0.000	0.492	0.367	0.415	0.013	None
Parvimonas	0.000	0.000	0.000	0.000	0.000	0.000	0.000	0.000	None
Filifactor	0.000	0.032	0.000	0.077	0.000	0.474	0.000	0.015	None
Peptostreptococcus	0.032	0.000	0.387	0.000	0.411	0.000	0.015	0.000	None
[Eubacterium] brachy group	0.016	0.000	0.000	0.000	0.335	0.000	0.100	0.000	None
[Eubacterium] nodatum group	0.000	0.000	0.000	0.000	0.000	0.000	0.000	0.000	None
[Eubacterium] saphenum group	0.000	0.016	0.000	0.000	0.000	0.360	0.000	0.003	None
Mycoplasma	0.000	0.016	0.000	0.000	0.000	0.433	0.000	0.013	None
Solobacterium	0.016	0.000	0.000	0.000	0.384	0.000	0.205	0.000	None
Gemella	0.032	0.000	0.121	0.000	0.413	0.000	0.174	0.000	None
Granulicatella	0.048	0.000	0.181	0.000	0.504	0.000	0.421	0.000	None
Abiotrophia	0.016	0.000	0.000	0.000	0.269	0.000	0.015	0.000	None
HT002	0.016	0.048	0.000	0.077	0.294	0.555	0.003	0.367	None
Unclassified Saccharimonadales	0.000	0.000	0.000	0.000	0.000	0.000	0.000	0.000	None
Candidatus Saccharimonas	0.032	0.000	0.000	0.000	0.329	0.000	0.005	0.000	None
Unclassified Saccharimonadaceae	0.048	0.016	0.093	0.000	0.491	0.450	0.332	0.033	None
TM7x	0.000	0.000	0.000	0.000	0.000	0.000	0.000	0.000	None
Unclassified Absconditabacteriales (SR1)	0.000	0.000	0.000	0.000	0.000	0.000	0.000	0.000	None
Megasphaera	0.048	0.000	0.504	0.000	0.472	0.000	0.122	0.000	None
Anaeroglobus	0.000	0.000	0.000	0.000	0.000	0.000	0.000	0.000	None

Table S5a. Co-occurrence network comparison for sputum microbiota ongoing health impairment

Metric	4: Mild	1: Very severe	Absolute difference	Similarity/Distance metric value	p-value	Notes
**Global Network (LCC)**						
Relative LCC size	0.147	0.397	0.250	–	0.24	
Clustering coefficient	0.232	0.045	0.187	–	0.42	
Modularity	0.405	0.555	0.150	–	0.43	
Positive edge percentage	100.000	63.333	36.667	–	0.04 *	
Edge density	0.222	0.085	0.137	–	0.27	
Natural connectivity	0.145	0.047	0.098	–	0.23	
Vertex connectivity	1.000	1.000	0.000	–	1.00	
Edge connectivity	1.000	1.000	0.000	–	1.00	
Average dissimilarity*	0.926	0.973	0.048	–	0.22	*Dissimilarity = 1 - edge weight
Average path length**	2.104	2.612	0.508	–	0.62	**Path length = Units with average dissimilarity
**Global Network (Whole Network)**						
Number of components	40.000	33.000	7.000	–	0.63	
Clustering coefficient	0.261	0.037	0.224	–	0.16	
Modularity	0.754	0.689	0.066	–	0.41	
Positive edge percentage	80.645	64.103	16.543	–	0.16	
Edge density	0.014	0.017	0.004	–	0.63	
Natural connectivity	0.016	0.017	0.000	–	0.66	
**Jaccard Index (Central Nodes)**						Jaccard index in [0,1]; 1 = perfect agreement
Degree	–	–	–	0.333	P≤: 0.61<br>P≥: 0.59	
Betweenness centrality	–	–	–	0.188	P≤: 0.17<br>P≥: 0.94	
Closeness centrality	–	–	–	0.227	P≤: 0.21<br>P≥: 0.90	
Eigenvector centrality	–	–	–	0.125	P≤: 0.02 *<br>P≥: 1.00	
Hub taxa	–	–	–	0.100	P≤: 0.10<br>P≥: 1.00	

LCC: Largest Connected Component. Significance codes: ***: 0.001, **: 0.01, *: 0.05, .: 0.1

Table 5b. Co-occurrence network centrality measures comparison for sputum microbiota ongoing health impairment with hubs identified

	Centrality measure								
	Degree		Betweeness		Closeness		Eigenvector		
Taxa	1: Mild	4: Very severe	1: Mild	4: Very severe	1: Mild	4: Very severe	1: Mild	4: Very severe	Hub group
Streptococcus	0.045	0.045	0.556	0.209	0.843	0.645	0.800	0.699	Both
Veillonella	0.045	0.000	0.556	0.000	0.856	0.000	1.000	0.000	1: Mild
Actinomyces	0.045	0.060	0.500	0.495	0.807	0.621	0.830	0.156	1: Mild
Prevotella_7	0.030	0.045	0.389	0.212	0.696	0.533	0.421	0.081	1: Mild
Solobacterium	0.030	0.000	0.222	0.000	0.615	0.000	0.198	0.000	1: Mild
Granulicatella	0.030	0.015	0.222	0.000	0.663	0.419	0.378	0.176	1: Mild
Bifidobacterium	0.000	0.045	0.000	0.129	0.000	0.621	0.000	0.548	4: Very severe
Rothia	0.015	0.075	0.000	0.222	0.571	0.619	0.340	0.605	4: Very severe
Staphylococcus	0.000	0.060	0.000	0.058	0.000	0.593	0.000	0.604	4: Very severe
Limosilactobacillus	0.000	0.134	0.000	0.732	0.000	0.817	0.000	1.000	4: Very severe
Unclassified Selenomonadaceae	0.000	0.015	0.000	0.000	0.000	0.433	0.000	0.159	None
Centipeda	0.000	0.000	0.000	0.000	0.000	0.000	0.000	0.000	None
Selenomonas	0.000	0.000	0.000	0.000	0.000	0.000	0.000	0.000	None
Fretibacterium	0.000	0.000	0.000	0.000	0.000	0.000	0.000	0.000	None
Neisseria	0.000	0.015	0.000	0.000	0.000	0.504	0.000	0.278	None
Kingella	0.000	0.000	0.000	0.000	0.000	0.000	0.000	0.000	None
Eikenella	0.000	0.000	0.000	0.000	0.000	0.000	0.000	0.000	None
Lautropia	0.000	0.015	0.000	0.000	0.000	0.506	0.000	0.283	None
Aggregatibacter	0.000	0.030	0.000	0.471	0.000	0.615	0.000	0.317	None
Haemophilus	0.015	0.000	0.000	0.000	0.489	0.000	0.151	0.000	None
Actinobacillus	0.000	0.000	0.000	0.000	0.000	0.000	0.000	0.000	None
Cardiobacterium	0.000	0.000	0.000	0.000	0.000	0.000	0.000	0.000	None
Scardovia	0.000	0.030	0.000	0.052	0.000	0.478	0.000	0.199	None
F0332	0.000	0.000	0.000	0.000	0.000	0.000	0.000	0.000	None
Corynebacterium	0.000	0.000	0.000	0.000	0.000	0.000	0.000	0.000	None
Atopobium	0.000	0.015	0.000	0.000	0.000	0.369	0.000	0.017	None
Olsenella	0.000	0.015	0.000	0.000	0.000	0.510	0.000	0.297	None
Campylobacter	0.030	0.030	0.000	0.000	0.688	0.480	0.703	0.061	None
Unclassified Absconditabacteriales (SR1)	0.000	0.015	0.000	0.000	0.000	0.380	0.000	0.023	None
TM7x	0.000	0.000	0.000	0.000	0.000	0.000	0.000	0.000	None
Unclassified Saccharimonadales	0.000	0.000	0.000	0.000	0.000	0.000	0.000	0.000	None
Candidatus Saccharimonas	0.000	0.000	0.000	0.000	0.000	0.000	0.000	0.000	None
Unclassified Saccharimonadaceae	0.000	0.000	0.000	0.000	0.000	0.000	0.000	0.000	None
Treponema	0.000	0.000	0.000	0.000	0.000	0.000	0.000	0.000	None
Mycoplasma	0.000	0.000	0.000	0.000	0.000	0.000	0.000	0.000	None
Bergeyella	0.000	0.000	0.000	0.000	0.000	0.000	0.000	0.000	None
Capnocytophaga	0.000	0.000	0.000	0.000	0.000	0.000	0.000	0.000	None

Rikenellaceae RC9 gut group	0.000	0.000	0.000	0.000	0.000	0.000	0.000	0.000	None
Lentimicrobium	0.000	0.000	0.000	0.000	0.000	0.000	0.000	0.000	None
Porphyromonas	0.000	0.030	0.000	0.015	0.000	0.462	0.000	0.226	None
Prevotella	0.015	0.045	0.000	0.077	0.478	0.454	0.092	0.046	None
Alloprevotella	0.000	0.015	0.000	0.000	0.000	0.504	0.000	0.279	None
Tannerella	0.000	0.000	0.000	0.000	0.000	0.000	0.000	0.000	None
F0058	0.000	0.000	0.000	0.000	0.000	0.000	0.000	0.000	None
Oceanivirga	0.000	0.000	0.000	0.000	0.000	0.000	0.000	0.000	None
Leptotrichia	0.000	0.000	0.000	0.000	0.000	0.000	0.000	0.000	None
Fusobacterium	0.000	0.015	0.000	0.000	0.000	0.342	0.000	0.015	None
Unclassified Clostridia vadinBB60 group	0.000	0.000	0.000	0.000	0.000	0.000	0.000	0.000	None
Unclassified Clostridia UCG-014	0.000	0.000	0.000	0.000	0.000	0.000	0.000	0.000	None
Butyrivibrio	0.000	0.000	0.000	0.000	0.000	0.000	0.000	0.000	None
Oribacterium	0.000	0.015	0.000	0.000	0.000	0.370	0.000	0.017	None
Stomatobaculum	0.000	0.000	0.000	0.000	0.000	0.000	0.000	0.000	None
Lachnoanaerobaculum	0.000	0.000	0.000	0.000	0.000	0.000	0.000	0.000	None
Johnsonella	0.000	0.000	0.000	0.000	0.000	0.000	0.000	0.000	None
Catonella	0.000	0.015	0.000	0.000	0.000	0.417	0.000	0.171	None
Parvimonas	0.000	0.000	0.000	0.000	0.000	0.000	0.000	0.000	None
Filifactor	0.000	0.000	0.000	0.000	0.000	0.000	0.000	0.000	None
Peptostreptococcus	0.000	0.015	0.000	0.000	0.000	0.417	0.000	0.171	None
[Eubacterium] nodatum group	0.000	0.000	0.000	0.000	0.000	0.000	0.000	0.000	None
[Eubacterium] brachy group	0.000	0.000	0.000	0.000	0.000	0.000	0.000	0.000	None
Peptococcus	0.000	0.000	0.000	0.000	0.000	0.000	0.000	0.000	None
Abiotrophia	0.000	0.000	0.000	0.000	0.000	0.000	0.000	0.000	None
Lactobacillus	0.000	0.030	0.000	0.108	0.000	0.577	0.000	0.411	None
Gemella	0.000	0.015	0.000	0.000	0.000	0.502	0.000	0.272	None
Dialister	0.000	0.000	0.000	0.000	0.000	0.000	0.000	0.000	None
Unclassified Veillonellaceae	0.000	0.000	0.000	0.000	0.000	0.000	0.000	0.000	None
Megasphaera	0.000	0.045	0.000	0.151	0.000	0.500	0.000	0.055	None
Anaeroglobus	0.000	0.000	0.000	0.000	0.000	0.000	0.000	0.000	None

Table S6. Correlation coefficient, p value and FDR adjusted p value for correlation of oral microbiota very severe ongoing health impairment cluster taxa co-occurrence network hubs with inflammation related protein levels (NPX). Only showing correlations with $p < 0.05$ and absolute r^2 of >0.4.

Genus	Inflammation related protein	r^2	p-value	Adjusted p-value (FDR)
Lactobacillus	IL17A	0.441	0.000	0.000
Streptococcus	CLEC4D	0.402	0.000	0.000

Table S7. Correlation coefficient, p value and FDR adjusted p value for correlation of sputum microbiota very severe ongoing health impairment cluster taxa co-occurrence network hubs with inflammation related protein levels (NPX). Only showing correlations with $p < 0.05$ and absolute r^2 of >0.4.

Genus	Inflammation related protein	r^2	p-value	Adjusted p-value (FDR)
Streptococcus	CLSTN2	0.680	0.000	0.000
Streptococcus	CCL21	0.576	0.000	0.003
Streptococcus	CKMT1A_CKMT1B	0.576	0.000	0.003
Solobacterium	CCL11	-0.564	0.000	0.004
Solobacterium	HLA.DRA	-0.589	0.000	0.005
Solobacterium	CXCL17	-0.549	0.000	0.01
Streptococcus	CCL25	0.549	0.000	0.01
Streptococcus	CXCL14	0.548	0.001	0.01
Streptococcus	CD58	0.535	0.001	0.01
Rothia	ISM1	0.526	0.001	0.01
Solobacterium	IL6	-0.516	0.001	0.01
Streptococcus	FCAR	0.516	0.001	0.01
Actinomyces	CCL20	-0.511	0.001	0.02
Streptococcus	TNFRSF11B	0.507	0.001	0.02
Streptococcus	IL17D	0.508	0.002	0.02
Solobacterium	IL5RA	-0.499	0.002	0.02
Rothia	SMOC2	0.499	0.002	0.02
Streptococcus	MATN2	0.498	0.002	0.02
Streptococcus	CXCL17	0.496	0.002	0.02
Actinomyces	LAP3	0.502	0.002	0.02
Actinomyces	CCL26	-0.495	0.002	0.02
Limosilactobacillus	AMBN	0.491	0.002	0.03
Rothia	CXADR	0.484	0.002	0.03
Streptococcus	LTBR	0.481	0.003	0.03
Actinomyces	EPCAM	0.477	0.003	0.03
Streptococcus	TGFA	0.474	0.003	0.03
Solobacterium	REG4	-0.474	0.003	0.03
Veillonella	LY6D	0.472	0.003	0.03
Prevotella_7	CXCL12	-0.477	0.003	0.03
Streptococcus	IL33	0.474	0.003	0.03
Limosilactobacillus	CXCL14	0.469	0.004	0.04
Streptococcus	BSG	0.460	0.004	0.04
Streptococcus	REG4	0.459	0.004	0.04
Rothia	EPHA1	0.457	0.004	0.04
Rothia	SLC39A5	0.457	0.004	0.04
Bifidobacterium	AMN	-0.461	0.005	0.04
Streptococcus	EDAR	-0.455	0.005	0.04
Streptococcus	CSF1	0.453	0.005	0.04
Solobacterium	TNFRSF11B	-0.452	0.005	0.04
Streptococcus	ALDH3A1	0.448	0.01	0.05
Streptococcus	IL17C	0.453	0.01	0.05
Bifidobacterium	CDON	-0.446	0.01	0.05
Streptococcus	NME3	0.445	0.01	0.05
Actinomyces	SLAMF7	-0.442	0.01	0.05
Rothia	PRELP	0.441	0.01	0.05
Streptococcus	CRHBP	0.441	0.01	0.05
Solobacterium	MMP10	-0.441	0.01	0.05
Solobacterium	IL33	-0.444	0.01	0.06
Prevotella_7	ITGB6	-0.443	0.01	0.06
Streptococcus	PGF	0.435	0.01	0.06

Solobacterium	PLAUR	-0.433	0.01	0.06
Limosilactobacillus	HSD11B1	0.433	0.01	0.06
Prevotella_7	CCL26	-0.432	0.01	0.06
Streptococcus	ITGB6	0.435	0.01	0.06
Actinomyces	SPRY2	0.434	0.01	0.07
Solobacterium	TFF2	-0.423	0.01	0.07
Streptococcus	PLAUR	0.420	0.01	0.07
Streptococcus	HLA.DRA	0.444	0.01	0.07
Rothia	FSTL3	0.419	0.01	0.07
Actinomyces	CXCL17	-0.419	0.01	0.07
Prevotella_7	ITGA11	-0.419	0.01	0.08
Actinomyces	WAS	0.424	0.01	0.08
Rothia	BSG	0.418	0.01	0.08
Prevotella_7	MERTK	-0.418	0.01	0.08
Solobacterium	SLAMF7	-0.417	0.01	0.08
Solobacterium	ALDH3A1	-0.417	0.01	0.08
Streptococcus	TNFSF13	0.416	0.01	0.08
Veillonella	TGFB1	0.415	0.01	0.08
Limosilactobacillus	IL17D	0.420	0.01	0.08
Limosilactobacillus	CDON	-0.414	0.01	0.08
Solobacterium	TNFSF11	0.413	0.01	0.08
Solobacterium	LAMP3	-0.413	0.01	0.08
Streptococcus	CLEC4D	0.412	0.01	0.08
Streptococcus	SLAMF7	0.412	0.01	0.08
Actinomyces	PDLIM7	0.411	0.01	0.08
Streptococcus	FLT3LG	0.410	0.01	0.08
Bifidobacterium	PTH1R	-0.410	0.01	0.08
Actinomyces	EDAR	0.409	0.01	0.09
Prevotella_7	GALNT3	-0.413	0.01	0.09
Rothia	IL20RA	0.412	0.01	0.09
Solobacterium	MYO9B	0.411	0.01	0.09
Solobacterium	CXCL8	-0.404	0.01	0.09
Veillonella	BTN2A1	0.403	0.01	0.09
Streptococcus	CRIM1	0.401	0.01	0.10
Rothia	LILRB4	0.412	0.01	0.10
Veillonella	CTSC	0.404	0.01	0.10
Actinomyces	PRKAB1	0.401	0.02	0.10
Granulicatella	LTA	0.443	0.03	0.16

Table S8. LC symptom groups sample counts.

	Oral microbiota		Sputum microbiota	
**Symptom group**	**Present**	**Absent**	**Present**	**Absent**
Cardiopulmonary	404 (53.8%)	347 (46.2%)	171 (52.8%)	153 (47.2%)
Fatigue	339 (46.9%)	384 (53.1%)	134 (44.1%)	170 (55.9%)
Mental health	220 (29.5%)	525 (70.5%)	87 (27.4%)	230 (72.6%)
Neurocognitive	331 (43.2%)	436 (56.8%)	152 (47.1%)	171 (52.9%)
GI	131 (18.6%)	572 (81.4%)	46 (15.1%)	258 (84.9%)

Table S9a. Co-occurrence network comparison for oral microbiota neurocognitive symptoms

Metric	No neurological symptoms	Neurological symptoms	Absolute difference	Similarity/Distance metric value	p-value	Notes
**Global Network (LCC)**						
Relative LCC size	0.80	0.80	0.00	–	1.00	
Clustering coefficient	0.31	0.40	0.09	–	0.27	
Modularity	0.61	0.62	0.01	–	0.75	
Positive edge percentage	74.36	82.67	8.31	–	0.14	
Edge density	0.06	0.06	0.00	–	0.81	
Natural connectivity	0.03	0.03	0.00	–	0.98	
Vertex connectivity	1.00	1.00	0.00	–	1.00	
Edge connectivity	1.00	1.00	0.00	–	2.00	
Average dissimilarity*	0.98	0.98	0.00	–	0.81	*Dissimilarity = 1 - edge weight
Average path length**	2.75	2.68	0.07	–	0.84	**Path length = Units with average dissimilarity
**Global Network (Whole Network)**						
Number of components	12.00	12.00	0.00	–	1.00	
Clustering coefficient	0.31	0.39	0.08	–	0.32	
Modularity	0.62	0.63	0.01	–	0.74	
Positive edge percentage	75.00	83.12	8.12	–	0.15	
Edge density	0.04	0.04	0.00	–	0.83	
Natural connectivity	0.02	0.02	0.00	–	0.84	
**Jaccard Index (Central Nodes)**						Jaccard index in [0,1]; 1 = perfect agreement
Degree	–	–	–	0.22	P≤: 0.23<br>P≥: 0.90	
Betweenness centrality	–	–	–	0.36	P≤: 0.71<br>P≥: 0.46	
Closeness centrality	–	–	–	0.39	P≤: 0.79<br>P≥: 0.35	
Eigenvector centrality	–	–	–	0.39	P≤: 0.79<br>P≥: 0.35	
Hub taxa	–	–	–	0.25	P≤: 0.47<br>P≥: 0.80	

LCC: Largest Connected Component. Significance codes: ***: 0.001, **: 0.01, *: 0.05, .: 0.1

Table S9b. Co-occurrence network centrality measures comparison for oral microbiota neurocognitive symptoms with hubs identified

	Centrality measure								
	Degree		Betweeness		Closeness		Eigenvector		
**Taxa**	**1: Mild**	**4: Very severe**	**1: Mild**	**4: Very severe**	**1: Mild**	**4: Very severe**	**1: Mild**	**4: Very severe**	**Hub group**
Actinomyces	0.111	0.079	0.260	0.220	0.670	0.608	0.779	0.444	Both
Treponema	0.159	0.127	0.407	0.323	0.727	0.659	1.000	0.494	Both
Bergeyella	0.095	0.063	0.229	0.096	0.597	0.563	0.400	0.151	1: Mild
Granulicatella	0.095	0.048	0.176	0.033	0.640	0.524	0.649	0.313	1: Mild
Veillonella	0.143	0.048	0.400	0.040	0.711	0.502	0.950	0.430	1: Mild
Scardovia	0.063	0.079	0.003	0.223	0.388	0.579	0.051	0.561	4: Very severe
Streptococcus	0.063	0.111	0.096	0.323	0.605	0.676	0.544	1.000	4: Very severe
Lactobacillus	0.063	0.095	0.003	0.151	0.405	0.580	0.056	0.549	4: Very severe
Dialister	0.000	0.000	0.000	0.000	0.000	0.000	0.000	0.000	None
pneumosintes	0.032	0.016	0.000	0.000	0.400	0.405	0.100	0.051	None
Selenomonas	0.000	0.000	0.000	0.000	0.000	0.000	0.000	0.000	None
Centipeda	0.000	0.000	0.000	0.000	0.000	0.000	0.000	0.000	None
Unclassified Selenomonadaceae	0.016	0.000	0.000	0.000	0.388	0.000	0.049	0.000	None
Atopobium	0.063	0.032	0.152	0.000	0.541	0.428	0.310	0.053	None
Olsenella	0.000	0.000	0.000	0.000	0.000	0.000	0.000	0.000	None
Rothia	0.063	0.095	0.082	0.091	0.550	0.625	0.332	0.844	None
Bifidobacterium	0.032	0.032	0.000	0.000	0.353	0.415	0.027	0.164	None
F0332	0.000	0.000	0.000	0.000	0.000	0.000	0.000	0.000	None
Corynebacterium	0.000	0.016	0.000	0.000	0.000	0.410	0.000	0.033	None
Cardiobacterium	0.032	0.016	0.000	0.000	0.360	0.410	0.046	0.033	None
Neisseria	0.048	0.063	0.078	0.120	0.458	0.561	0.120	0.200	None
Eikenella	0.016	0.016	0.000	0.000	0.340	0.411	0.022	0.034	None
Kingella	0.048	0.016	0.000	0.000	0.447	0.419	0.107	0.038	None
Lautropia	0.032	0.000	0.040	0.000	0.363	0.000	0.026	0.000	None
Haemophilus	0.032	0.048	0.000	0.015	0.435	0.477	0.120	0.100	None
Actinobacillus	0.000	0.000	0.000	0.000	0.000	0.000	0.000	0.000	None
Aggregatibacter	0.016	0.016	0.000	0.000	0.453	0.366	0.164	0.087	None
Lentimicrobium	0.032	0.048	0.078	0.028	0.496	0.472	0.201	0.130	None
Capnocytophaga	0.063	0.127	0.078	0.224	0.463	0.607	0.113	0.160	None
Porphyromonas	0.079	0.032	0.215	0.000	0.553	0.441	0.259	0.267	None
F0058	0.016	0.016	0.000	0.000	0.413	0.344	0.114	0.023	None
Alloprevotella	0.016	0.048	0.000	0.000	0.413	0.503	0.100	0.526	None
Prevotella	0.048	0.032	0.079	0.007	0.382	0.441	0.020	0.082	None
Prevotella_7	0.063	0.063	0.084	0.158	0.562	0.528	0.445	0.165	None
Tannerella	0.048	0.032	0.000	0.040	0.556	0.458	0.503	0.111	None
Fretibacterium	0.079	0.095	0.114	0.181	0.549	0.593	0.361	0.247	None
Fusobacterium	0.016	0.048	0.000	0.061	0.293	0.534	0.004	0.222	None

Leptotrichia	0.032	0.032	0.004	0.060	0.406	0.473	0.048	0.048	None
Catonella	0.000	0.016	0.000	0.000	0.000	0.330	0.000	0.025	None
Oribacterium	0.032	0.032	0.003	0.000	0.406	0.323	0.048	0.005	None
Stomatobaculum	0.032	0.063	0.033	0.078	0.474	0.414	0.161	0.021	None
Lachnoanaerobaculum	0.032	0.032	0.033	0.000	0.473	0.380	0.157	0.018	None
Johnsonella	0.016	0.000	0.000	0.000	0.385	0.000	0.061	0.000	None
Unclassified Clostridia UCG-014	0.016	0.016	0.000	0.000	0.385	0.277	0.062	0.005	None
Campylobacter	0.063	0.048	0.122	0.018	0.468	0.491	0.088	0.147	None
Parvimonas	0.063	0.048	0.000	0.000	0.525	0.496	0.322	0.188	None
Filifactor	0.032	0.048	0.040	0.011	0.391	0.447	0.043	0.092	None
Peptostreptococcus	0.048	0.048	0.036	0.079	0.517	0.438	0.238	0.123	None
[Eubacterium] brachy group	0.048	0.032	0.001	0.000	0.419	0.454	0.160	0.147	None
[Eubacterium] nodatum group	0.032	0.016	0.001	0.000	0.411	0.332	0.054	0.026	None
[Eubacterium] saphenum group	0.016	0.032	0.000	0.000	0.312	0.386	0.010	0.048	None
Mycoplasma	0.032	0.016	0.000	0.000	0.402	0.412	0.134	0.057	None
Solobacterium	0.016	0.032	0.000	0.000	0.294	0.323	0.004	0.005	None
Gemella	0.048	0.032	0.058	0.009	0.490	0.440	0.191	0.098	None
Abiotrophia	0.016	0.000	0.000	0.000	0.287	0.000	0.005	0.000	None
HT002	0.048	0.032	0.000	0.000	0.443	0.432	0.073	0.191	None
Limosilactobacillus	0.063	0.095	0.109	0.086	0.456	0.598	0.078	0.827	None
Unclassified Saccharimonadales	0.000	0.000	0.000	0.000	0.000	0.000	0.000	0.000	None
Candidatus Saccharimonas	0.000	0.032	0.000	0.040	0.000	0.346	0.000	0.025	None
Unclassified Saccharimonadaceae	0.079	0.016	0.042	0.000	0.596	0.440	0.598	0.108	None
TM7x	0.000	0.032	0.000	0.078	0.000	0.426	0.000	0.117	None
Unclassified Absconditabacteriales (SR1)	0.000	0.000	0.000	0.000	0.000	0.000	0.000	0.000	None
Megasphaera	0.032	0.079	0.000	0.165	0.434	0.499	0.150	0.064	None
Anaeroglobus	0.000	0.000	0.000	0.000	0.000	0.000	0.000	0.000	None

Table S10a. Co-occurrence network comparison for sputum microbiota GI symptoms

Metric	No GI symptoms	GI symptoms	Absolute difference	Similarity/Distance metric value	p-value	Notes
**Global Network (LCC)**						
Relative LCC size	0.203	0.188	0.014	–	0.960	
Clustering coefficient	0.000	0.312	0.312	–	0.094	
Modularity	0.436	0.319	0.117	–	0.499	
Positive edge percentage	85.714	66.667	19.048	–	0.179	
Edge density	0.154	0.231	0.077	–	0.425	
Natural connectivity	0.097	0.109	0.013	–	0.833	
Vertex connectivity	1.000	1.000	0.000	–	1.000	
Edge connectivity	1.000	1.000	0.000	–	1.000	
Average dissimilarity*	0.951	0.928	0.023	–	0.453	*Dissimilarity = 1 - edge weight
Average path length**	2.145	1.754	0.391	–	0.699	**Path length = Units with average dissimilarity
**Global Network (Whole Network)**						
Number of components	50.000	34.000	16.000	–	0.099	
Clustering coefficient	0.000	0.137	0.137	–	0.398	
Modularity	0.674	0.722	0.048	–	0.520	
Positive edge percentage	85.000	82.927	2.073	–	0.828	
Edge density	0.009	0.017	0.009	–	0.076	
Natural connectivity	0.016	0.016	0.000	–	0.069	
**Jaccard Index (Central Nodes)**						Jaccard index in [0,1]; 1 = perfect agreement
Degree	–	–	–	0.125	P≤: 0.02 *<br>P≥: 1.00	
Betweenness centrality	–	–	–	0.062	P≤: 0.01 **<br>P≥: 1.00	
Closeness centrality	–	–	–	0.125	P≤: 0.02*<br>P≥: 1.00	
Eigenvector centrality	–	–	–	0.125	P≤: 0.02 *<br>P≥: 1.00	
Hub taxa	–	–	–	0.067	P≤: 0.02*<br>P≥: 1.00	

LCC: Largest Connected Component. Significance codes: ***: 0.001, **: 0.01, *: 0.05, .: 0.1

Table S10b. Co-occurrence network centrality measures comparison for sputum microbiota GI symptoms with hubs identified

	Centrality measure								
	Degree		Betweeness		Closeness		Eigenvector		
**Taxa**	**1: Mild**	**4: Very severe**	**1: Mild**	**4: Very severe**	**1: Mild**	**4: Very severe**	**1: Mild**	**4: Very severe**	**Hub group**
Streptococcus	0.074	0.044	0.628	0.167	0.853	0.767	1.000	0.524	Both
Veillonella	0.044	0.000	0.628	0.000	0.806	0.000	0.866	0.000	1: Mild
Actinomyces	0.029	0.000	0.128	0.000	0.613	0.000	0.363	0.000	1: Mild
Atopobium	0.029	0.000	0.154	0.000	0.587	0.000	0.305	0.000	1: Mild
Campylobacter	0.029	0.000	0.128	0.000	0.650	0.000	0.502	0.000	1: Mild
Prevotella_7	0.044	0.000	0.474	0.000	0.740	0.000	0.604	0.000	1: Mild
Oribacterium	0.029	0.000	0.013	0.000	0.568	0.000	0.302	0.000	1: Mild
Granulicatella	0.029	0.015	0.154	0.000	0.622	0.477	0.483	0.060	1: Mild
Unclassified Selenomonadaceae	0.000	0.044	0.000	0.045	0.000	0.786	0.000	0.536	4: Very severe
Scardovia	0.000	0.044	0.000	0.136	0.000	0.820	0.000	0.527	4: Very severe
Bifidobacterium	0.000	0.059	0.000	0.273	0.000	0.815	0.000	0.503	4: Very severe
Staphylococcus	0.000	0.044	0.000	0.030	0.000	0.746	0.000	0.542	4: Very severe
Lactobacillus	0.000	0.029	0.000	0.227	0.000	0.761	0.000	0.498	4: Very severe
Limosilactobacillus	0.000	0.103	0.000	0.606	0.000	1.033	0.000	1.000	4: Very severe
Gemella	0.000	0.044	0.000	0.167	0.000	0.680	0.000	0.209	4: Very severe
Centipeda	0.000	0.000	0.000	0.000	0.000	0.000	0.000	0.000	None
Selenomonas	0.000	0.000	0.000	0.000	0.000	0.000	0.000	0.000	None
Fretibacterium	0.000	0.000	0.000	0.000	0.000	0.000	0.000	0.000	None
Neisseria	0.000	0.000	0.000	0.000	0.000	0.000	0.000	0.000	None
Kingella	0.000	0.000	0.000	0.000	0.000	0.000	0.000	0.000	None
Eikenella	0.000	0.000	0.000	0.000	0.000	0.000	0.000	0.000	None
Lautropia	0.000	0.000	0.000	0.000	0.000	0.000	0.000	0.000	None
Aggregatibacter	0.000	0.000	0.000	0.000	0.000	0.000	0.000	0.000	None
Haemophilus	0.015	0.000	0.000	0.000	0.461	0.000	0.206	0.000	None
Actinobacillus	0.000	0.000	0.000	0.000	0.000	0.000	0.000	0.000	None
Cardiobacterium	0.000	0.000	0.000	0.000	0.000	0.000	0.000	0.000	None
F0332	0.000	0.000	0.000	0.000	0.000	0.000	0.000	0.000	None
Rothia	0.015	0.015	0.000	0.000	0.559	0.554	0.434	0.202	None
Corynebacterium	0.000	0.000	0.000	0.000	0.000	0.000	0.000	0.000	None
Olsenella	0.000	0.029	0.000	0.000	0.000	0.642	0.000	0.201	None
Unclassified Absconditabacteriales (SR1)	0.000	0.000	0.000	0.000	0.000	0.000	0.000	0.000	None
TM7x	0.000	0.000	0.000	0.000	0.000	0.000	0.000	0.000	None
Unclassified Saccharimonadales	0.000	0.000	0.000	0.000	0.000	0.000	0.000	0.000	None
Candidatus Saccharimonas	0.015	0.000	0.000	0.000	0.540	0.000	0.365	0.000	None
Unclassified Saccharimonadaceae	0.000	0.000	0.000	0.000	0.000	0.000	0.000	0.000	None
Treponema	0.000	0.000	0.000	0.000	0.000	0.000	0.000	0.000	None
Mycoplasma	0.000	0.000	0.000	0.000	0.000	0.000	0.000	0.000	None

Bergeyella	0.000	0.000	0.000	0.000	0.000	0.000	0.000	0.000	None
Capnocytophaga	0.000	0.000	0.000	0.000	0.000	0.000	0.000	0.000	None
Rikenellaceae RC9 gut group	0.000	0.000	0.000	0.000	0.000	0.000	0.000	0.000	None
Lentimicrobium	0.000	0.000	0.000	0.000	0.000	0.000	0.000	0.000	None
Porphyromonas	0.000	0.029	0.000	0.000	0.000	0.679	0.000	0.382	None
Prevotella	0.000	0.000	0.000	0.000	0.000	0.000	0.000	0.000	None
Alloprevotella	0.000	0.029	0.000	0.000	0.000	0.659	0.000	0.405	None
Tannerella	0.000	0.000	0.000	0.000	0.000	0.000	0.000	0.000	None
F0058	0.000	0.000	0.000	0.000	0.000	0.000	0.000	0.000	None
Oceanivirga	0.000	0.000	0.000	0.000	0.000	0.000	0.000	0.000	None
Leptotrichia	0.000	0.000	0.000	0.000	0.000	0.000	0.000	0.000	None
Fusobacterium	0.000	0.000	0.000	0.000	0.000	0.000	0.000	0.000	None
Unclassified Clostridia vadinBB60 group	0.000	0.000	0.000	0.000	0.000	0.000	0.000	0.000	None
Unclassified Clostridia UCG-014	0.000	0.000	0.000	0.000	0.000	0.000	0.000	0.000	None
Butyrivibrio	0.000	0.000	0.000	0.000	0.000	0.000	0.000	0.000	None
Stomatobaculum	0.000	0.000	0.000	0.000	0.000	0.000	0.000	0.000	None
Lachnoanaerobaculum	0.029	0.000	0.013	0.000	0.551	0.000	0.270	0.000	None
Johnsonella	0.000	0.000	0.000	0.000	0.000	0.000	0.000	0.000	None
Catonella	0.000	0.000	0.000	0.000	0.000	0.000	0.000	0.000	None
Parvimonas	0.000	0.000	0.000	0.000	0.000	0.000	0.000	0.000	None
Filifactor	0.000	0.000	0.000	0.000	0.000	0.000	0.000	0.000	None
Peptostreptococcus	0.015	0.000	0.000	0.000	0.537	0.000	0.356	0.000	None
[Eubacterium] nodatum group	0.000	0.000	0.000	0.000	0.000	0.000	0.000	0.000	None
[Eubacterium] saphenum group	0.000	0.000	0.000	0.000	0.000	0.000	0.000	0.000	None
[Eubacterium] brachy group	0.000	0.000	0.000	0.000	0.000	0.000	0.000	0.000	None
Peptococcus	0.000	0.000	0.000	0.000	0.000	0.000	0.000	0.000	None
Solobacterium	0.000	0.000	0.000	0.000	0.000	0.000	0.000	0.000	None
Abiotrophia	0.000	0.000	0.000	0.000	0.000	0.000	0.000	0.000	None
Dialister	0.000	0.000	0.000	0.000	0.000	0.000	0.000	0.000	None
Unclassified Veillonellaceae	0.000	0.000	0.000	0.000	0.000	0.000	0.000	0.000	None
Megasphaera	0.015	0.000	0.000	0.000	0.440	0.000	0.125	0.000	None
Anaeroglobus	0.000	0.000	0.000	0.000	0.000	0.000	0.000	0.000	None

Table S11. Correlation coefficient, p value and FDR adjusted p value for correlation of sputum microbiota GI symptoms group taxa co-occurrence network hubs with inflammation related protein levels (NPX). Only showing correlations with $p < 0.05$ and absolute r^2 of >0.4.

Genus	Inflammation related protein	r^2	p-value	Adjusted p-value (FDR)
Prevotella_7	IL2	-0.439	0.02	0.19
Oribacterium	PAPPA	-0.441	0.02	0.19
Atopobium	IL1B	0.484	0.01	0.12
Actinomyces	IL33	-0.538	0.00	0.06
Campylobacter	LILRB4	-0.524	0.01	0.08
Oribacterium	LILRB4	-0.542	0.01	0.06
Streptococcus	LILRB4	0.464	0.02	0.16
Bifidobacterium	ARNT	-0.444	0.02	0.18
Atopobium	LAP3	0.431	0.03	0.21
Prevotella_7	LAP3	0.444	0.02	0.18
Limosilactobacillus	AMBN	0.438	0.03	0.19
Scardovia	IL24	-0.595	0.00	0.02
Atopobium	NBN	0.447	0.02	0.18
Prevotella_7	NBN	0.446	0.02	0.18
Scardovia	FCRL3	-0.430	0.03	0.21
Staphylococcus	FCRL3	-0.453	0.02	0.17
Limosilactobacillus	FCRL3	-0.414	0.04	0.24
Atopobium	PRKAB1	0.448	0.02	0.18
Oribacterium	PRKAB1	0.416	0.03	0.23
Atopobium	PNPT1	0.586	0.00	0.03
Prevotella_7	PNPT1	0.458	0.02	0.16
Lactobacillus	PNPT1	0.405	0.04	0.26
Atopobium	MYO9B	0.445	0.02	0.18
Prevotella_7	MYO9B	0.529	0.01	0.07
Campylobacter	IL11	-0.486	0.01	0.12
Scardovia	IL17F	0.565	0.00	0.04
Limosilactobacillus	IL17F	0.440	0.02	0.19
Gemella	IL17F	-0.439	0.02	0.19
Scardovia	SCGN	-0.501	0.01	0.10
Oribacterium	CXCL14	-0.524	0.01	0.07
Streptococcus	CXCL14	0.417	0.03	0.23
Prevotella_7	MVK	0.436	0.03	0.20
Streptococcus	WNT9A	0.441	0.02	0.19
Staphylococcus	NRTN	0.471	0.02	0.14
Bifidobacterium	DGKZ	-0.443	0.02	0.18
Prevotella_7	DGKZ	0.451	0.02	0.17
Gemella	PRKCQ	0.412	0.04	0.24
Bifidobacterium	NFATC3	-0.427	0.03	0.21
Actinomyces	GALNT3	-0.499	0.01	0.10
Campylobacter	GALNT3	-0.534	0.00	0.06
Oribacterium	GALNT3	-0.420	0.03	0.23
Streptococcus	GALNT3	0.417	0.03	0.23
Limosilactobacillus	TNF	0.409	0.04	0.25
Scardovia	TANK	-0.467	0.02	0.15
Bifidobacterium	TANK	-0.563	0.00	0.04
Gemella	TANK	0.410	0.04	0.25
Bifidobacterium	SPRY2	-0.416	0.03	0.23
Campylobacter	IL17C	-0.557	0.00	0.04
Staphylococcus	IL17C	0.405	0.04	0.26
Streptococcus	IL17C	0.461	0.02	0.15

Atopobium	YTHDF3	0.426	0.03	0.22
Actinomyces	WAS	0.407	0.04	0.25
Atopobium	WAS	0.457	0.02	0.16
Prevotella_7	WAS	0.630	0.00	0.01
Oribacterium	WAS	0.463	0.02	0.15
Streptococcus	WAS	-0.439	0.02	0.19
Oribacterium	IL17D	-0.443	0.02	0.18
Streptococcus	IL17D	0.402	0.04	0.26
Veillonella	IRAK1	0.422	0.03	0.22
Atopobium	IRAK1	0.455	0.02	0.16
Prevotella_7	IRAK1	0.459	0.02	0.16
Actinomyces	IL12RB1	-0.459	0.02	0.16
Prevotella_7	IL1RL2	-0.410	0.04	0.25
Prevotella_7	IL1A	0.442	0.03	0.20
Staphylococcus	IL1A	-0.625	0.00	0.02
Granulicatella	CSF3	-0.422	0.03	0.22
Veillonella	ITGB6	-0.414	0.04	0.24
Campylobacter	ITGB6	-0.515	0.01	0.08
Staphylococcus	JCHAIN	0.424	0.03	0.22
Staphylococcus	IFNG	0.432	0.03	0.21
Bifidobacterium	SLAMF1	0.469	0.02	0.14
Campylobacter	IL15RA	-0.479	0.01	0.13
Campylobacter	FABP9	-0.499	0.01	0.10
Scardovia	SULT2A1	0.545	0.00	0.06
Granulicatella	SULT2A1	-0.426	0.03	0.23
Bifidobacterium	NUB1	-0.477	0.01	0.13
Actinomyces	ALDH3A1	-0.681	0.00	0.00
Atopobium	ALDH3A1	-0.468	0.02	0.14
Campylobacter	ALDH3A1	-0.453	0.02	0.17
Prevotella_7	ALDH3A1	-0.613	0.00	0.02
Oribacterium	ALDH3A1	-0.609	0.00	0.02
Streptococcus	ALDH3A1	0.521	0.01	0.08
Veillonella	BID	0.577	0.04	0.25
Actinomyces	HLA.DRA	-0.499	0.01	0.11
Atopobium	TBC1D5	0.447	0.02	0.18
Prevotella_7	TBC1D5	0.411	0.04	0.24
Scardovia	DAPP1	-0.423	0.03	0.22
Prevotella_7	EDAR	0.452	0.02	0.17
Oribacterium	EDAR	0.458	0.02	0.16
Streptococcus	EDAR	-0.489	0.01	0.11
Lactobacillus	EDAR	-0.471	0.02	0.14
Limosilactobacillus	EDAR	-0.471	0.02	0.14
Prevotella_7	TRIM5	0.429	0.03	0.21
Atopobium	ITGA6	0.567	0.00	0.04
Prevotella_7	ITGA6	0.502	0.01	0.11
Veillonella	TRIM21	0.446	0.02	0.18
Atopobium	TRIM21	0.429	0.03	0.21
Prevotella_7	TRIM21	0.450	0.02	0.17
Veillonella	SAMD9L	0.417	0.03	0.23
Atopobium	SAMD9L	0.443	0.02	0.18
Prevotella_7	SAMD9L	0.463	0.02	0.15
Actinomyces	SIGLEC10	-0.436	0.03	0.20
Oribacterium	SIGLEC10	-0.404	0.04	0.26
Gemella	ISM1	-0.449	0.02	0.18
Streptococcus	PROK1	-0.457	0.02	0.16

Prevotella_7	IKBKG	0.423	0.03	0.22
Prevotella_7	NFATC1	0.428	0.03	0.21
Actinomyces	CCL26	-0.462	0.02	0.15
Campylobacter	CCL26	-0.520	0.01	0.08
Staphylococcus	CCL26	0.416	0.03	0.23
Lactobacillus	CLEC4C	0.425	0.03	0.22
Granulicatella	PSMG3	0.414	0.04	0.24
Atopobium	MAP2K6	0.490	0.01	0.11
Prevotella_7	MAP2K6	0.429	0.03	0.21
Atopobium	ARHGEF12	0.467	0.02	0.14
Prevotella_7	ARHGEF12	0.459	0.02	0.16
Oribacterium	ARHGEF12	0.430	0.03	0.21
Veillonella	MAPK9	0.473	0.01	0.14
Prevotella_7	MAPK9	0.467	0.02	0.14
Campylobacter	SELPLG	-0.415	0.03	0.24
Actinomyces	IL6	-0.425	0.03	0.22
Campylobacter	IL6	-0.520	0.01	0.08
Actinomyces	FCAR	-0.420	0.03	0.23
Campylobacter	FCAR	-0.418	0.03	0.23
Oribacterium	FCAR	-0.431	0.03	0.21
Actinomyces	NPPC	-0.436	0.03	0.20
Campylobacter	NPPC	-0.428	0.03	0.21
Actinomyces	PTX3	-0.483	0.01	0.12
Atopobium	CLEC4A	0.418	0.03	0.23
Scardovia	HSD11B1	0.441	0.02	0.19
Campylobacter	HSD11B1	-0.487	0.01	0.12
Oribacterium	HSD11B1	-0.536	0.00	0.06
Staphylococcus	HSD11B1	0.438	0.03	0.19
Streptococcus	HSD11B1	0.461	0.02	0.15
Limosilactobacillus	HSD11B1	0.654	0.00	0.01
Campylobacter	IL4R	-0.461	0.02	0.15
Granulicatella	ITGA11	0.541	0.00	0.06
Atopobium	AXIN1	0.402	0.04	0.26
Prevotella_7	AXIN1	0.474	0.01	0.13
Actinomyces	CD4	-0.536	0.00	0.06
Oribacterium	CD4	-0.445	0.02	0.18
Bifidobacterium	IL17RB	0.404	0.04	0.26
Atopobium	PIK3AP1	0.421	0.03	0.23
Prevotella_7	MGMT	0.414	0.04	0.24
Streptococcus	CLEC4G	-0.426	0.03	0.22
Atopobium	PTPRM	0.412	0.04	0.24
Streptococcus	TGFA	0.419	0.03	0.23
Bifidobacterium	IL5RA	0.418	0.03	0.23
Actinomyces	SLAMF7	-0.522	0.01	0.07
Prevotella_7	SLAMF7	-0.470	0.02	0.14
Oribacterium	SLAMF7	-0.486	0.01	0.12
Lactobacillus	SLAMF7	0.425	0.03	0.22
Atopobium	CD200	0.404	0.04	0.26
Granulicatella	CD200	0.618	0.00	0.01
Actinomyces	IL32	-0.505	0.01	0.09
Actinomyces	LIFR	-0.513	0.01	0.08
Campylobacter	LIFR	-0.403	0.04	0.26
Oribacterium	LIFR	-0.403	0.04	0.26
Atopobium	RAB6A	0.545	0.00	0.06
Prevotella_7	RAB6A	0.652	0.00	0.01

Campylobacter	CLEC4D	-0.447	0.02	0.18
Campylobacter	TLR3	0.426	0.03	0.22
Streptococcus	TLR3	-0.499	0.01	0.10
Atopobium	CXCL6	0.502	0.01	0.10
Prevotella_7	CXCL6	0.424	0.03	0.22
Actinomyces	PCDH1	-0.636	0.00	0.01
Campylobacter	PCDH1	-0.423	0.03	0.22
Oribacterium	PCDH1	-0.528	0.01	0.07
Campylobacter	CKAP4	-0.426	0.03	0.22
Oribacterium	CKAP4	-0.462	0.02	0.15
Streptococcus	CKAP4	0.452	0.02	0.17
Limosilactobacillus	CKAP4	0.478	0.01	0.13
Atopobium	FKBP1B	0.401	0.04	0.26
Veillonella	TGFB1	0.481	0.01	0.12
Atopobium	TGFB1	0.581	0.00	0.03
Lactobacillus	TGFB1	0.403	0.04	0.26
Actinomyces	CXCL17	-0.605	0.00	0.02
Campylobacter	CXCL17	-0.499	0.01	0.10
Prevotella_7	CXCL17	-0.658	0.00	0.01
Oribacterium	CXCL17	-0.702	0.00	0.00
Streptococcus	CXCL17	0.591	0.00	0.02
Limosilactobacillus	CXCL17	0.420	0.03	0.23
Gemella	CXCL17	-0.434	0.03	0.20
Atopobium	EIF4G1	0.451	0.02	0.17
Prevotella_7	EIF4G1	0.519	0.01	0.08
Campylobacter	BSG	-0.599	0.00	0.02
Streptococcus	BSG	0.408	0.04	0.25
Bifidobacterium	DNAJA2	-0.413	0.04	0.24
Prevotella_7	DNAJA2	0.438	0.03	0.19
Actinomyces	KLRB1	-0.445	0.02	0.18
Bifidobacterium	KLRB1	0.434	0.03	0.20
Staphylococcus	KLRB1	0.525	0.01	0.07
Lactobacillus	CXCL8	0.528	0.01	0.07
Gemella	CXCL8	-0.429	0.03	0.21
Actinomyces	KLRD1	-0.423	0.03	0.22
Prevotella_7	IL16	0.469	0.02	0.14
Actinomyces	NFASC	-0.510	0.01	0.09
Oribacterium	NFASC	-0.528	0.01	0.07
Streptococcus	NFASC	0.428	0.03	0.21
Lactobacillus	NFASC	0.489	0.01	0.11
Actinomyces	CD79B	-0.479	0.01	0.13
Campylobacter	CLEC7A	-0.625	0.00	0.01
Staphylococcus	CLEC7A	0.515	0.01	0.08
Limosilactobacillus	CLEC7A	0.501	0.01	0.10
Actinomyces	CD22	-0.426	0.03	0.22
Lactobacillus	CD22	0.452	0.02	0.17
Campylobacter	LAMP3	-0.429	0.03	0.21
Streptococcus	LAMP3	0.417	0.03	0.23
Veillonella	PPP1R9B	0.424	0.03	0.22
Atopobium	PPP1R9B	0.550	0.00	0.05
Prevotella_7	PPP1R9B	0.590	0.00	0.02
Oribacterium	PPP1R9B	0.413	0.04	0.24
Granulicatella	ADA	0.435	0.03	0.20
Bifidobacterium	CD160	0.404	0.04	0.26
Prevotella_7	HCLS1	0.423	0.03	0.22

Granulicatella	ADAM23	0.496	0.01	0.10
Veillonella	IL18R1	-0.450	0.02	0.17
Actinomyces	IL18R1	-0.545	0.00	0.05
Campylobacter	IL18R1	-0.458	0.02	0.16
Lactobacillus	IL18R1	0.429	0.03	0.21
Bifidobacterium	TNFRSF4	0.455	0.02	0.16
Staphylococcus	TNFRSF4	0.601	0.00	0.02
Scardovia	C1QA	0.429	0.03	0.21
Oribacterium	C1QA	-0.407	0.04	0.25
Scardovia	CCL13	-0.405	0.04	0.26
Lactobacillus	HGF	0.645	0.00	0.01
Prevotella_7	MERTK	-0.416	0.03	0.23
Oribacterium	MERTK	-0.403	0.04	0.26
Staphylococcus	MERTK	0.404	0.04	0.26
Campylobacter	FLT3LG	-0.432	0.03	0.20
Prevotella_7	FLT3LG	-0.563	0.00	0.04
Oribacterium	FLT3LG	-0.595	0.00	0.02
Limosilactobacillus	FLT3LG	0.477	0.01	0.13
Actinomyces	CLSTN2	-0.482	0.01	0.12
Campylobacter	CLSTN2	-0.531	0.01	0.07
Prevotella_7	CLSTN2	-0.445	0.02	0.18
Streptococcus	CLSTN2	0.571	0.00	0.03
Lactobacillus	CLSTN2	0.457	0.02	0.16
Actinomyces	IL12B	-0.488	0.01	0.11
Campylobacter	IL12B	-0.450	0.02	0.17
Oribacterium	IL12B	-0.445	0.02	0.18
Streptococcus	IL12B	0.468	0.02	0.14
Bifidobacterium	CDSN	0.605	0.00	0.02
Bifidobacterium	CCL11	0.456	0.02	0.16
Prevotella_7	CCL11	-0.429	0.03	0.21
Streptococcus	CCL11	0.461	0.02	0.16
Limosilactobacillus	CCL11	0.612	0.00	0.02
Gemella	CCL11	-0.474	0.01	0.13
Actinomyces	LY9	-0.426	0.03	0.22
Actinomyces	CCL20	-0.599	0.00	0.02
Campylobacter	CCL20	-0.497	0.01	0.10
Staphylococcus	CCL20	0.455	0.02	0.17
Staphylococcus	MMP1	0.436	0.03	0.20
Campylobacter	PGF	-0.500	0.01	0.10
Staphylococcus	PGF	0.539	0.00	0.06
Limosilactobacillus	PGF	0.493	0.01	0.11
Actinomyces	CCL25	-0.431	0.03	0.21
Prevotella_7	CCL25	-0.481	0.01	0.12
Actinomyces	CXCL9	-0.440	0.02	0.19
Oribacterium	CXCL9	-0.511	0.01	0.09
Scardovia	SMPDL3A	0.401	0.04	0.26
Staphylococcus	EPHA1	0.407	0.04	0.25
Atopobium	CTSC	0.497	0.01	0.13
Lactobacillus	PKLR	0.472	0.01	0.14
Actinomyces	CCL21	-0.598	0.00	0.02
Campylobacter	CCL21	-0.490	0.01	0.11
Prevotella_7	CCL21	-0.454	0.02	0.17
Oribacterium	CCL21	-0.598	0.00	0.02
Streptococcus	CCL21	0.500	0.01	0.10
Lactobacillus	CCL21	0.437	0.03	0.19

Limosilactobacillus	CCL21	0.499	0.01	0.10
Actinomyces	MMP10	-0.441	0.02	0.19
Atopobium	MMP10	-0.430	0.03	0.21
Campylobacter	MMP10	-0.420	0.03	0.23
Prevotella_7	MMP10	-0.402	0.04	0.26
Oribacterium	MMP10	-0.527	0.01	0.07
Streptococcus	MMP10	0.508	0.01	0.09
Lactobacillus	ENPP7	0.403	0.04	0.26
Actinomyces	SIGLEC1	-0.442	0.02	0.19
Lactobacillus	CD48	0.418	0.03	0.23
Granulicatella	IL18	0.454	0.02	0.17
Staphylococcus	CXCL10	0.600	0.00	0.02
Atopobium	EGF	0.403	0.04	0.26
Actinomyces	CRLF1	-0.522	0.01	0.07
Actinomyces	TNFRSF13B	-0.447	0.02	0.18
Bifidobacterium	TNFRSF13B	0.490	0.01	0.11
Staphylococcus	TNFRSF13B	0.461	0.02	0.15
Scardovia	ERBB3	0.409	0.04	0.25
Gemella	ERBB3	-0.436	0.03	0.20
Veillonella	NELL2	-0.404	0.04	0.26
Actinomyces	NELL2	-0.509	0.01	0.09
Atopobium	NELL2	-0.417	0.03	0.23
Campylobacter	NELL2	-0.430	0.03	0.21
Oribacterium	NELL2	-0.540	0.00	0.06
Streptococcus	NELL2	0.476	0.01	0.13
Atopobium	CCN2	0.415	0.03	0.24
Scardovia	MGLL	-0.844	0.00	0.01
Veillonella	FGF19	0.497	0.01	0.10
Streptococcus	CD58	0.506	0.01	0.09
Lactobacillus	CD58	0.563	0.00	0.04
Limosilactobacillus	CD58	0.432	0.03	0.21
Scardovia	PTPN6	-0.401	0.04	0.27
Streptococcus	CSF1	0.515	0.01	0.08
Lactobacillus	CSF1	0.609	0.00	0.02
Limosilactobacillus	CSF1	0.476	0.01	0.13
Actinomyces	CKMT1A_CKMT1B	-0.400	0.04	0.27
Prevotella_7	CKMT1A_CKMT1B	-0.431	0.03	0.21
Oribacterium	CKMT1A_CKMT1B	-0.454	0.02	0.17
Streptococcus	CKMT1A_CKMT1B	0.517	0.01	0.08
Atopobium	CD40	0.422	0.03	0.22
Oribacterium	ANGPTL2	-0.472	0.01	0.14
Gemella	ANGPTL2	-0.543	0.00	0.05
Streptococcus	SCG3	0.415	0.04	0.24
Atopobium	PDLIM7	0.528	0.01	0.07
Prevotella_7	PDLIM7	0.522	0.01	0.07
Bifidobacterium	SMOC2	0.466	0.02	0.15
Oribacterium	TREM2	-0.442	0.02	0.19
Streptococcus	TREM2	0.436	0.03	0.20
Streptococcus	MZB1	0.413	0.04	0.24
Actinomyces	ROBO1	-0.503	0.01	0.10
Lactobacillus	ROBO1	0.552	0.00	0.05
Prevotella_7	TNFRSF11B	-0.428	0.03	0.21
Oribacterium	TNFRSF11B	-0.488	0.01	0.12
Streptococcus	TNFRSF11B	0.420	0.03	0.23
Lactobacillus	TNFRSF11B	0.447	0.02	0.18

Limosilactobacillus	TNFRSF11B	0.448	0.02	0.18
Granulicatella	ENPP5	0.404	0.04	0.26
Streptococcus	LAIR1	0.478	0.01	0.13
Campylobacter	SIRPB1	-0.468	0.02	0.14
Oribacterium	SIRPB1	-0.457	0.02	0.16
Atopobium	PDGFB	0.462	0.02	0.15
Lactobacillus	MEGF10	0.401	0.04	0.26
Oribacterium	CRHBP	-0.504	0.01	0.09
Veillonella	LHPP	0.471	0.02	0.14
Gemella	TPP1	-0.410	0.04	0.25
Actinomyces	ADGRE2	-0.507	0.01	0.09
Streptococcus	ESM1	0.476	0.01	0.13
Actinomyces	SPON1	-0.469	0.02	0.14
Prevotella_7	SPON1	-0.433	0.03	0.20
Oribacterium	SPON1	-0.504	0.01	0.09
Streptococcus	SPON1	0.451	0.02	0.17
Oribacterium	ATP5IF1	0.457	0.02	0.16
Atopobium	SKAP2	0.409	0.04	0.25
Veillonella	CXCL1	0.403	0.04	0.26
Bifidobacterium	CXCL1	-0.426	0.03	0.22
Gemella	PRSS8	-0.449	0.02	0.17
Actinomyces	PLAUR	-0.494	0.01	0.11
Campylobacter	PLAUR	-0.533	0.01	0.06
Oribacterium	PLAUR	-0.475	0.01	0.13
Streptococcus	PLAUR	0.676	0.00	0.00
Lactobacillus	PLAUR	0.540	0.00	0.06
Limosilactobacillus	PLAUR	0.505	0.01	0.09
Bifidobacterium	PRELP	0.488	0.01	0.11
Campylobacter	MATN2	-0.434	0.03	0.20
Streptococcus	MATN2	0.649	0.00	0.01
Lactobacillus	MATN2	0.435	0.03	0.20
Limosilactobacillus	MATN2	0.403	0.04	0.26
Gemella	MATN2	-0.411	0.04	0.24
Campylobacter	LTBR	-0.490	0.01	0.11
Limosilactobacillus	LTBR	0.400	0.04	0.27
Actinomyces	CHRDL1	-0.420	0.03	0.23
Oribacterium	CHRDL1	-0.403	0.04	0.26
Streptococcus	CHRDL1	0.402	0.04	0.26
Limosilactobacillus	CCL24	0.416	0.03	0.24
Atopobium	LGMN	0.441	0.02	0.19
Lactobacillus	LGMN	0.411	0.04	0.24
Oribacterium	OSCAR	-0.458	0.02	0.16
Actinomyces	FABP1	-0.405	0.04	0.26
Bifidobacterium	FABP1	0.425	0.03	0.22
Atopobium	MPIG6B	0.417	0.03	0.23
Campylobacter	FSTL3	-0.436	0.03	0.20
Bifidobacterium	REG4	0.484	0.01	0.12
Actinomyces	TFF2	-0.537	0.00	0.06
Campylobacter	TFF2	-0.492	0.01	0.11
Oribacterium	TFF2	-0.553	0.00	0.05
Streptococcus	TFF2	0.616	0.00	0.01
Lactobacillus	TFF2	0.467	0.02	0.14
Limosilactobacillus	TFF2	0.427	0.03	0.21
Actinomyces	NME3	-0.441	0.02	0.19
Streptococcus	NME3	0.431	0.03	0.21

Lactobacillus	NME3	0.411	0.04	0.24
Gemella	NME3	-0.512	0.01	0.09

Table S12. Oral microbiota COVID-19 studies differences in relative abundance summary for genus identified as significant in our study.

Taxa (Genus)	Relative abundance	Studies
Streptococcus	↑	Soffritti et al., 2021 [7]
		Shi et al., 2022 [8]
		Miller et al., 2021 [9]
		Rafiqul Islam et al., 2022 [10]
		Gupta et al., 2022 [11]
		Cui et al., 2022 [12]
Rothia	↑	Rafiqul Islam et al., 2022 [6]
	↓	Gupta et al., 2022 [11]
		Cui et al., 2022 [12]
		Soffritti et al., 2021 [7]
Neisseria	↓	Callahan et al., 2022 [13]
		Cui et al., 2022 [12]
		Rafiqul Islam et al., 2022 [10]
		Wu et al., 2021 [14]
Veillonella	↑	Wu et al., 2021 [14]
		Soffritti et al., 2021 [7]
		Rafiqul Islam et al., 2022 [10]
		Gupta et al., 2022 [11]
		Cui et al., 2022 [12]
	↓	Shi et al., 2022 [8]
Prevotella	↑	Soffritti et al., 2021 [7]
		Gupta et al., 2022 [11]
		Cui et al., 2022 [12]
		Callahan et al., 2022 [13]
	↓	Rafiqul Islam et al., 2022 [10]
		Shi et al., 2022 [8]
Haemophilus	↑	Shi et al., 2022 [8]
	↓	Soffritti et al., 2021 [7]
		Ren et al., 2021 [15]
		Rafiqul Islam et al., 2022 [10]
		Gupta et al., 2022 [11]
		Cui et al., 2022 [12]
Fusobacterium	↓	Soffritti et al., 2021 [7]
		Ren et al., 2021 [15]
		Gupta et al., 2022 [11]
		Cui et al., 2022 [12]
Limosilactobacillus	↑	Soffritti et al., 2021 [7] (i.e. *Lactobacillus fermentum)*

Bold "↑" or "↓" indicates the direction of relative abundance for that genus in our study.

Table S13. Primer sequences for the four forward and reverse primers used for V3-V4 16S rRNA sequencing.

**Primer**	**Primer sequence (5’-3’)**
Primer 1 F	TCGTCGGCAGCGTCAGATGTGTATAAGAGACAGNCCTACGGGNGGCWGCAG
Primer 2 F	TCGTCGGCAGCGTCAGATGTGTATAAGAGACAGNNCCTACGGGNGGCWGCAG
Primer 3 F	CGTCGGCAGCGTCAGATGTGTATAAGAGACAGNNNCCTACGGGNGGCWGCAG
Primer 4 F	TCGTCGGCAGCGTCAGATGTGTATAAGAGACAGNNNNCCTACGGGNGGCWGCAG
Primer 1 R	GTCTCGTGGGCTCGGAGATGTGTATAAGAGACAGNGACTACHVGGGTATCTAATCC
Primer 2 R	GTCTCGTGGGCTCGGAGATGTGTATAAGAGACAGNNGACTACHVGGGTATCTAATCC
Primer 3 R	GTCTCGTGGGCTCGGAGATGTGTATAAGAGACAGNNNGACTACHVGGGTATCTAATCC
Primer 4 R	GTCTCGTGGGCTCGGAGATGTGTATAAGAGACAGNNNNGACTACHVGGGTATCTAATCC

Table S14. Random forest error rates for differential abundance analysis of ongoing health impairment.

Microbiota	Error rate
Oral	0.70
Sputum	0.64

Table S15. Participant respiratory comorbidities demographics stratified by sample type and ongoing health impairment cluster at 6 months post-hospitalisation.

	**Oral rinse samples (n= 722)**					**Sputum samples (n= 276)**				
	**1: Very severe**	**2: Severe**	**3: Moderate/cognitive**	**4: Mild**	**Total**	**1: Very severe**	**2: Severe**	**3: Moderate/cognitive**	**4: Mild**	**Total**
	(n= 135)	(n= 187)	(n= 147)	(n= 253)	(n= 722)	(n= 54)	(n= 58)	(n= 76)	(n= 88)	(n= 276)
**Respiratory Comorbidities**										
Chronic obstructive pulmonary disease	12 (8.9%)	7 (3.7%)	8 (5.4%)	9 (3.6%)	36 (5.0%)	4 (7.4%)	4 (6.9%)	4 (5.3%)	1 (1.1%)	13 (4.7%)
Asthma	47 (34.8%)	33 (17.6%)	18 (12.2%)	44 (17.4%)	142 (19.7%)	16 (29.6%)	9 (15.5%)	6 (7.9%)	16 (18.2%)	47 (17.0%)
Interstitial lung disease	1 (0.7%)	1 (0.5%)	0 (0.0%)	1 (0.4%)	3 (0.4%)	0 (0.0%)	1 (1.7%)	0 (0.0%)	1 (1.1%)	2 (0.7%)
Bronchiectasis	6 (4.4%)	4 (2.1%)	1 (0.7%)	5 (2.0%)	16 (2.2%)	2 (3.7%)	1 (1.7%)	0 (0.0%)	1 (1.1%)	4 (1.4%)
Obstructive sleep apnoea	10 (7.4%)	10 (5.3%)	6 (4.1%)	13 (5.1%)	39 (5.4%)	6 (11.1%)	4 (6.9%)	2 (2.6%)	3 (3.4%)	15 (5.4%)
Obesity hypoventilation syndrome	0 (0.0%)	0 (0.0%)	1 (0.7%)	0 (0.0%)	1 (0.1%)	0 (0.0%)	0 (0.0%)	1 (1.3%)	0 (0.0%)	1 (0.4%)
Pleural effusion	0 (0.0%)	2 (1.1%)	0 (0.0%)	4 (1.6%)	6 (0.8%)	0 (0.0%)	0 (0.0%)	0 (0.0%)	1 (1.1%)	1 (0.4%)
Other	6 (4.4%)	8 (4.3%)	5 (3.4%)	12 (4.7%)	31 (4.3%)	3 (5.6%)	5 (8.6%)	2 (2.6%)	1 (1.1%)	11 (4.0%)

Table S16. Top 20 taxa for sputum and oral rinse samples.

Top 20 taxa (Genus)	Oral rinse	Sputum
1	*Streptococcus*	*Streptococcus*
2	*Veillonella*	*Veillonella*
3	*Neisseria*	*Prevotella_7*
4	*Rothia*	*Neisseria*
5	*Haemophilus*	*Haemophilus*
6	*Prevotella_7*	*Rothia*
7	*Fusobacterium*	*Fusobacterium*
8	*Prevotella*	*Prevotella*
9	*Actinomyces*	*Actinomyces*
10	*Gemella*	*Porphyromonas*
11	*Porphyromonas*	*Leptotrichia*
12	*Granulicatella*	*Gemella*
13	*Leptotrichia*	*Campylobacter*
14	*Campylobacter*	*Alloprevotella*
15	*Capnocytophaga*	*Granulicatella*
16	*Alloprevotella*	*Megasphaera*
17	*Aggregatibacter*	*TM7x*
18	*Megasphaera*	*Capnocytophaga*
19	*Lautropia*	*Aggregatibacter*
20	*TM7x*	*Corynebacterium*

Supplementary references

1. Martin M. Cutadapt removes adapter sequences from high-throughput sequencing reads. EMBnetjournal. 2011;17(1):10.

2. Andrews S. FastQC: a quality control tool for high throughput sequence data 2010 [Available from: http://www.bioinformatics.babraham.ac.uk/projects/fastqc.

3. Davis NM, Proctor DM, Holmes SP, Relman DA, Callahan BJ. Simple statistical identification and removal of contaminant sequences in marker-gene and metagenomics data. Microbiome. 2018;6(1):226.

4. Xu S, Zhan L, Tang W, Wang Q, Dai Z, Zhou L, et al. MicrobiotaProcess: A comprehensive R package for deep mining microbiome. Innovation (Camb). 2023;4(2):100388.

5. Anne-Laure B, Sabine H. To adjust or not to adjust: it is not the tests performed that count, but how they are reported and interpreted. BMJ Medicine. 2024;3(1):e000783.

6. Rubin M. When to adjust alpha during multiple testing: a consideration of disjunction, conjunction, and individual testing. Synthese. 2021;199(3):10969-1000.

7. Soffritti I, D'Accolti M, Fabbri C, Passaro A, Manfredini R, Zuliani G, et al. Oral Microbiome Dysbiosis Is Associated With Symptoms Severity and Local Immune/Inflammatory Response in COVID-19 Patients: A Cross-Sectional Study. Front Microbiol. 2021;12:687513.

8. Shi YL, He MZ, Han MZ, Gui HY, Wang P, Yu JL, et al. Characterization of Altered Oropharyngeal Microbiota in Hospitalized Patients With Mild SARS-CoV-2 Infection. Front Cell Infect Microbiol. 2022;12:824578.

9. Miller EH, Annavajhala MK, Chong AM, Park H, Nobel YR, Soroush A, et al. Oral Microbiome Alterations and SARS-CoV-2 Saliva Viral Load in Patients with COVID-19. Microbiol Spectr. 2021;9(2):e0005521.

10. Rafiqul Islam SM, Foysal MJ, Hoque MN, Mehedi HMH, Rob MA, Salauddin A, et al. Dysbiosis of Oral and Gut Microbiomes in SARS-CoV-2 Infected Patients in Bangladesh: Elucidating the Role of Opportunistic Gut Microbes. Front Med (Lausanne). 2022;9:821777.

11. Gupta A, Bhanushali S, Sanap A, Shekatkar M, Kharat A, Raut C, et al. Oral dysbiosis and its linkage with SARS-CoV-2 infection. Microbiol Res. 2022;261:127055.

12. Cui GY, Rao BC, Zeng ZH, Wang XM, Ren T, Wang HY, et al. Characterization of oral and gut microbiome and plasma metabolomics in COVID-19 patients after 1-year follow-up. Mil Med Res. 2022;9(1):32.

13. Callahan N, Hattar M, Barbour T, Adami GR, Kawar N. Oral microbial taxa associated with risk for SARS-CoV-2 infection. Front Oral Health. 2022;3:886341.

14. Wu Y, Cheng X, Jiang G, Tang H, Ming S, Tang L, et al. Altered oral and gut microbiota and its association with SARS-CoV-2 viral load in COVID-19 patients during hospitalization. NPJ Biofilms Microbiomes. 2021;7(1):61.

15. Ren Z, Wang H, Cui G, Lu H, Wang L, Luo H, et al. Alterations in the human oral and gut microbiomes and lipidomics in COVID-19. Gut. 2021;70(7):1253-65.
